# In situ Synchrotron IR Microspectroscopy of CO_2_ Adsorption on Single Crystals of the Functionalized MOF Sc_2_(BDC-NH_2_)_3_**

**DOI:** 10.1002/anie.201408369

**Published:** 2014-11-07

**Authors:** Alex Greenaway, Berenice Gonzalez-Santiago, Paul M Donaldson, Mark D Frogley, Gianfelice Cinque, Jorge Sotelo, Stephen Moggach, Elenica Shiko, Stefano Brandani, Russell F Howe, Paul A Wright

**Affiliations:** EaStCHEM School of ChemistryPurdie Building, St Andrews, KY16 9ST (UK); Department of Chemistry, University of Aberdeen, Meston Buildings, King's CollegeAberdeen, AB24 3UE (UK); EaStCHEM School of Chemistry, Joseph Black BuildingWest Mains Road, Edinburgh, EH9 3JJ (UK); Diamond Light Source, Harwell Science and Innovation CampusDidcot, OX11 0DE (UK); School of Engineering, University of Edinburgh, Sanderson BuildingThe King's Buildings, Mayfield Road Edinburgh EH9 3JL (UK)

**Keywords:** analytical methods, carbon dioxide adsorption, IR spectroscopy, metal–organic frameworks, single crystals

## Abstract

Synchrotron radiation (SR) IR microspectroscopy has enabled determination of the thermodynamics, kinetics, and molecular orientation of CO_2_ adsorbed in single microcrystals of a functionalized metal–organic framework (MOF) under conditions relevant to carbon capture from flue gases. Single crystals of the small-pore MOF, Sc_2_(BDC-NH_2_)_3_, (BDC-NH_2_=2-amino-1,4-benzenedicarboxylate), with well-defined crystal form have been investigated during CO_2_ uptake at partial pressures of 0.025-0.2 bar at 298–373 K. The enthalpy and diffusivity of adsorption determined from individual single crystals are consistent with values obtained from measurements on bulk samples. The brilliant SR IR source permits rapid collection of polarized spectra. Strong variations in absorbance of the symmetric stretch of the NH_2_ groups of the MOF and the asymmetric stretch of the adsorbed CO_2_ at different orientations of the crystals relative to the polarized IR light show that CO_2_ molecules align along channels in the MOF.

MOFs exhibit properties that make them strong candidates for post combustion carbon capture, including acceptable uptakes of CO_2_ and high selectivity, regenerability, and stability to water.[1] The conventional approach to assess a potential sorbent is to acquire adsorption isotherms at relevant temperatures. However, this can be slow, even when only single component adsorption is performed—the collection of multicomponent isotherms is much more demanding.[2]

IR spectroscopy is widely used to follow the uptake of CO_2_ on powdered samples of microporous sorbents, where absorption frequencies and intensities give information on the type and amount of adsorption and variable temperature studies give enthalpies and entropies of uptake that can be compared to values determined by other methods, such as analysis of isotherms or calorimetry.[3–6] Where single crystals are available, IR microspectroscopic analysis (combined optical microscopy and IR spectroscopy) has been used for the determination of concentration-dependent diffusion parameters of hydrocarbons.[7,8] Here, we have used for the first time synchrotron-based single-microcrystal Fourier transform IR spectroscopy coupled with optical microscopy to quantify the performance of an amino-functionalized MOF, Sc_2_(BDC-NH_2_)_3_, as a carbon capture sorbent, through measurement of the heat of adsorption and the rate of desorption. This MOF shows enhanced uptake of CO_2_ over its unfunctionalized variant[9] and the amino groups in the MOF act as an internal reference for IR analysis. Furthermore, we make use of single-crystal microspectroscopy with polarized IR light to determine the orientation of adsorbed CO_2_ molecules. For crystalline samples in which the orientation of the lattice can be associated with the external morphology of the crystal, single-crystal-polarized IR spectroscopy is able to determine the orientation of molecules adsorbed within the micropores.[10] Synchrotron IR radiation (which gives an about 100-fold increase in photon flux density over Globar laboratory sources) yields a large improvement in the signal-to-noise ratio so that high quality direction-dependent polarized IR spectra can be measured for anisotropic materials. The technique has been exploited to identify the orientation of adsorbed *para*-xylene molecules in SAPO-5[11] and recently Eschenroeder et al. used it to determine the orientation of metal–amine complex templates in the zeotype STA-7.[12]

Microcrystals (80×20×20 μm^3^) of Sc_2_(BDC-NH_2_)_3_ were synthesized by a solvothermal technique (see Figure 1 a–c and the Supporting Information). Single-crystal X-ray diffraction indicated the crystals have orthorhombic *Fddd* symmetry. The most strongly developed faces of the crystals were indexed to be of the forms {011} and {001}, which are indicated on scanning electron micrographs in Figure 1 a (see also Figures S2.1 and S2.2 in the Supporting Information). The long axis of the crystals is parallel to the *a* axis, which corresponds to the channel axis. The structure (Figure 1 d) is built from rows of isolated ScO_6_ octahedra running along the *a* axis, linked by bridging carboxylate groups. This results in a series of triangular channels around 4 Å in free diameter, bounded by amino-terephthalate groups. There are two inequivalent terephthalate linkers in the structure, one disordered over two positions close to parallel to the *xy* plane and with its NH_2_ group statistically disordered over four positions (linker 1), the other inclined to the *yz* plane, and with its NH_2_ group disordered over two positions (linker 2), with the C=N bond close to parallel to the channel axis.

**Figure 1:**
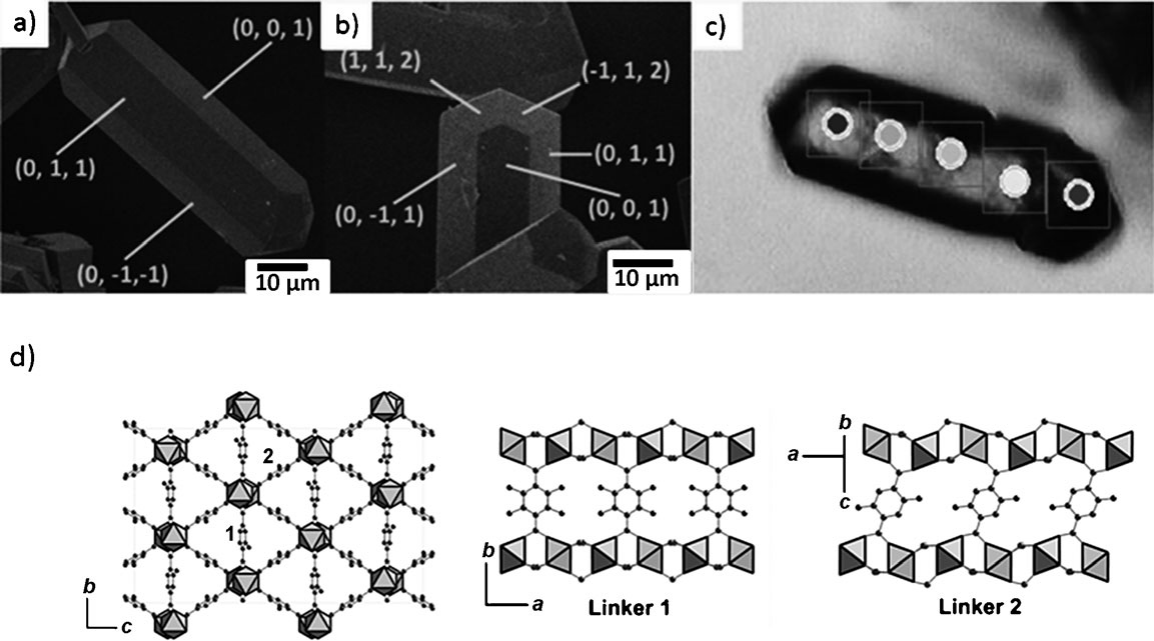
a,b) SEM of Sc_2_(BDC-NH_2_)_3_ crystals with face indices marked (10 μm scale bars shown), c) optical micrograph showing sites selected on the same crystal for IR analysis, (boxes represent 10×10 μm area from which spectra are collected). d) Framework structure of Sc_2_(BDC-NH_2_)_3_ (ScO_6_ gray octahedra, C and N atoms shown as spheres (all possible N atom positions included and H omitted).

In situ PXRD at 298–373 K and 0.1 bar CO_2_ confirmed that the structure remains orthorhombic, with very small changes in the cell parameters (see the Supporting Information). For the FTIR microspectroscopy, Sc_2_(BDC-NH_2_)_3_ crystals were loaded into a Linkam cell (path length ca. 10 mm) and this was mounted within an Bruker Hyperion 3000 IR microscope coupled to a Vertex 80 V interferometer. It was possible to identify the morphology of the crystals optically and so to determine the orientation of individual crystals. The cell allows for in situ examination under controlled gas compositions and temperature by optical microscopy and IR microspectroscopy.

The first set of experiments investigated the kinetics and thermodynamics of CO_2_ adsorption on single crystals of Sc_2_(BDC-NH_2_)_3_ under a gas flow of 10 % CO_2_ in helium at temperatures relevant to temperature swing adsorption processes. Sites were selected on crystals under the microscope, bearing in mind that the incident beam size giving spectra with good signal-to-noise ratio (above 1000) was approximately 10×10 μm^2^. MicroFTIR spectra were measured in transmission using a 15× objective/condenser with 512 scans (80 s) per point, at 2 cm^−1^ resolution. More than 20 sites were chosen both on different crystals and also on the same crystal (Figure 1 c). Spectra were taken using non-polarized IR radiation at 298 K on crystals pre-heated to 400 K, all in a dry He flow. The spectra were of such good quality that the asymmetric and symmetric NH_2_ stretches were clearly visible, at 3513 and 3396 cm^−1^ respectively. Switching the gas flow to 10 % CO_2_ in He enabled spectra to be measured in the presence of gas-phase CO_2_. The background signal from gas-phase CO_2_ could be subtracted successfully. The asymmetric stretching vibration of adsorbed CO_2_ occurred at 2333 cm^−1^, 15 cm^−1^ below the gas-phase value. It was accompanied by a weak shoulder at about 2324 cm^−1^. The relative intensity of the lower frequency shoulder remained constant independent of CO_2_ loading (see the Supporting Information for the spectra). Switching to pure He resulted in a rapid decrease of the adsorbed CO_2_ signal to 1 % of its original value within 2 minutes, indicating rapid diffusion within the MOF. This was confirmed by zero length column experiments on a 13 mg sample, which follow desorption of CO_2_ from a thin wafer of powder. These showed that desorption is 99 % complete within a few tens of seconds at the flow rates used in the IR experiment (see the Supporting Information for details). Analysis shows that although the desorption is too fast to permit calculation of the diffusional time constant, an upper bound of R^2^/D of 5.3 s can be estimated,[13] corresponding to a lower limit for diffusivity along the channels, *D*, of 3×10^−10^ m^2^ s^−1^, higher than measured by different methods for zeolite 5A.[14]

In the light of this, an experiment was conducted in which spectra were measured on the selected crystal sites in the presence of 10 % CO_2_ as the temperature was raised stepwise from 298 to 393 K. The sample was allowed to equilibrate for approximately 5 minutes at each temperature before unpolarized IR spectra were collected. As the temperature was increased the magnitude of the adsorbed CO_2_ asymmetric stretch decreased relative to the size of the peaks associated with the NH_2_ stretches (see Figure 2 for a series of spectra from a single point). This indicates that the concentration of adsorbed CO_2_ decreases with increasing temperatures, as expected for exothermic adsorption. Subsequently, similar series of experiments were performed as the partial pressure of CO_2_ was adjusted to 0.025, 0.050, and 0.2 bar. Above this pressure the absorption from gas-phase CO_2_ precluded accurate measurement.

**Figure 2:**
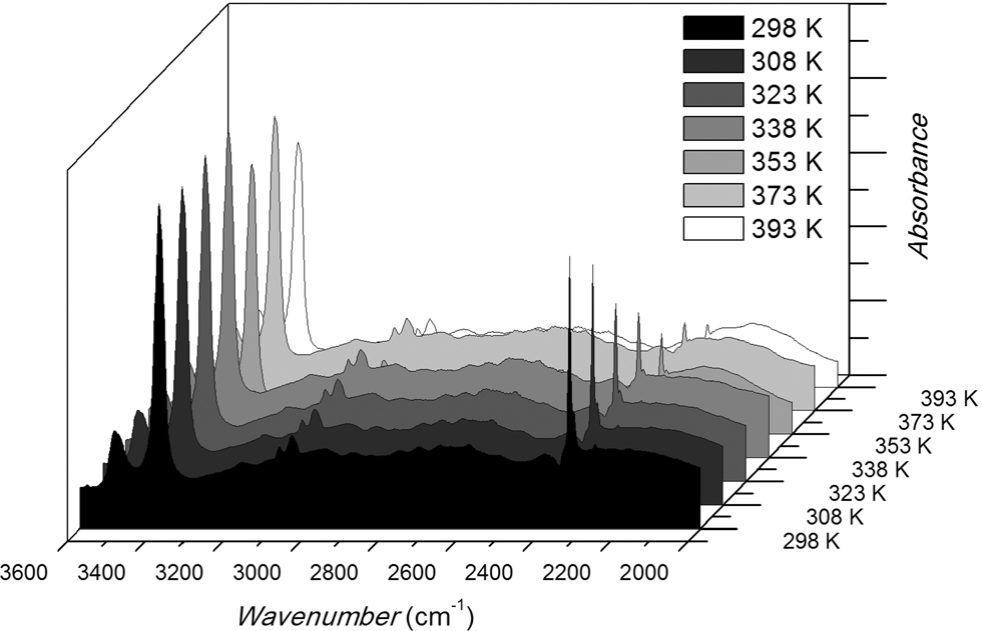
Series of spectra taken from an isobar from a single site of a single Sc_2_(BDC-NH_2_)_3_ crystal. As temperature increases the magnitude of the adsorbed CO_2_ asymmetric stretch at 2335 cm^−1^ wavenumbers decreases relative to the NH_2_ symmetric and asymmetric stretches.

IR spectra were analyzed using the OPUS 7.2 software.[15] Integrals were calculated for the NH_2_ symmetric and asymmetric stretches and the CO_2_ asymmetric stretch. The combined integral associated with the NH_2_ stretches was used as an internal reference to quantify the uptake of CO_2_, by calculating the ratio of the integral of the CO_2_ asymmetric stretch to that of the NH_2_ region. At 100 mbar CO_2_ and 298 K the specific uptake of CO_2_ on Sc_2_(BDC-NH_2_)_3_ was known from gravimetric measurements to be 0.393 mmol g^−1^. Measured peak area ratios were used to estimate the uptakes at each temperature in each isobar. Figure 3 a shows the calculated uptake at 0.1 bar on the single crystal shown in Figure 1 c. The variation of the uptakes at 0.1 bar measured by IR spectroscopy on single crystals and from gravimetric isotherms (see the Supporting Information) shows good agreement. Figure 3 b shows the uptake from several crystals for isobars at 0.025, 0.05, 0.1, and 0.2 bar CO_2_.

**Figure 3:**
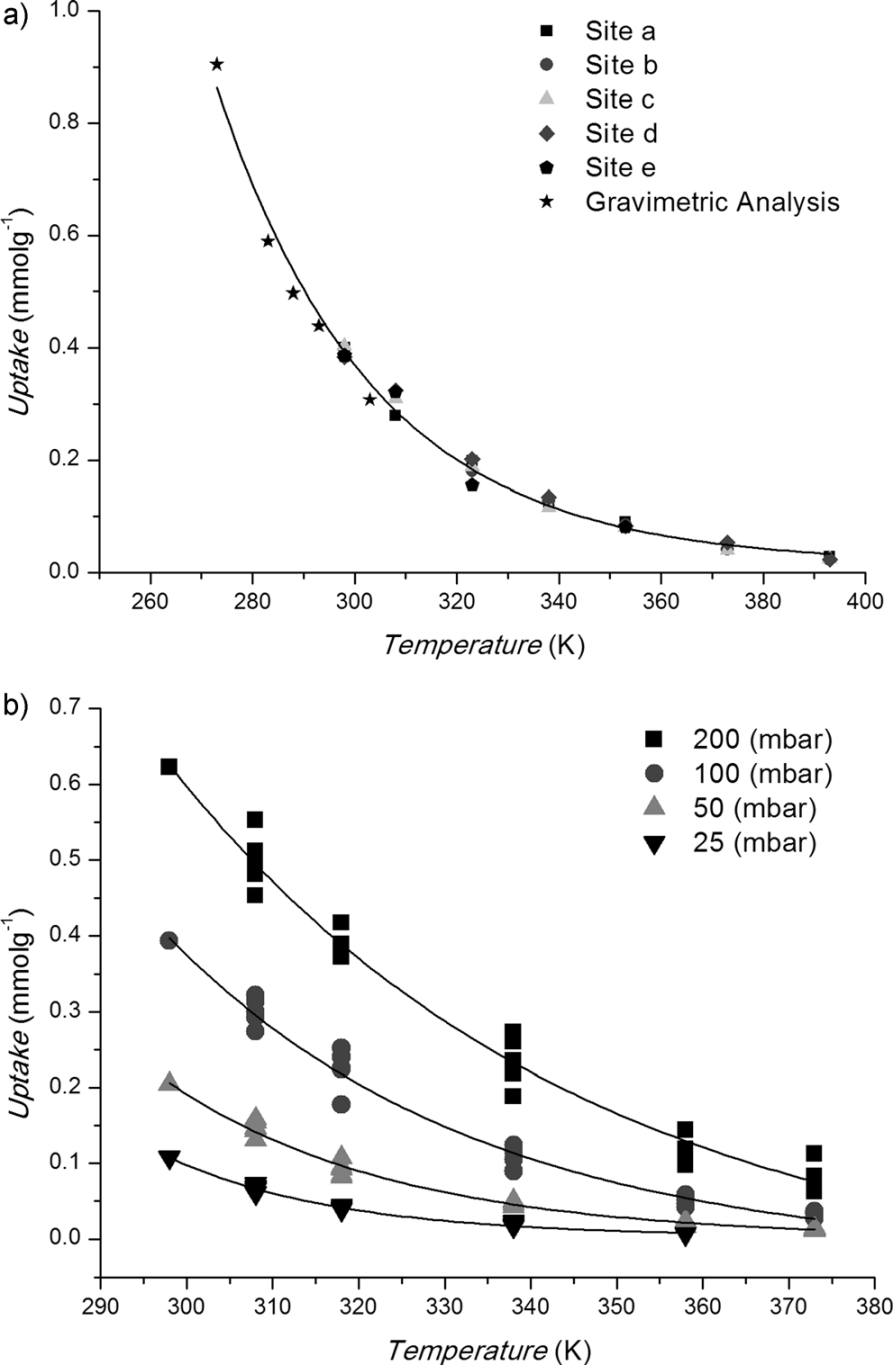
a) CO_2_ isobar (*P*
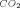
=0.1 bar), measured on 5 sites (Figure 1 c) on a single crystal at 298–393 K (square, site a; circle, b; triangle, c; diamond, d; pentagon, e). Comparison between uptakes from single crystal IR data and conventional gravimetric adsorption (stars), fitted by an exponential curve calculated assuming a heat of adsorption of 31 kJ mol^−1^. b) CO_2_ isobars (*P*
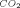
=25, 50, 100, 200 mbar).

The uptakes were used to calculate a fractional coverage and hence a value for the equilibrium constant *K* for adsorption according to the equilibrium, CO_2_(g)+S↔CO_2_(ads), where S is the adsorption site. Full coverage was taken as 5.1 mmol g^−1^.[9] The heat of adsorption for CO_2_ in Sc_2_(BDC-NH_2_)_3_ was calculated using these single-crystal data to be 31±2 kJ mol^−1^ from plots of ln*K* vs. 1/*T* (see the Supporting Information). This agrees with the isosteric heats calculated from a series of adsorption isotherms at different temperatures, the average of which was 31±3 kJ mol^−1^. The heats of adsorption on the amino-functionalized material are higher than those reported previously on Sc_2_BDC_3_ (23 kJ mol^−1^).[16] This is the result of interactions of CO_2_ with NH_2_.

IR spectroscopy can also be sensitive (in terms of frequency shifts) to molecular interactions. Upon CO_2_ adsorption on Sc_2_(BDC-NH_2_)_3_ the peak maxima for the symmetric and asymmetric NH_2_ stretches shift by approximately 5 cm^−1^ to lower values (3396 to 3392 cm^−1^; 3513 to 3508 cm^−1^, respectively) suggesting a weak additional interaction between the NH_2_ groups and CO_2_.

It is therefore possible to determine the rates, heat, and nature of adsorption of CO_2_ on single crystals using IR microspectroscopy under realistic conditions. While these could be obtained from bulk samples, the microscopic approach can measure variation between different crystals. Furthermore, and importantly, synchrotron IR single-crystal microspectroscopy can give information inaccessible to bulk methods, in particular by the use of a polarized IR beam.

To understand the adsorption of CO_2_ onto the MOF, non-polarized and polarized IR spectra were collected from crystals orientated parallel or perpendicular to the holographic ZnSe wire grid polarizers used to define the electric field direction of the IR beam being detected. The crystals of Sc_2_(BDC-NH_2_)_3_ have well-defined facets, so that it is possible from the optical image to establish the crystal orientation with respect to the polarized beam: crystals were found to settle in one of three ways: with their (001), (011) or (010) plane parallel to the window (and perpendicular to the plane of the polarized IR light). Schematic representations of these orientations (illustrated by SEM micrographs) are shown in Figure 4. Spectra were collected at 298 K using unpolarized IR light and also IR light polarized parallel and perpendicular to the crystal length, on activated crystals and in 0.1 bar CO_2_. Integrals were calculated for the NH_2_ symmetric and the CO_2_ asymmetric stretches, to enable details of the orientation of adsorbed molecules in the functionalized framework to be measured (see the Supporting Information).

**Figure 4:**
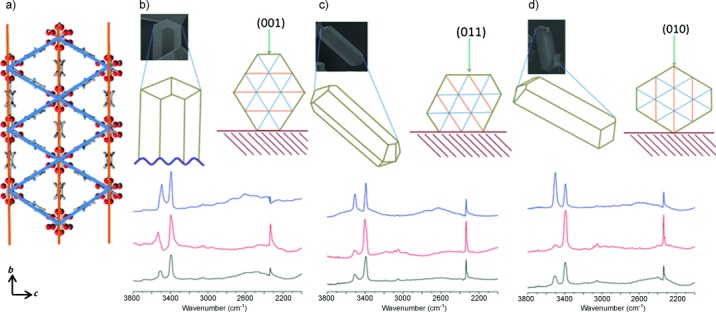
a) The framework of Sc_2_(BDC-NH_2_)_3_ viewed down the *a* axis, which corresponds to the morphologically longest direction of the crystals. Orange (type 1 linker) and blue (type 2 linker) lines indicate the projections of the different linkers. b,c,d) Scanning electron micrographs of crystals in similar orientations to those studied, and schematic representations, for crystals lying with (001), (011) and (010) planes parallel to the window of the cell, respectively. The spectra (b,c,d) are of unpolarized spectra of the MOF with adsorbed CO_2_ (black), spectra with direction of polarization parallel (red) and perpendicular (blue) to long morphological axis of a crystal lying on (001), (011), and (010) faces, respectively.

In each case the polarized FTIR spectra reveal that the absorbance of the NH_2_ symmetric stretch increases when the polarization is parallel to the long direction of the crystal, indicating that the C_Ph_=N bonds aligned along the channels. Very similar NH_2_ absorbance behavior is observed in crystals with and without adsorbed CO_2_, suggesting that there is no significant re-orientation of the ligands upon uptake of CO_2_. Furthermore, the CO_2_ asymmetric stretch intensity is enhanced when the IR light is polarized along the crystal length, indicating that the average alignment of the CO_2_ molecules is oriented along the channels.

A more quantitative estimation of the CO_2_ alignment can be made if the structure of Sc_2_(BDC-NH_2_)_3_ is considered (Figure 1 d and Figure 4). Intensities of polarized SR IR spectra vary strongly according to orientation, but by comparing the relative enhancement or suppression of the CO_2_ asymmetric stretch with respect to the symmetric NH_2_ stretch (which is expected to give a dipole change parallel to the C_Ph_=N bond) it is possible for each of the projections to determine whether the CO_2_ molecules are aligned more or less closely to the channel axis than the net C_Ph_-N vector. (It is not possible to locate the amine hydrogen atoms by diffraction, so we have not analyzed directional information from the asymmetric stretch.) The C_Ph_-N vectors for all of the possible orientations of amino groups on the terephthalate ligands were measured from projections of the single-crystal structure down [001], [011] and [010], and a weighted average of their deviation from the [100] axis was determined (see the Supporting Information). The net C_Ph_-N vector is close to parallel to the channel axis in the MOF (17° off-parallel viewed down [001], 13° off-parallel viewed down [011], and 11° off-parallel for [010]). The intensity of the asymmetric stretch of the linear O=C=O molecule is enhanced relative to the NH_2_ symmetric stretch using IR light polarized along the crystal length when viewed down [001] (by ca. 90 %) and [011] (by 23 %) but is suppressed when viewed down [010] (by 17 %). The projection of the CO_2_ molecule is therefore more closely aligned to the channel axis than 17° when viewed down [001] and makes a larger angle than 11° when viewed down [010], in effect enabling triangulation of the relative orientation of the CO_2_ molecules’ orientation in the channel, which is on average closer than approximately 20° to the channel axis.

A powerful new SR FTIR microspectroscopic technique has been developed which permits the rapid analysis of adsorption by porous materials under flowing gas conditions relevant to carbon capture. Investigations on an amino-functionalized MOF give a heat of adsorption and desorption rates on a single crystal and polarized IR light has been used to show a strong orientation dependency of adsorbed CO_2_ molecules within the pores of Sc_2_(BDC-NH_2_)_3_, even at rather low uptakes, a result which is experimentally difficult to attain by other means. Whereas previous diffraction measurements have located adsorbed CO_2_ on MOFs at room temperature at loadings down to approximately 1 mmol g^−1^,[17] these IR measurements give acceptable signal-to-noise ratios for the asymmetric stretch of adsorbed CO_2_ at <0.1 mmol g^−1^ (fractional site occupancies <0.015). Furthermore, the method has the potential to follow temporal and spatial variation over a single crystal and so to determine adsorbate diffusivities. Although the diffusion of CO_2_ in Sc_2_(BDC-NH_2_)_3_ is too fast to permit this, there are many examples of more slowly diffusing, IR-active molecules (e.g. H_2_O, N_2_O, small hydrocarbons or organics) in other porous solids than can be studied in this way, even when part of adsorbing mixtures, especially when developments in full field FTIR imaging using synchrotron radiation IR brilliance can offer faster acquisition and a larger field of view (see the Supporting Information).[18]

The results reported here confirm the potential of amino-functionalized MOFs as carbon capture materials and indicate that in situ single-crystal gas adsorption and polarized IR microspectroscopy can rapidly provide unique insights into single- and eventually multi-component adsorption processes.

## Notes

Supporting information for this article including details of the in situ IR experiments, single-crystal and powder X-ray diffraction, zero length column chromatography, and gravimetric analysis are given in the Supporting Information is available on the WWW under http://dx.doi.org/10.1002/anie.201408369.

## References

[1] Sumida K, Rogow DL, Mason JA, McDonald TM, Bloch ED, Herm ZR, Bae TH, Long JR (2012). Chem. Rev.

[2a] Li B, Kaye SS, Riley C, Greenberg D, Galang D, Bailey MS (2012). ACS Comb. Sci.

[2b] Bae TH, Hudson MR, Mason JA, Queen WL, Dutton JJ, Sumida K, Micklash KJ, Kaye SS, Brown CM, Long JR (2013). Energy Environ. Sci.

[3] Nijem N, Canepa P, Kong L, Wu H, Li J, Thonhauser T, Chabal YJ (2012). J. Phys. Condens. Matter.

[4] Arean CO, Bibiloni GF, Delgado MR (2012). Appl. Surf. Sci.

[5] Llewellyn PL, Bourelly S, Vagner C, Heymans N, Leclerc H, Ghoufi A, Bazin P, Vimont A, Daturi M, Devic T, Serre C, De Weireld G, Maurin G (2013). J. Phys. Chem. C.

[6] Lozinska MM, Mangano E, Mowat JPS, Shepherd AM, Howe RF, Thompson SP, Parker JE, Brandani S, Wright PA (2012). J. Am. Chem. Soc.

[7a] Chmelik C, Kärger J (2010). Chem. Soc. Rev.

[7b] Kärger J, Binder T, Chmelik C, Hibbe F, Krautscheid H, Krishna R, Weitkamp J (2014). Nat. Mater.

[8] Pantatosaki E, Megariotis G, Pusch A-K, Chmelik C, Stallmach F, Papadopoulus GK (2012). J. Phys. Chem. C.

[9] Mowat JPS, Miller SR, Griffin JM, Seymour VR, Ashbrook SE, Thompson SP, Fairen-Jimenez D, Banu A-M, Düren T, Wright PA (2011). Inorg. Chem.

[10] Stavitski E, Weckhuysen BM (2010). Chem. Soc. Rev.

[11] Schüth F, Demuth D, Zibrowius B, Kornatowski J, Finger G (1994). J. Am. Chem. Soc.

[12] Eschenroeder ECV, Turrina A, Picone AL, Cinque G, Frogley MD, Cox PA, Howe RF, Wright PA (2014). Chem. Mater.

[13] Brandani S, Ruthven DM (1996). Adsorption.

[14a] Ruthven DM (1993). Zeolites.

[14b] Kärger J, Pfeifer H, Stallmach F, Feoktistova N, Zhdanov SP (1993). Zeolites.

[15] OPUS software 7.0, Bruker Optik GmbH, Germany, **2011**

[16] Miller SR, Wright PA, Devic T, Serre C, Férey G, Llewellyn PL, Denoyel R, Gaberova L, Filinchuck Y (2009). Langmuir.

[17] Wriedt M, Sculley JP, Yakovenko AA, Ma Y, Halder GJ, Balbuena PB, Zhou H-C (2012). Angew. Chem. Int. Ed.

[18] Quaroni L, Zlateva T, Sarafimov B, Kreuzer HW, Wehbe K, Hegg EL, Cinque G (2014). Biophys. Chem.

