# Supplementary material for: In situ Synchrotron IR Microspectroscopy of CO2 Adsorption on Single Crystals of the Functionalized MOF Sc2(BDC-NH2)3
Source: Angew Chem Int Ed Engl. 2014 Nov 7;53(49):13483–7. doi: 10.1002/anie.201408369 (PMC4501324; doi:10.1002/anie.201408369)
Supplement: Supplementary file 1 [file anie0053-13483-sd1.pdf]

Angewandte Chemie
Eine Zeitschrift der Gesellschaft Deutscher Chemiker

Supporting Information

© Wiley-VCH 2014

69451 Weinheim, Germany

In situ Synchrotron IR Microspectroscopy of CO_2 Adsorption on Single Crystals of the Functionalized MOF $Sc_2(BDC\text{-}NH_2)_3$**

Alex Greenaway, Berenice Gonzalez-Santiago, Paul M. Donaldson, Mark D. Frogley, Gianfelice Cinque, Jorge Sotelo, Stephen Moggach, Elenica Shiko, Stefano Brandani, Russell F. Howe, and Paul A. Wright**

anie_201408369_sm_miscellaneous_information.pdf

Supporting Information

Contents

S1 Synthesis and general characterisation of $Sc_2(BDC\text{-}NH_2)_3$ 3

S2 Single crystal diffraction and face indexing of single crystals of $Sc_2(BDC\text{-}NH_2)_3$ 7

S3 In situ gas adsorption variable temperature PXRD 11

S4 CO_2 Zero length column (ZLC) experiments 13

S4a Technique 13

S4b Experimental procedure 13

S4c Results 14

S5 Single crystal micro FTIR:- General notes 15

S5a Data Collection 15

S5b Analysis of spectra 16

S6 Variable Temperature in situ gas adsorption Single crystal micro FTIR analysis 19

S6a Procedure 19

S6b Results of single crystal analysis for 0.1 bar CO_2 (Crystal sites 1-5) 19

S6c Analysis of spectra for 0.1 bar CO_2 25

S7 Variable Temperature In situ-gas adsorption Single crystal micro FTIR analysis, CO_2 isobars

S7a Procedure 29

S7b Results 29

Sites 1-4, spectra:- 30

S7c Heat of adsorption analysis (200, 100, 50, 25 mbar) 34

S8 Gravimetric CO_2 adsorption isotherms and isosteric heats 38

S9 In situ single crystal polarisation experiments 38

S9a Samples with no CO_2 43
S9b Samples with CO_2 (Crystals 1-6) 44
S9c Analysis of spectra 51
S9d Crystal projections onto (011), (001) and (010) 56
S9e Physical interpretation of spectra and crystal projections:- Positioning adsorbed CO_2 60
S10 Synchrotron IR Microcrystal Spectroscopy Developments..61
S11 Notes and References..63

Appended cif file

S1. Synthesis and general characterisation of $Sc_2(BDC\text{-}NH_2)_3$

The synthesis of $Sc_2(BDC\text{-}NH_2)_3$ was performed via a solvothermal route using scandium chloride, prepared from scandium oxide (Sc_2O_3, 99.999%, Stanford Materials Corporation)[1] as the scandium source. Scandium chloride ($ScCl_3$, 0.65 mmol, 0.43 ml of 1.5 M aqueous solution) and amino-terephthalic acid (0.21 g, 1.16 mmol) (Aldrich, >98%) were mixed in DMF (1.0 ml) and water (4.0 ml). The homogenized reaction mixture was transferred to a Teflon-lined Parr autoclave, sealed and heated at 403 K for 12 h. After cooling, solid material was collected by filtration, washed with ethanol and dried at 343 K overnight. Identification of the product phases was achieved by laboratory source powder X-ray diffraction. Comparison of the experimental profile with a simulated pattern derived from the single crystal structure indicated the material was phase pure (fig. S1-1). Thermogravimetric analysis was performed at 10 K min^{-1} under flowing air using a Netzsch TG 209 instrument showing results consistent with those previously reported for $Sc_2(BDC\text{-}NH_2)_3$ (fig S1-2).[2]

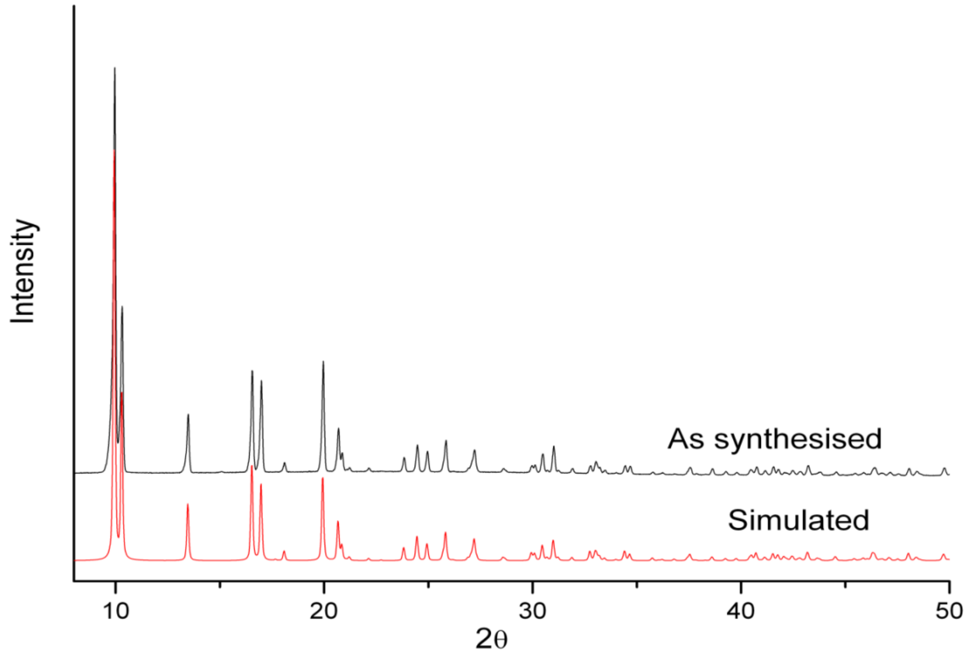

Figure S1.1 Comparision of experimental PXRD of as-synthesised $Sc_2(NH_2\text{-}BDC)_3$ (black) with that simulated for $Sc_2(NH_2\text{-}BDC)_3$ (red).

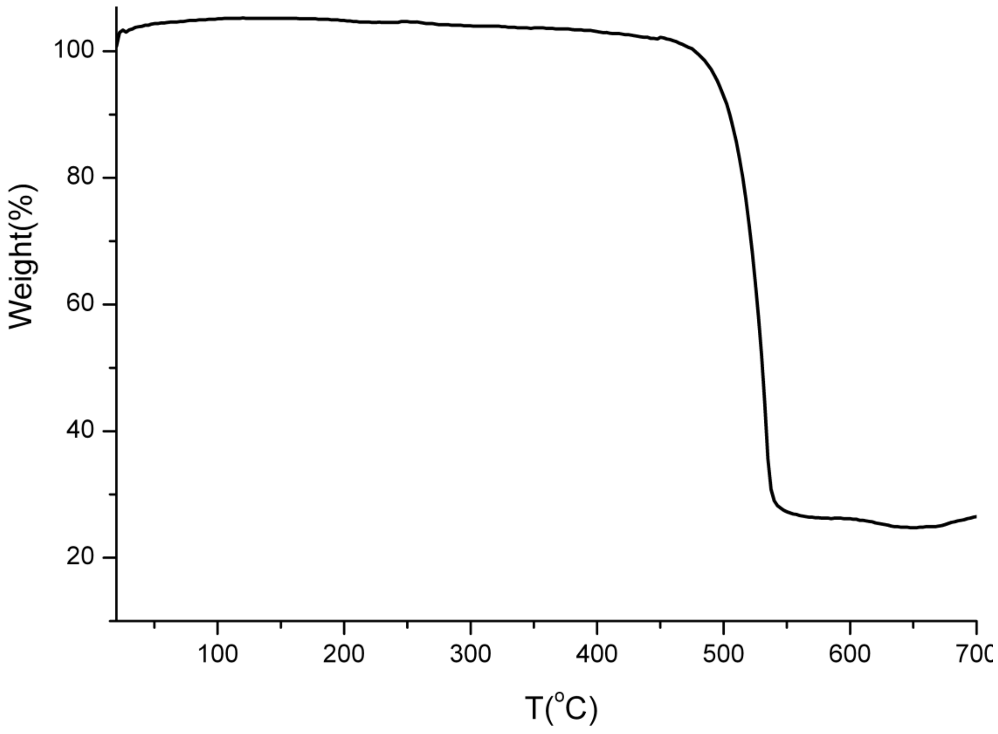

Figure S1.2 Thermogravimetric analysis of $Sc_2(BDC\text{-}NH_2)_3$ in flowing air.

SEM and EDX analysis of crystals

SEM micrographs of samples of as-prepared $Sc_2(BDC\text{-}NH_2)_3$ obtained on a Jeol JSM 5600 scanning electron microscope show that the crystals exhibit a well-defined and clearly identifiable anisotropic morphology with a single long axis and a pseudohexagonal cross section (Figure S1.3(a)-(f)). The size of the crystals is fairly uniform, with typical crystals having dimensions of 20-30 µm in width (across cross section) and 60 - 100 µm in length.

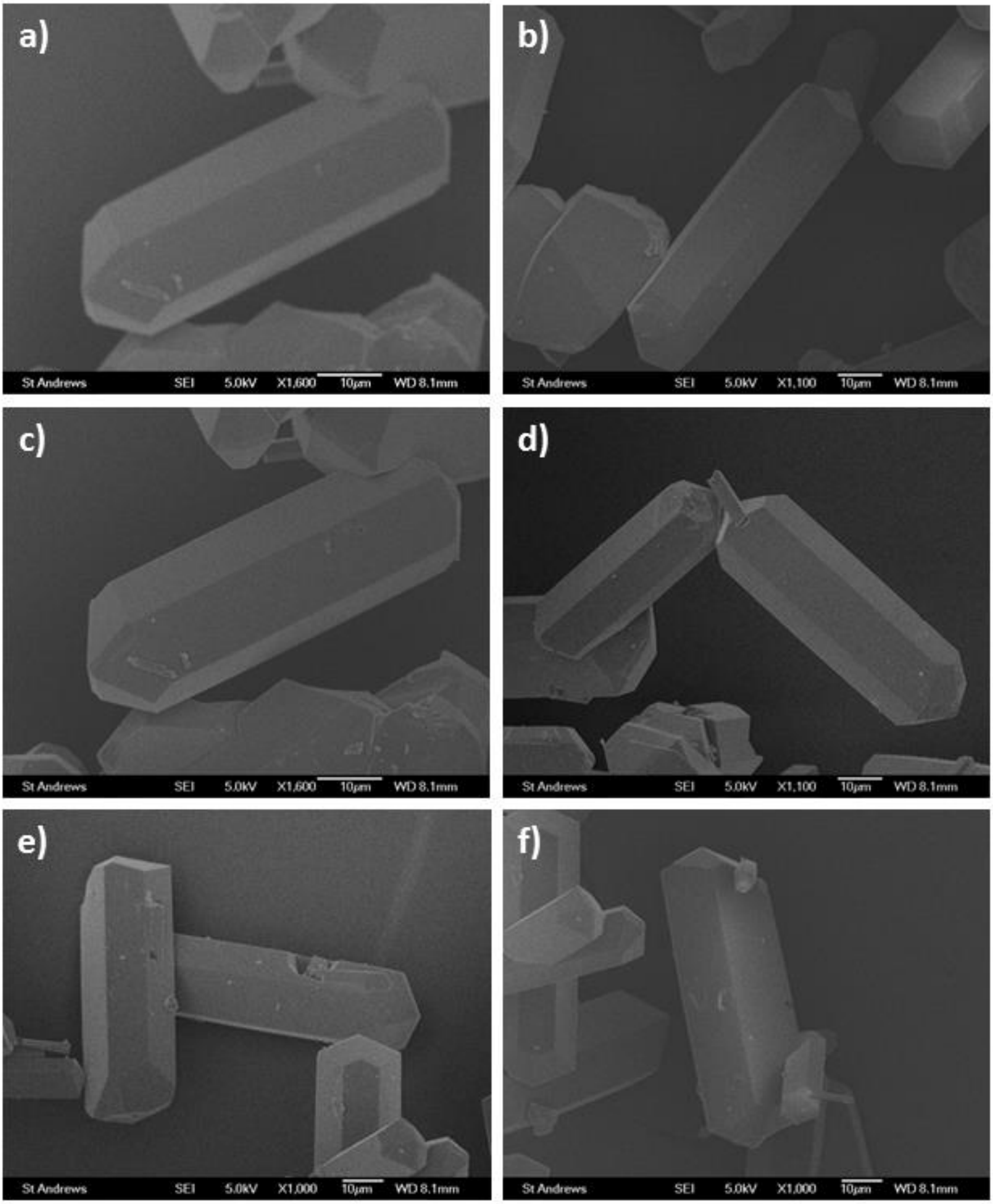

Figure S1.3 SEM secondary electron micrographs of samples of as-prepared $Sc_2(BDC\text{-}NH_2)_3$

EDX analysis on crystals of $Sc_2(BDC\text{-}NH_2)_3$ were conducted on a Jeol JSM 5600 SEM, with integrated EDX (Energy Dispersive X-rays) analytical system. EDX analysis confirmed the presence of metal in the MOF: Sc expected 14.3 wt%; 17.7% measured; C expected 45.9 wt%; 44.5 % wt measured, N expected 6.7 wt% measured 7.5 wt. % (Fig S1.4).

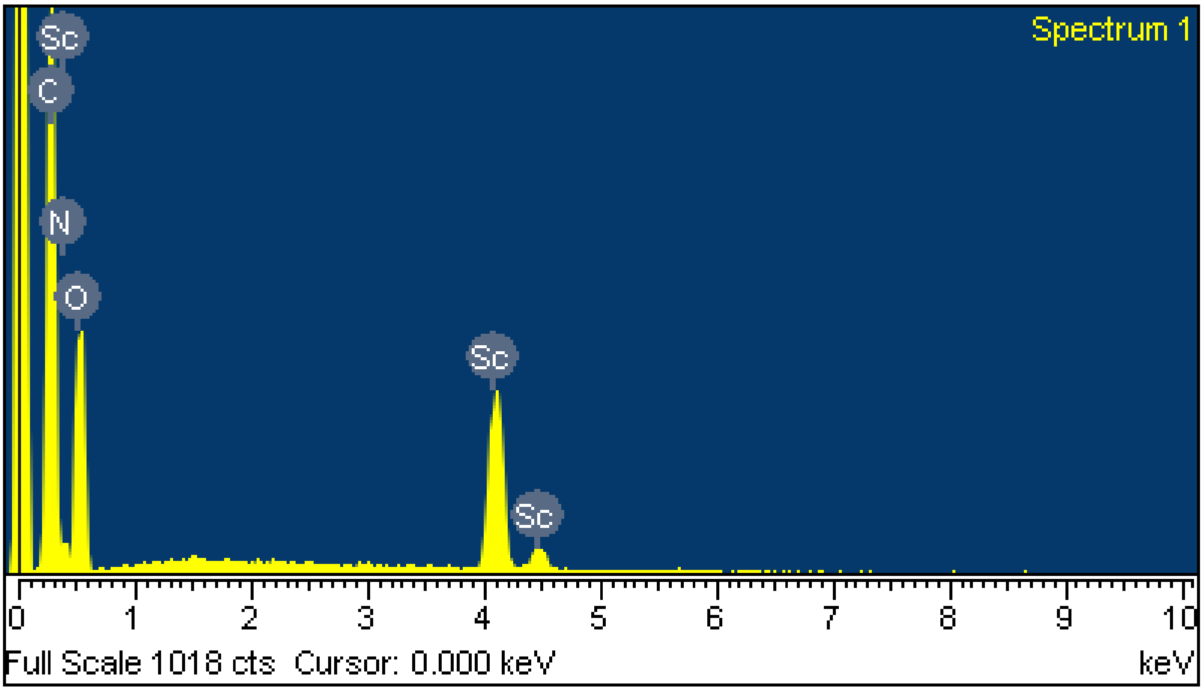

Figure S1.4 EDX spectrum of $Sc_2(NH_2\text{-}BDC)_3$

S2 Single crystal diffraction and face indexing of single crystals of $Sc_2(BDC\text{-}NH_2)_3$

The structure of $Sc_2(BDC\text{-}NH_2)_3$ was solved by single crystal diffraction in the space group *Fddd* at 120 K, giving structural details not available from the structure determined from powder diffraction. Details of the structure solution are given in the attached cif file.

There are two symmetrically-inequivalent aminoterephthalate groups. Linker 1 runs (carboxylate to carboxylate) parallel to the *y* axis, and for this ligand the phenyl ring exists in two configurations, each tilted a few degrees from the *xy*-plane. For each of these two configurations, the amino group N is found in two possible locations, at 0.25 occupancy, and located so that the amino group N can form a N-H – O bond with the nearest carboxylate O atom. Note that the carboxylate group is also tilted away from the *xy*-plane, so that the NH_2 group is found on the phenyl C atoms that permit the closest O – H(N) approach (2.1 Å). The second linker, 2, joins chains of ScO_6 octahedra, and runs oblique to the yz-plane, with NH_2 groups disordered over two symmetrically equivalent positions, again in locations that permit close (N)H – O distances of approach (2.0 Å) so that the CN bonds runs close to parallel to the *z* axis (channel direction).

Amino groups are disordered over different C atoms of the linkers: a possible arrangement of amino N atoms is shown below (H atoms omitted for clarity)

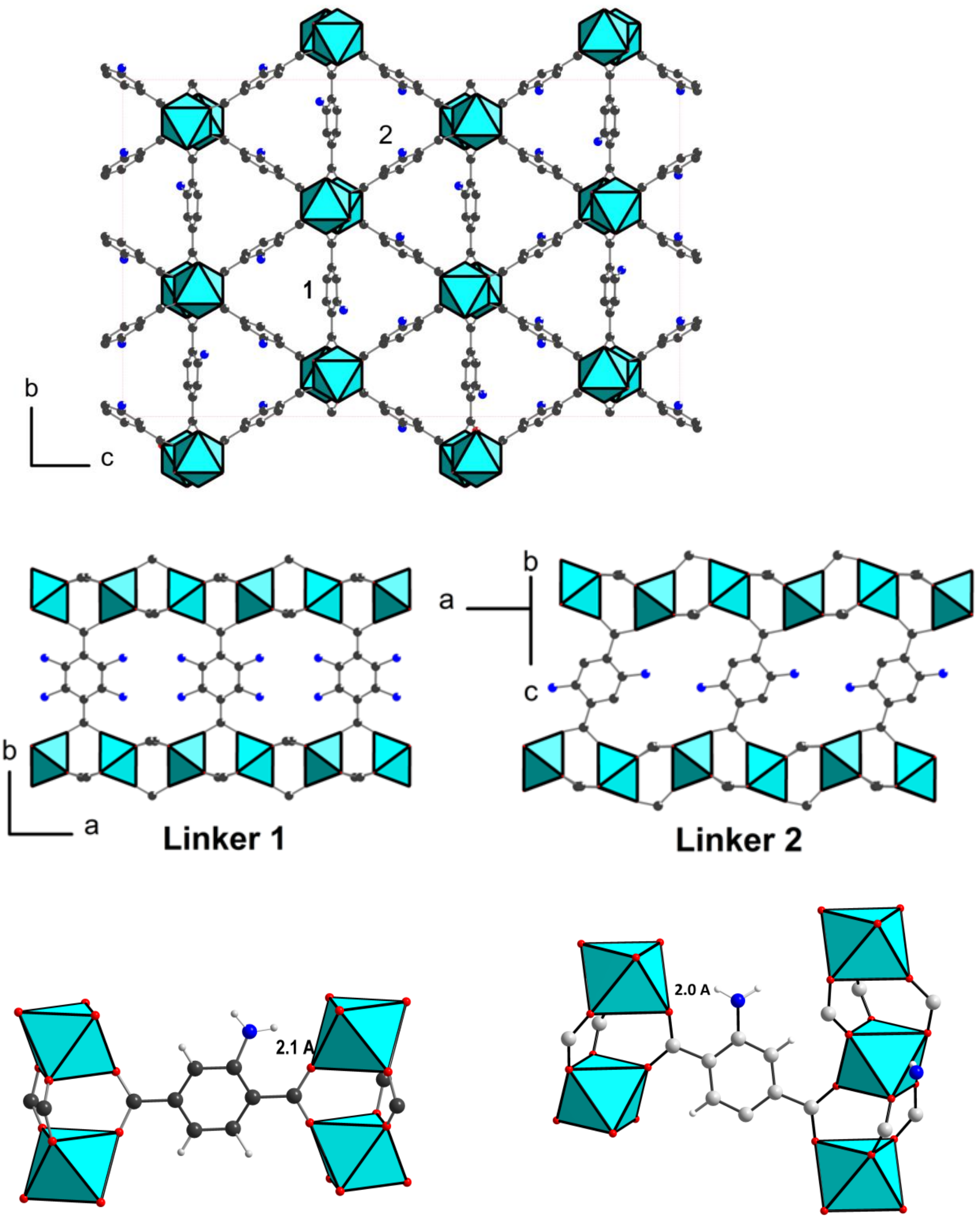

Figure S2.1 (Above) Framework structure of $Sc_2(BDC\text{-}NH_2)_3$ viewed down the channel axis. One possible arrangement of statistically disordered amino N atoms is shown. (Middle) Arrangement of linkers 1 and 2, showing all symmetrically equivalent positions of N atoms. ((C atoms, grey; N atoms, blue; H atoms omitted for clarity.) Below, left, One possible arrangement of linker 1, and right, of linker 2, with H atom positions and (N)H-O distances given.

Face Indexing

Face indexing of crystals of crystals of as prepared $Sc_2(BDC\text{-}NH_2)_3$ was used to establish the orientation of the crystallographic unit cell relative to the physical morphology of the crystal. Face indexing shows the longest (in microns) morphological direction of the crystals is parallel to the *a* axis of the crystallographic unit cell. Face indexing also reveals that the crystals have a regular morphology, with planes parallel to the long axis expressing the {001} and the {011} planes giving a pseudohexagonal cross section (Figures S2.2 and S2.3).

**Crystal system**	Orthorhombic	
**Space group**	*Fddd*	
**Unit cell dimensions**	a = 8.720(4) Å	α= 90°.
	b = 20.815(7) Å	β= 90°.
	c = 34.420(10) Å	γ = 90°.
**Volume**	6247(4) Å^3	
**Crystal size**	0.039 x 0.050 x 0.134 mm	

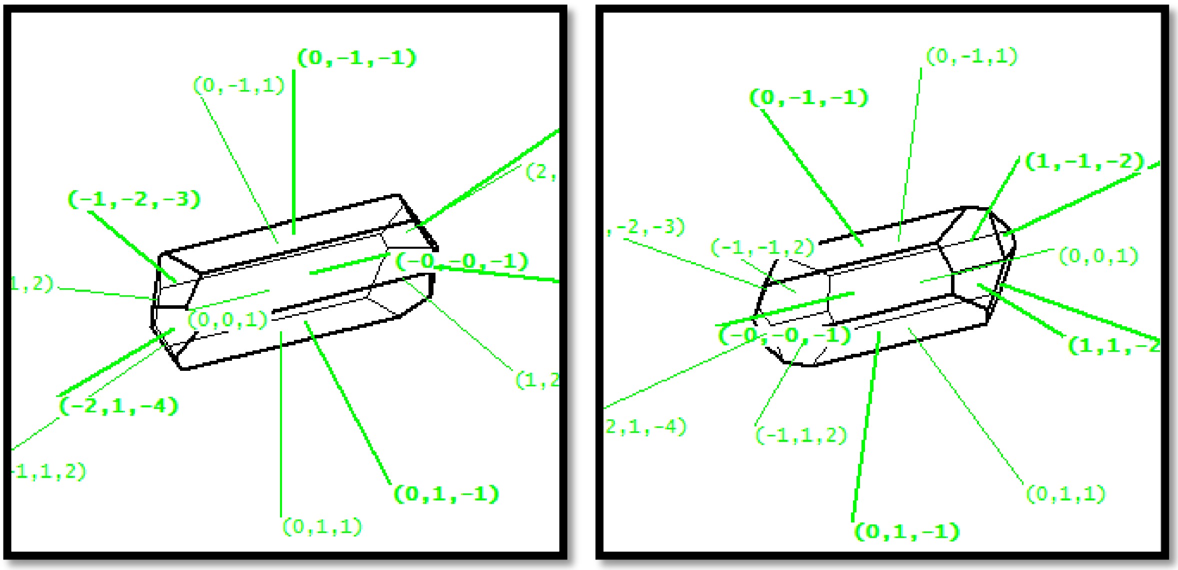

Figure S2.2 Crystal Shape (top) and crystal picture without face normals

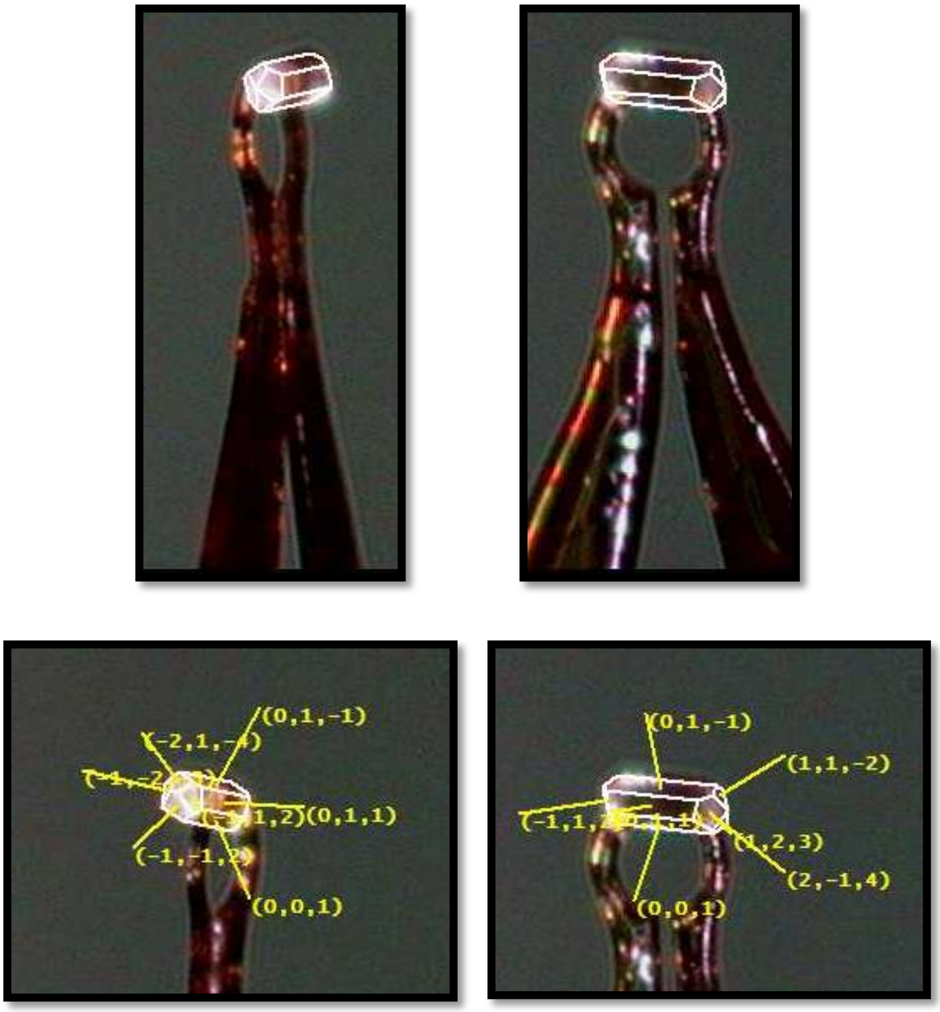

Figure S2.3 Crystal images with face normals. Note the main six faces along the longest axis are (0 0 1), (0 0 -1), (0 1 1), (0 -1 1), (0 1 -1) and (0 -1 -1)

S3 In situ gas adsorption variable temperature PXRD

A sample of $Sc_2(BDC\text{-}NH_2)_3$ was ground and packed into a 0.7 mm quartz glass capillary and held in place by a quartz glass plug The capillary was attached to a goniometer head that could be rocked by +/- 40° to improve powder averaging on the I11 beamline at Diamond Light Source (UK synchrotron). The sample was evacuated and heated to 400 K for 30 minutes. The activated sample was then allowed to attain 298 K over approximately 20 minutes. The sample was dosed to 100 mbar of CO_2 and allowed to equilibrate. A variable temperature experiment was conducted by using the cryostat probe available at I11 to control the temperature. The temperature was raised from 298 K to 400 K at 1 K per minute and diffraction patterns were collected at 10 K steps throughout the experiment (300 - 400 K) The PXRD pattern was measured using monochromated X-rays of wavelength 0.826956 Å using the Mythen position sensitive detector on the I11 beamline: 10 second scans were used to collect the diffraction patterns. Analysis of the diffraction patterns collected shows that the structure of $Sc_2(BDC\text{-}NH_2)_3$ is orthorhombic *Fddd* as shown by single crystal diffraction and does not undergo any phase changes or breathing effects over the range of temperatures tested.

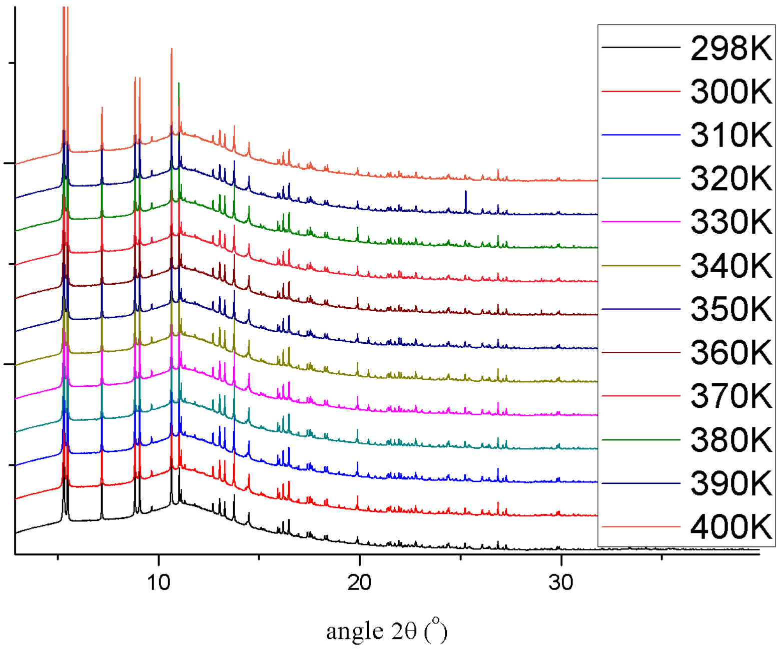

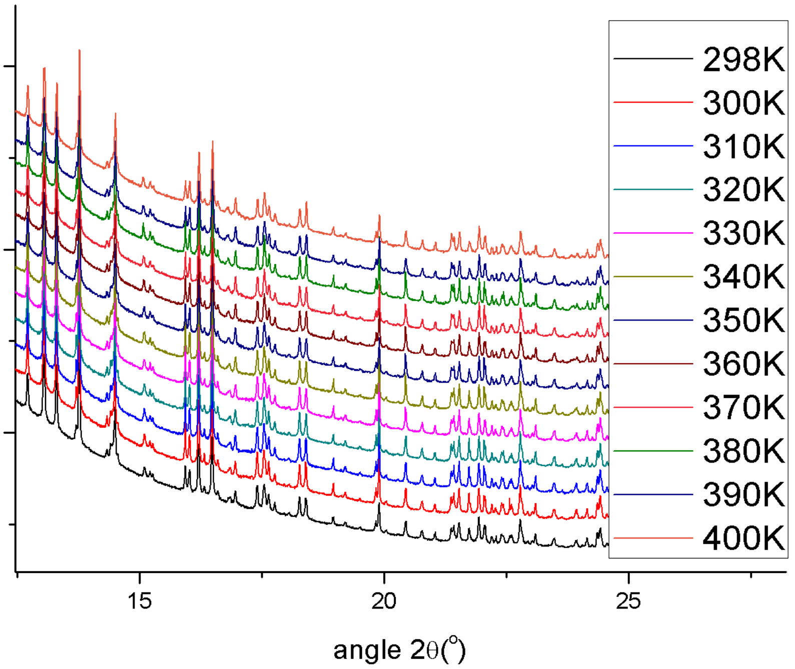

Figure S3.1 In situ synchrotron PXRD (λ = 0.826956 Å) of $Sc_2(BDC\text{-}NH_2)_3$ in contact with 0.1 bar CO_2.

S4. CO_2 Zero length column (ZLC) experiments

S4a Technique

The ZLC method is a chromatographic technique which allows the study of diffusion processes[3,4] and determination of the capacity of the adsorbed phase in porous systems.[5-7] The technique follows the desorption curve of the adsorbate from a small amount of sample, previously equilibrated with a gas mixture containing a low concentration of adsorbate in an inert carrier gas. The diffusion time constant is derived from the long time asymptote in the semi-log plot (C/C_0 vs t), whereas the capacity is found from the area under the desorption curve.

The ZLC method reveals that the desorption process of CO_2 from the $Sc_2(BDC\text{-}NH_2)_3$ is in the order of seconds. Moreover, it was used to measure the CO_2 capacity of the sample saturated with a mixture of 10% CO_2 in He, to validate the result gained from the volumetric gas sorption experiment at 30°C for CO_2 adsorption up to 0.1 bar.

S4b Experimental procedure

An amount of 13.3 mg of $Sc_2(BDC\text{-}NH_2)_3$ (without pre-drying) was loaded in the ZLC and the sample was regenerated overnight at 120 °C under the flow of He. It was then equilibrated at 30°C for 10 min with a mixture of 10% CO_2 in He and purged with pure He. The concentration of CO_2 was monitored using a quadrupole Mass Spectrometer (Ametek Benchtop). Both gases pass through drying columns before entering the system to remove any H_2O they may contain. The adsorption and purging gas flow rates (F) are set using mass flow controllers operating at 0 - 50 ml min^{-1}.

S4c Results

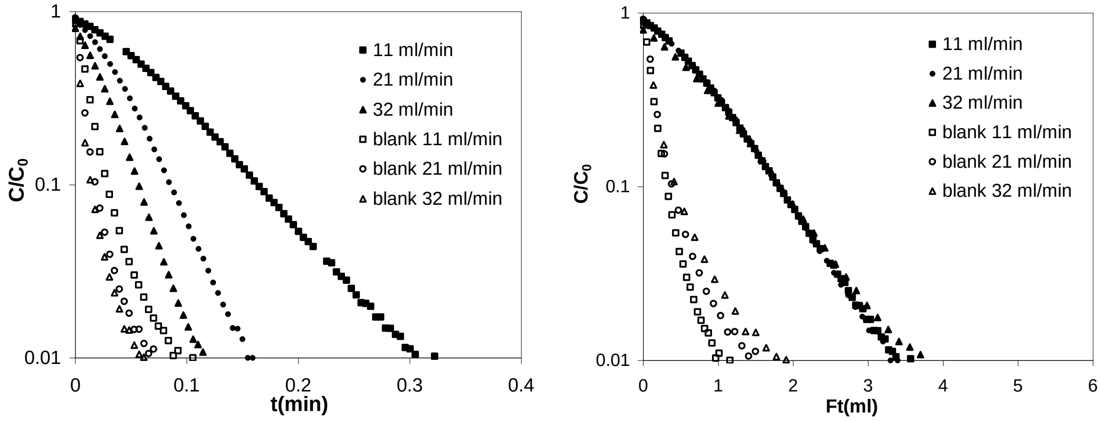

Figure S4.1 Desorption curves of CO_2 from $Sc_2(BDC\text{-}NH_2)_3$ and an empty ZLC column at different flowrates. The normalized decrease in concentration (C/C_0) is plotted against a) time (t) and b) Ft scales.

The t-plot (Fig S4-1a) shows the CO_2 desorption curves for the sample at flowrates between 11 and 32 ml min^{-1}. In the Ft-plot (Fig S4-1b) these curves overlap indicating that desorption is under equilibrium conditions, therefore the diffusivity is too fast to measure even at the highest flowrate considered of 32 ml/min. The CO_2 capacity of $Sc_2(BDC\text{-}NH_2)_3$ was calculated using the mass balance of the column[6,7] and was found to be ~0.22 mmol/g.

While it is not possible to determine the diffusional time constant from these experiments, it is possible to establish an upper bound (lower bound for the diffusivity). Assuming equilibrium control and the dimensionless parameter $L \approx 0.5$[8] from the slope of the long-time asymptote a maximum value of $R^2/D = 5.3$ s can be obtained.

S5 Single crystal micro FTIR: General Notes

S5a Data Collection

A small quantity (approximately 2 mg) of $Sc_2(BDC\text{-}NH_2)_3$ was evenly loaded over the sample stage of the Linkam cell, the cell was then sealed and mounted on to the microscope. Activation of samples was achieved by heating to 400K under flowing nitrogen for around 30 minutes before being cooled to 298 K over 20 minutes. For experiments in which the temperature was changed the sample was allowed to equilibrate for approximately 5 minutes once the desired temperature had been reached. From the ZLC experiments this equilibration period was shown to be well in excess of the time scale of adsorption / desorption process s samples of $Sc_2(BDC\text{-}NH_2)_3$.

Several different sites were selected from crystals on the sample stage; these sites were selected by optical microscopy (Fig. S5.1). On physically large crystals multiple sites were often selected. The aperture size of the microscope was set to probe a spot of approximately 10 × 10 µm. After each set of spectra were collected the optical image was inspected to ensure that crystals had not shifted from the site of interest, due to thermal or vibration induced movement, sites were re-selected and spectra were collected at the next set of relevant conditions.

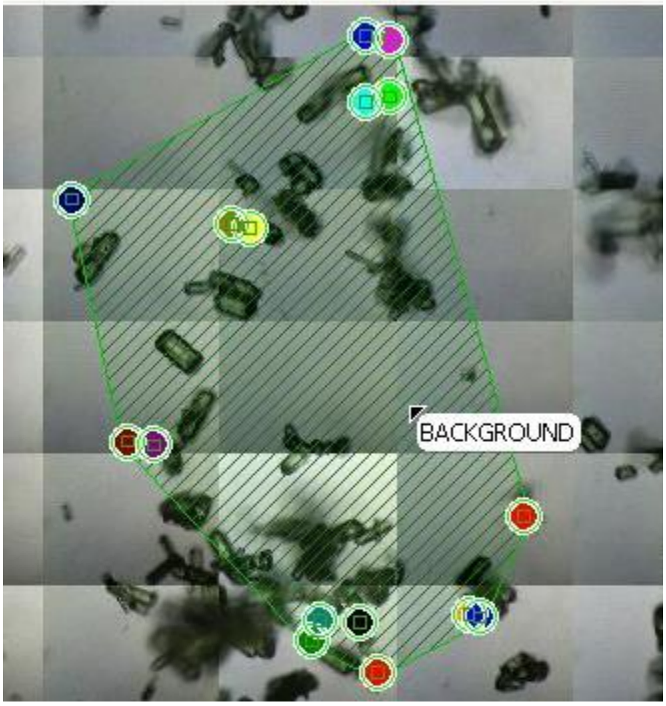

Figure S5.1 Several sites were selected from crystals on the sample stage; sites selected by visible light under the IR microscope.

Analysis of spectra

Spectra were initially checked visually to ensure good signal to noise ratio, data was omitted from spectra which did not meet these criteria. The usual explanation for such spectra was either due to sample drift during an experiment or falling close to the working limits of the spectrometer. A common feature of single crystal IR spectra is a sinusoidal baseline caused by internal interference fringes (See Fig S5.2). Spectra which exhibited severe baselines that interfered with features of the spectra were omitted. Spectra which contained limited oscillations were baseline corrected using linear functions in OPUS 7.2 (Fig. S5.3).

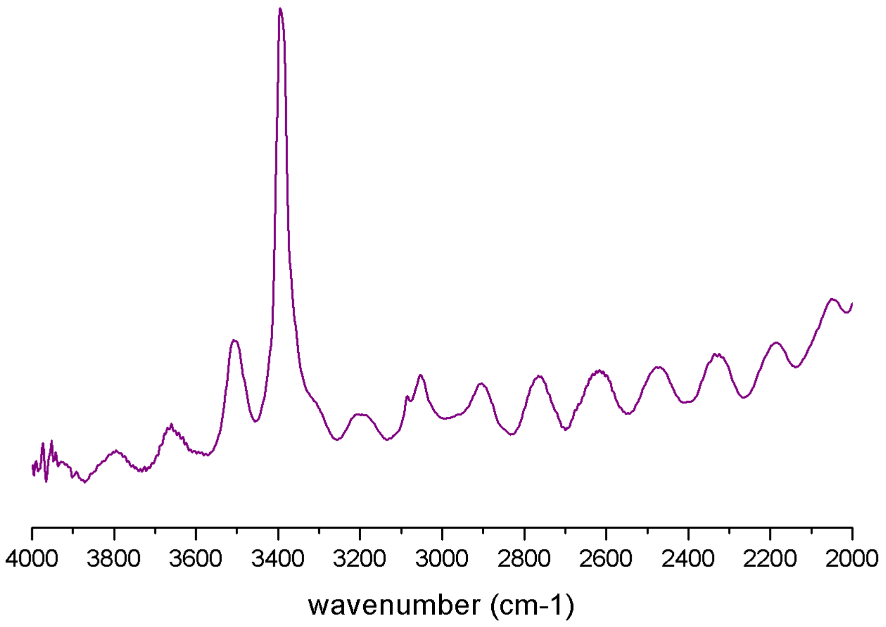

Figure S5.2 IR spectra with sinusoidal baseline caused by internal interference fringes.

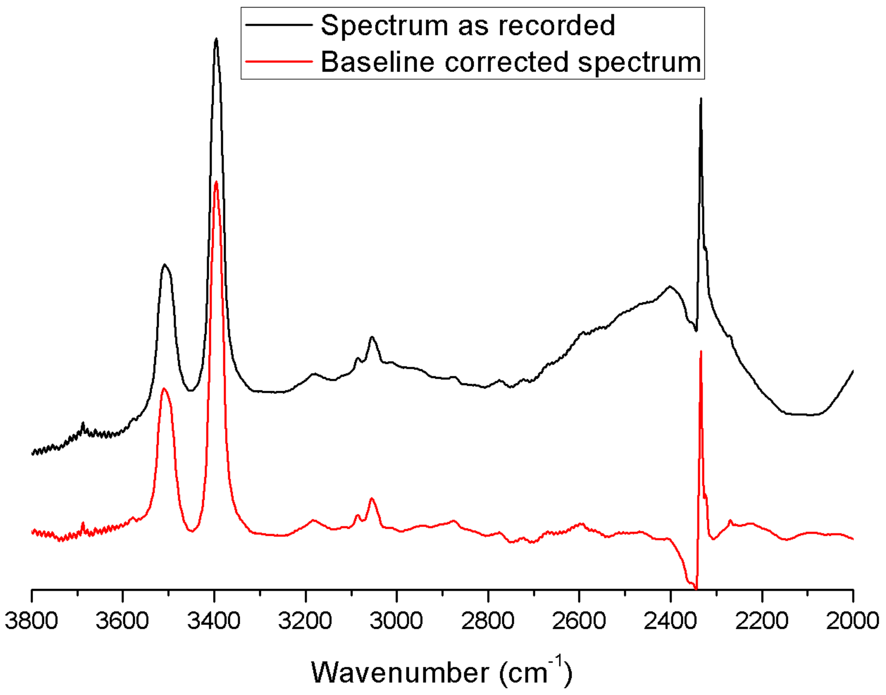

Figure S5.3 IR spectra with baseline corrected using linear functions in OPUS 7.2.

Integration of peaks was achieved using integration mode B in OPUS 7.2 in which a straight line is drawn between the two frequency limits of the defined peaks. The area above this line is then integrated (fig S5.4). For the peak due to the CO_2 asymmetric stretching mode spectra often showed a shallow minimum on the high frequency side at about 2345-2355 cm^{-1}. This due to incomplete compensation of the intense gas phase band at 2349 cm^{-1} between the background spectrum measured off the crystal and spectra measured on the crystal. The method used to integrate the CO_2 asymmetric peak was to visualise an asymptote of the baseline extended across the CO_2 minima / maxima peak feature and then define the limits of the integration at the point where the spectra crosses the imagined baseline (Fig S5.4). Thus, only the positive area on the right hand side of the band is used.

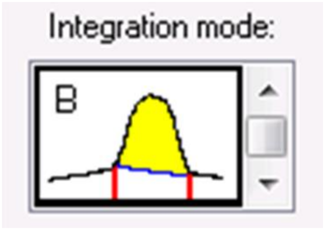

Schematic representation of integration mode B.

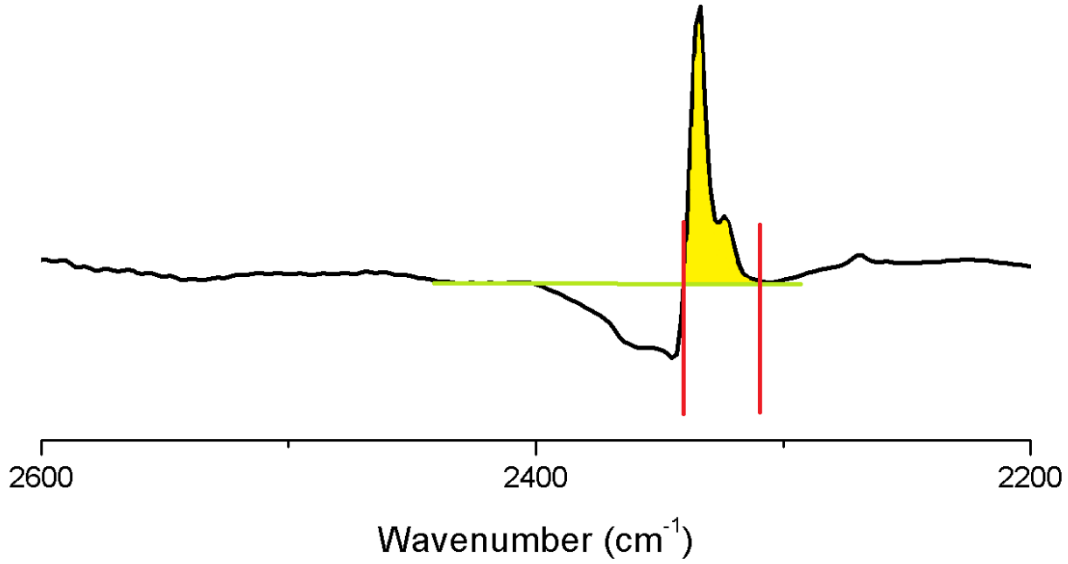

Figure S5.4 Schematic representation of method used to integrate CO_2 asymmetric stretch.

S6. Variable Temperature In situ-gas adsorption single crystal micro FTIR analysis

S6a Procedure

A sample of $Sc_2(BDC\text{-}NH_2)_3$ was loaded on to a CaF_2 window and enclosed within a Linkam FTIR600 cell (temperature and gas flow control). The sample was then loaded on to the sample stage of the Bruker Vertex 80 V Fourier Transform IR Interferometer fitted with a Hyperion 3000 microscope on beamline B22 at Diamond light source. The sample was heated to 400 K under flowing nitrogen for 30 minutes. The activated sample was then allowed to attain 298 K over approximately 20 minutes. The sample was dosed to approximately 100 mbar of CO_2 (1 bar of 10% CO_2 in N_2) and allowed to equilibrate for approximately 10 minutes. A variable temperature experiment was conducted by using the Linkam cell temperature control. The temperature was raised from 298K to 393K by pre-determined steps. Once the sample had attained the desired temperature it was allowed to equilibrate for approximately 5 minutes before non-polarised IR spectra were collected from various sites on a single crystal. Spectra were collected at the following temperatures: 298, 308, 323, 338, 353, 373 and 393 K.

S6b Results

Spectra were collected from 5 positions on the same crystal (Fig S6.1). Integrals were recorded for both the combined magnitude of the NH_2 stretches (approximately 3450-3350 cm^{-1}) and the CO_2 asymmetric stretch (approximately 2350-2315 cm^{-1}) using the previously highlighted method. A ratio for the CO_2: NH_2 integrals were then calculated.

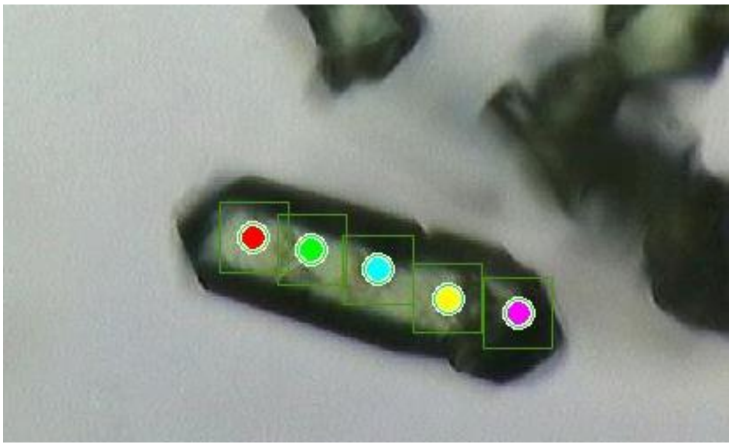

Figure S6.1 Multiple sites selected on a single crystal; Crystal site 1 (red circle) , crystal site 2 (green circle), crystal site 3 (blue circle), crystal site 4 (yellow circle) , crystal site 5 (pink circle).

Crystal site 1

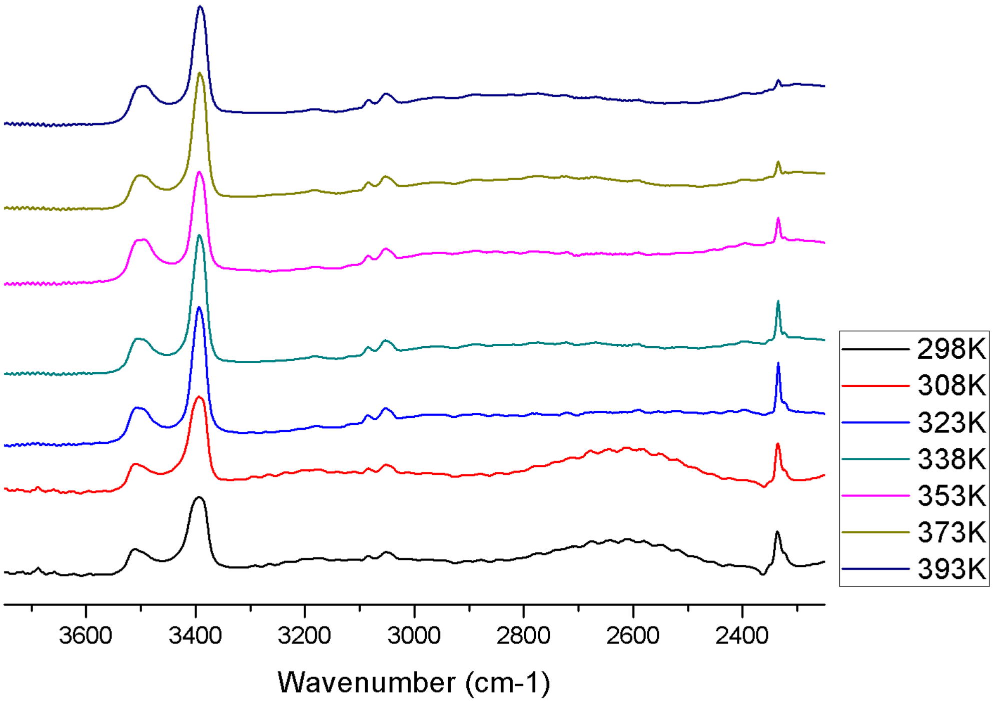

Temperature (K)	NH_2 Integral	CO_2 Integral	CO_2 :NH_2 Ratio
298	81.739	12.048	0.147
308	57.989	5.983	0.103
323	74.173	5.442	0.0734
338	76.087	3.525	0.046
353	71.278	2.389	0.034
373	74.185	1.182	0.016
393	74.287	0.794	0.011

Crystal site 2

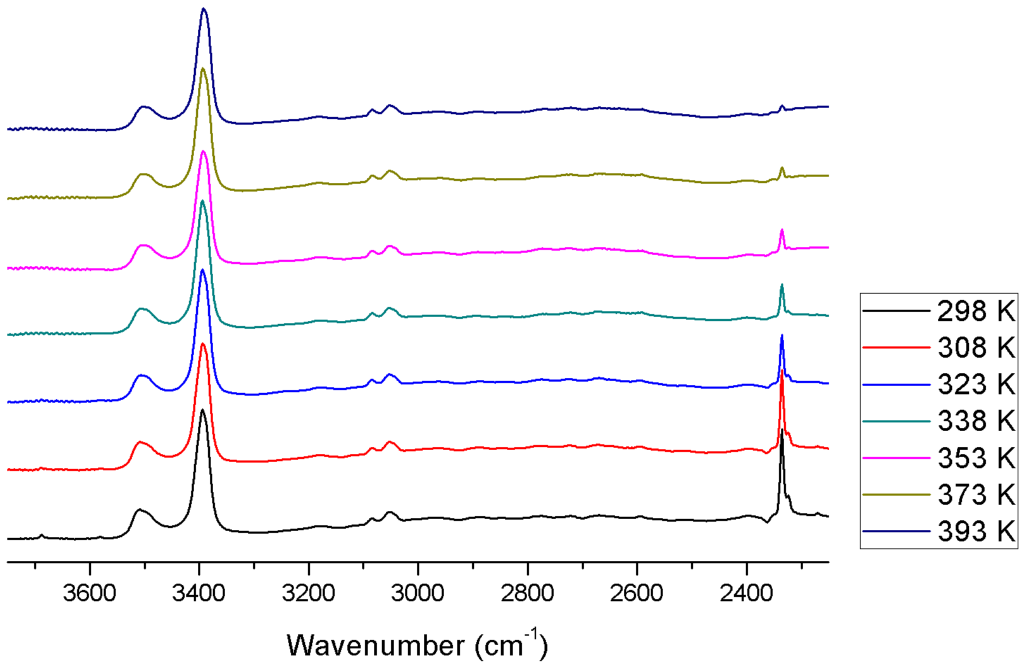

Temperature (K)	NH_2 Integral	CO_2 Integral	CO_2 :NH_2 Ratio
298	81.37	11.656	0.143
308	77.63	10.41	0.134
323	79.17	5.276	0.067
338	78.36	3.675	0.047
353	75.92	2.389	0.031
373	76.75	1.175	0.015
393	74.63	0.627	0.008

Crystal site 3

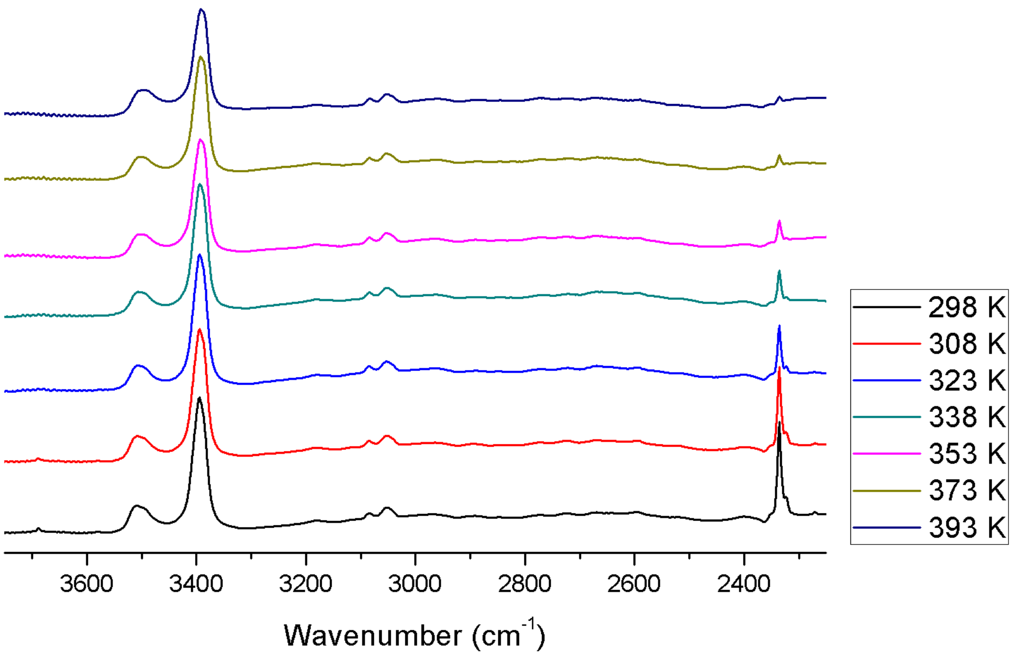

Temperature (K)	NH_2 Integral	CO_2 Integral	CO_2 :NH_2 Ratio
298	82.546	12.287	0.149
308	81.094	9.242	0.114
323	80.067	5.547	0.069
338	78.82	3.386	0.043
353	75.16	2.241	0.030
373	74.071	1.117	0.015
393	73.623	0.685	0.009

Crystal site 4

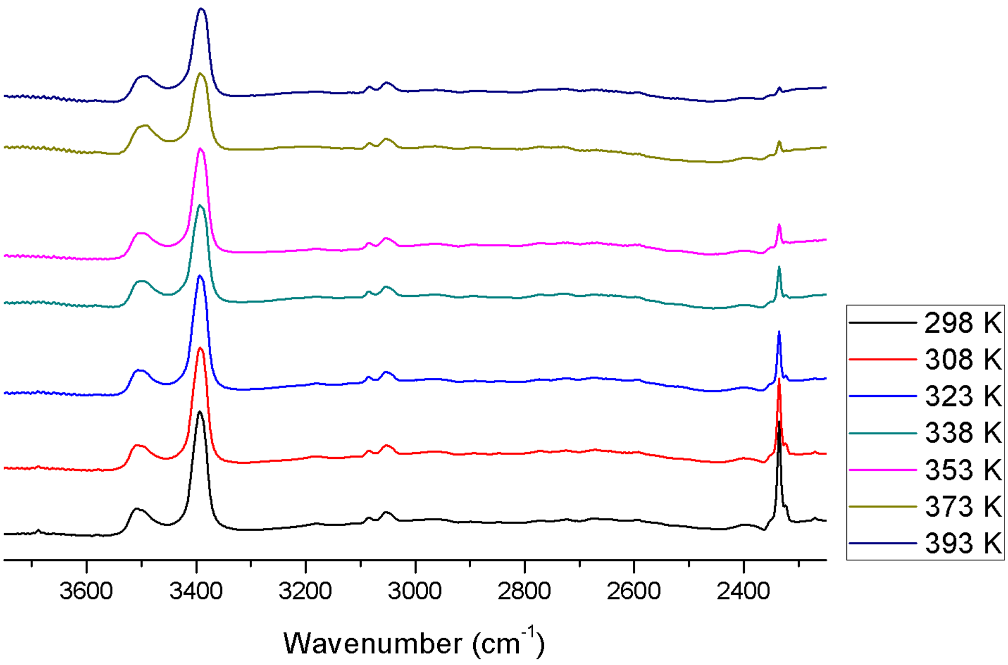

Temperature (K)	NH_2 Integral	CO_2 Integral	CO_2 :NH_2 Ratio
298	81.615	11.555	0.142
308	76.806	9.164	0.119
323	76.358	5.682	0.074
338	73.249	3.613	0.049
353	73.954	2.267	0.031
373	65.277	1.301	0.020
393	71.89	0.631	0.009

Crystal site 5

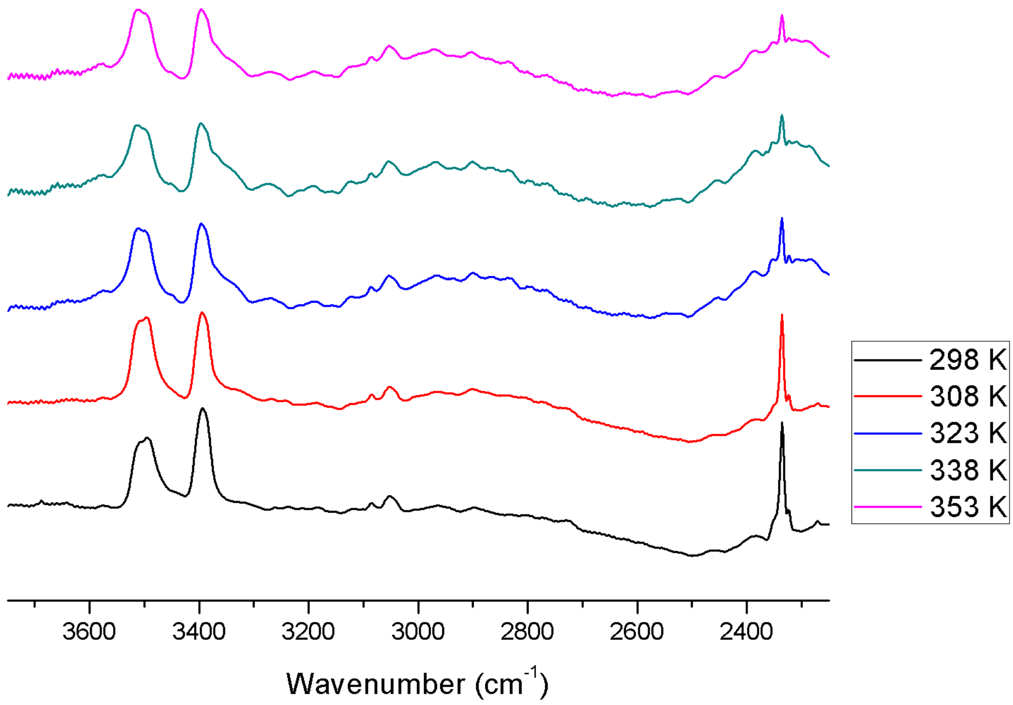

Temperature (K)	NH_2 Integral	CO_2 Integral	CO_2 :NH_2 Ratio
298	81.312	11.584	0.142
308	63.82	7.561	0.118
323	67.232	3.859	0.057
338	70.288	1.55	0.022
353	56.057	1.683	0.030

S6c Analysis of spectra

Collated data for each temperature collected:-

Temperature (K)	298			308		
Crystal site	NH_2 Integral	CO_2 Integral	CO_2 :NH_2 Ratio	NH_2 Integral	CO_2 Integral	CO_2 :NH_2 Ratio
1	81.739	12.048	0.147396	57.989	5.983	0.103175
2	81.37	11.656	0.143247	77.631	10.41	0.134096
3	82.546	12.287	0.14885	81.094	9.242	0.113967
4	81.615	11.555	0.141579	76.806	9.164	0.119314
5	81.312	11.584	0.142464	63.82	7.561	0.118474

Temperature (K)	323			338		
Crystal site	NH_2 Integral	CO_2 Integral	CO_2 :NH_2 Ratio	NH_2 Integral	CO_2 Integral	CO_2 :NH_2 Ratio
1	74.173	5.442	0.073369	76.087	3.525	0.046329
2	79.174	5.276	0.066638	78.364	3.675	0.046897
3	80.067	5.547	0.069279	78.82	3.386	0.042959
4	76.358	5.682	0.074413	73.249	3.613	0.049325
5	67.232	3.859	0.057398	70.288	1.55	0.022052

Temperature (K)	353			373		
Crystal site	NH_2 Integral	CO_2 Integral	CO_2 :NH_2 Ratio	NH_2 Integral	CO_2 Integral	CO_2 :NH_2 Ratio
1	71.278	2.389	0.033517	74.185	1.182	0.015933
2	75.919	2.389	0.031468	76.753	1.175	0.015309
3	75.16	2.241	0.029816	74.071	1.117	0.01508
4	73.954	2.267	0.030654	65.277	1.301	0.01993
5	56.057	1.683	0.030023			

Temperature (K)	393		
Crystal site	NH_2 Integral	CO_2 Integral	CO_2 :NH_2 Ratio
1	74.287	0.794	0.010688
2	74.63	0.627	0.008401
3	73.623	0.685	0.009304
4	71.89	0.631	0.008777

Taking the average CO_2: NH_2 ratio for the data collected at 298K at giving it the value equal to the uptake interpolated at 100 mbar of CO_2 in the gravimetric isotherm at 298K of 0.3929 mmol g^{-1}, it is then possible to calculate predicted uptakes for the remaining values of the CO_2: NH_2 ratios.

Crystal SIte	Temperature (K)	CO_2: NH_2 ratio	Predicted Uptake (mmolg^{-1})
1	298	0.147396	0.400173
2	298	0.143247	0.388908
3	298	0.14885	0.404122
4	298	0.141579	0.384381
5	298	0.142464	0.386782
1	308	0.103175	0.280114
3	308	0.113967	0.309414
4	308	0.119314	0.323931
5	308	0.118474	0.321651
1	323	0.073369	0.199193
2	323	0.066638	0.180919
3	323	0.069279	0.18809
4	323	0.074413	0.202027
5	323	0.057398	0.155834
1	338	0.046329	0.12578
2	338	0.046897	0.127322
3	338	0.042959	0.116631
4	338	0.049325	0.133915
1	353	0.033517	0.090996
2	353	0.031468	0.085433
3	353	0.029816	0.08095
4	353	0.030654	0.083225
5	353	0.030023	0.081511
1	373	0.015933	0.043258
2	373	0.015309	0.041563
3	373	0.01508	0.040942
4	z373	0.01993	0.05411
1	393	0.010688	0.029018
2	393	0.008401	0.02281
3	393	0.009304	0.02526
4	393	0.008777	0.02383

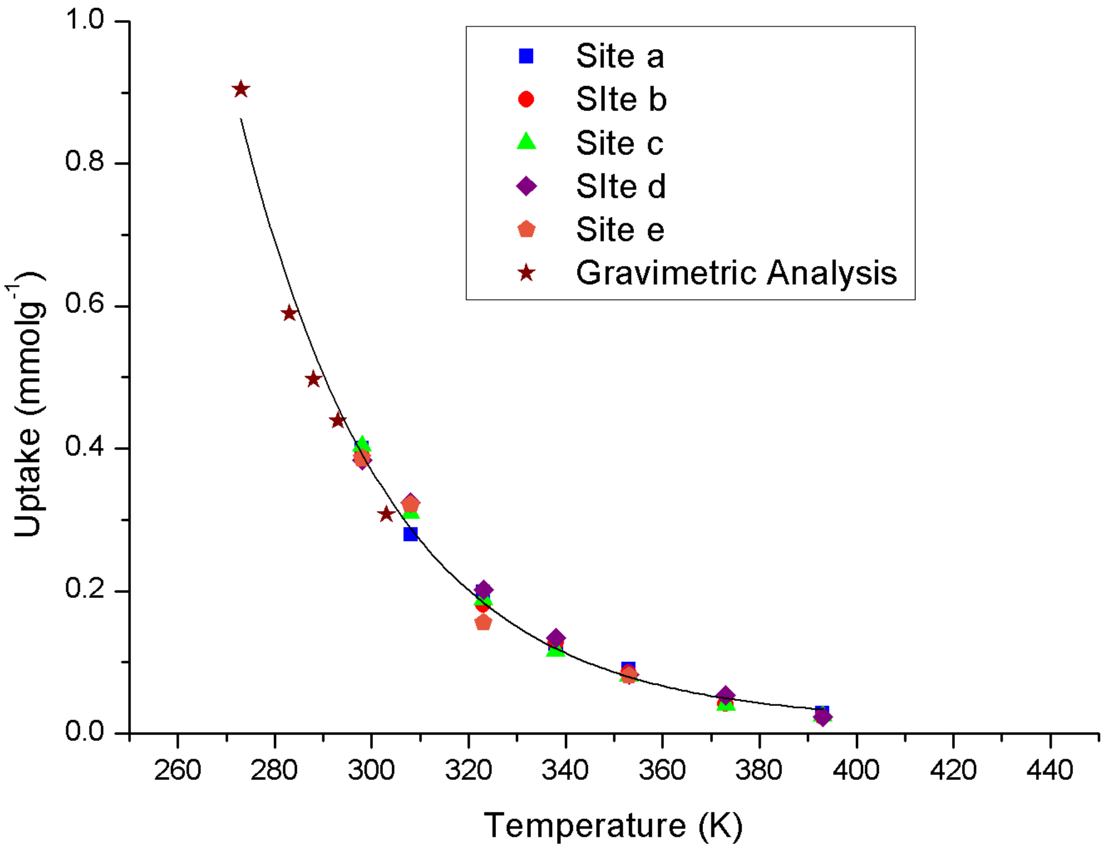

Figure S6.2 Plot of calculated uptake from IR intensities measured on different points on a single crystal against temperature, compared with uptakes measured gravimetrically.

The predicted uptake quantities at the various temperatures were then used to calculate coverage (assuming a total uptake of 5.1 mmol $g^{-1})^2$ and hence a value for the heat of adsorption for CO_2 in $Sc_2(BDC\text{-}NH_2)_3$ calculated from the gradient of a plot of lnK vs. 1/T (Fig. 6.3)

Temp, T (K)	1/T	Total uptake (mmol g^{-1})	Estimated uptake (mmol g^{-1})	Coverage θ	K θ / P_{CO2}(1-θ)	lnK
298	0.0034	5.1000	0.3929	0.0770	0.8347	-0.1807
308	0.0032	5.1000	0.3218	0.0631	0.6736	-0.3952
323	0.0031	5.1000	0.1858	0.0364	0.3782	-0.9724
338	0.0030	5.1000	0.1135	0.0222	0.2276	-1.4803
353	0.0028	5.1000	0.0845	0.0166	0.1685	-1.7808
373	0.0027	5.1000	0.0447	0.0088	0.0883	-2.4266
393	0.0025	5.1000	0.0252	0.0049	0.0497	-3.0011

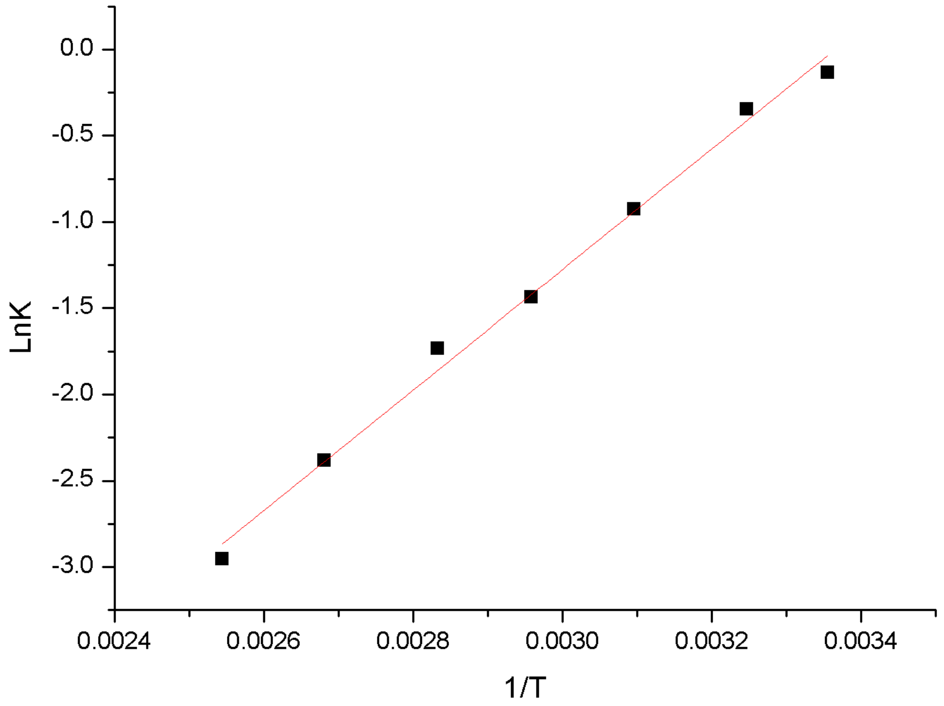

Figure S6.3 Plot of lnK vs. 1/T for adsorption of CO_2 on a single crystal of $Sc_2(BDC\text{-}NH_2)_3$

(y = 3490.2x - 11.79, σ(slope) = 121, ΔH = 29.0 ± 1.0 kJ mol^{-1})

S7 Variable Temperature In situ-gas adsorption Single crystal micro FTIR analysis, CO_2 isobars (25 – 200 mbar)

S7a Procedure

Having demonstrated the feasibility of the variable temperature *in situ* gas adsorption single crystal micro FTIR analysis technique for quantification of the adsorption process at 100 mbar partial pressure of CO_2 in a single crystal of Sc_2-(BDC-NH_2)$_3$, the technique was used to determine uptakes over isobars of CO_2 at 25, 50, 100 and 200 mbar partial pressure of CO_2 on multiple single crystals from the same batch of Sc_2(BDC-NH_2)$_3$. The procedure followed was the same as previously described applied sequentially to the series of isobars.

S7b Results

Sample sites used for data collection and analysis:-

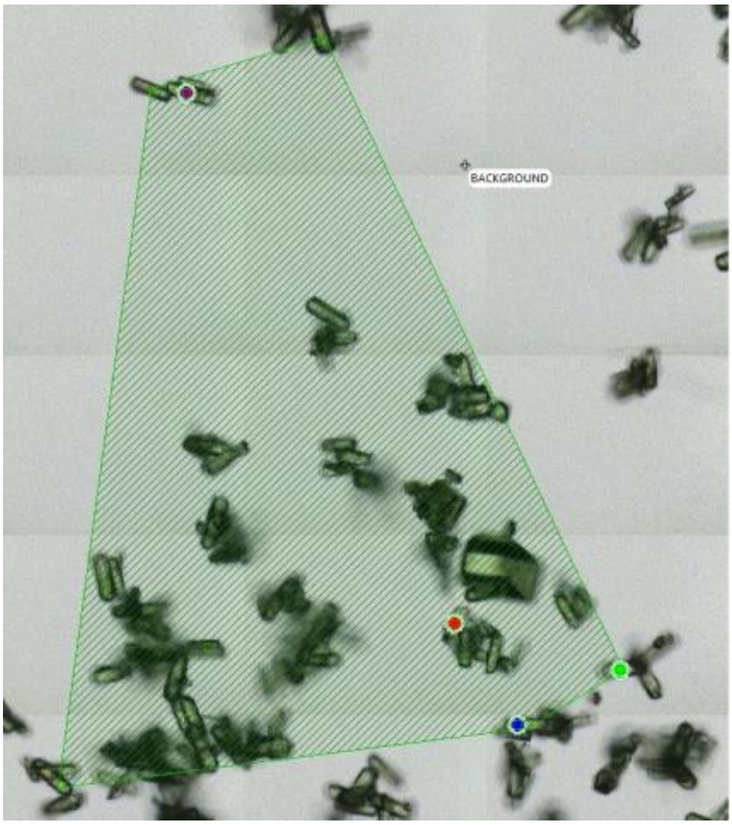

SITE 1 = Green circle

SITE 2= Blue Circle

SITE 3= Red Circle

SITE 4= Purple circle

Figure S7.1 Optical micrograph of single crystals of Sc_2(BDC-NH_2)$_3$ in the Linkam cell

Site 1, spectra:-

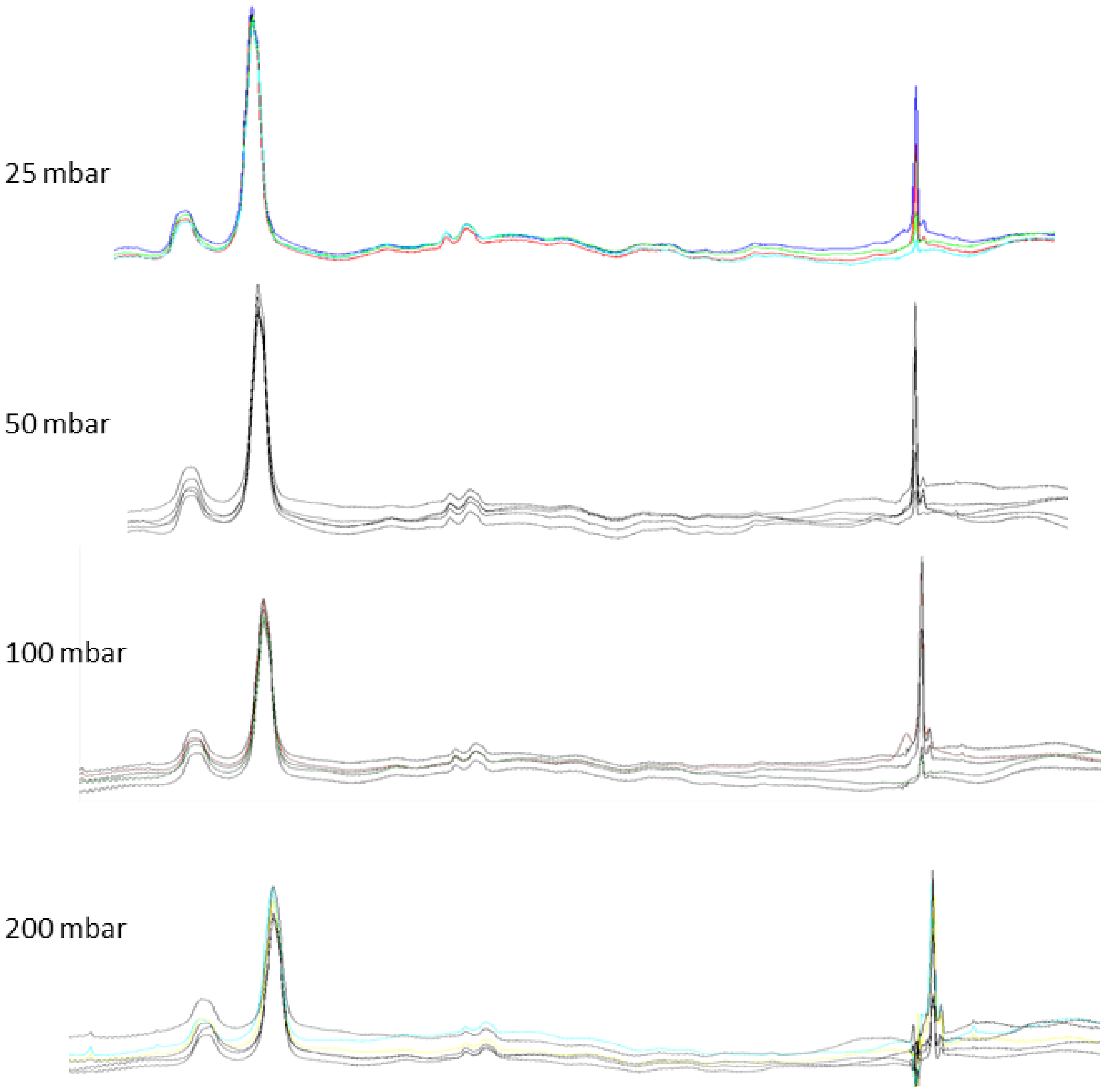

Site 2, spectra:-

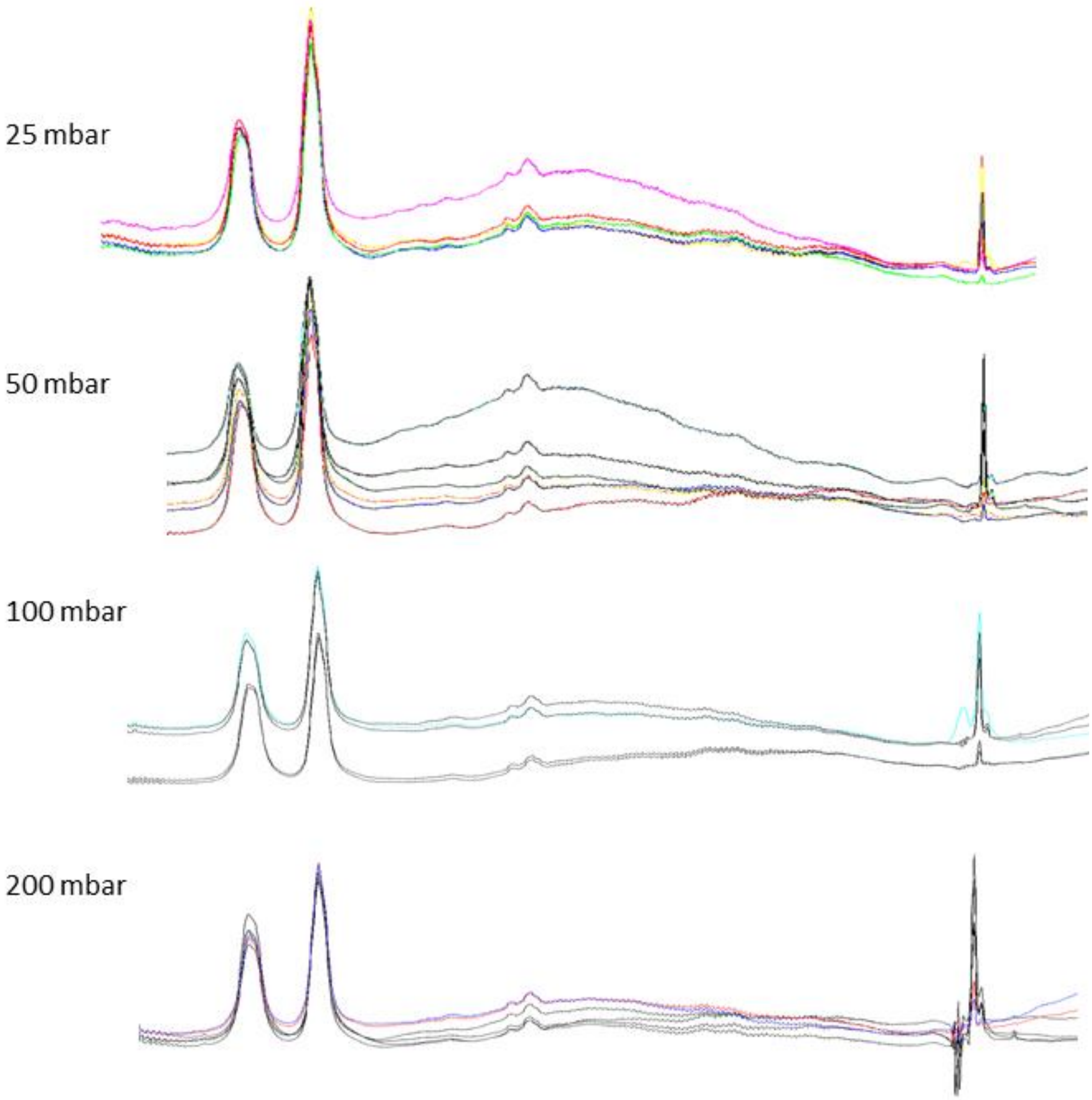

Site 3, spectra:-

25 mbar

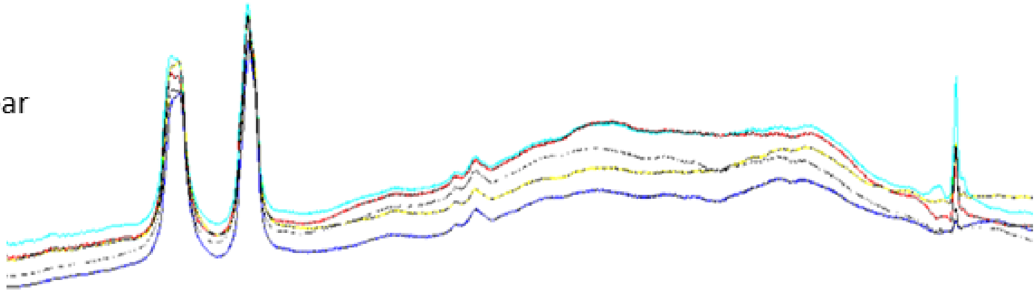

50 mbar

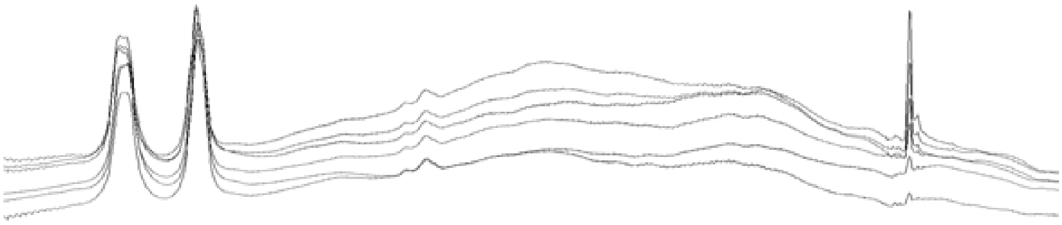

100 mbar

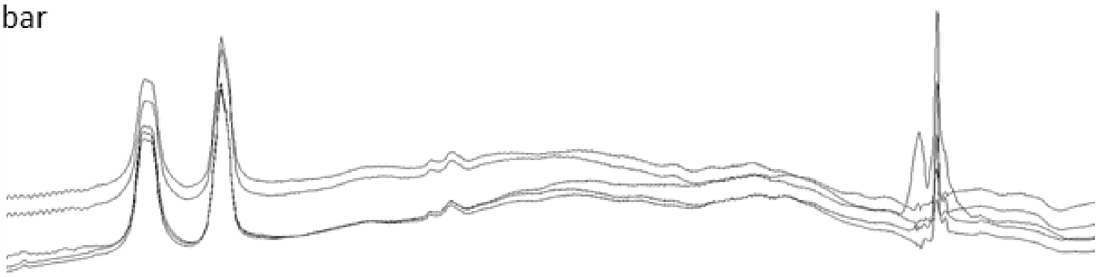

200 mbar

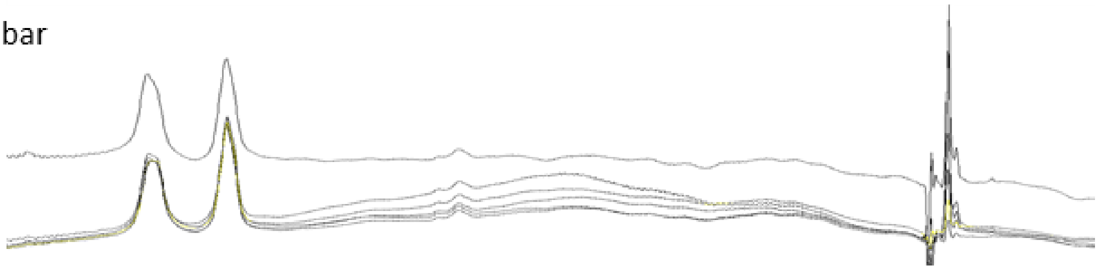

Site 4, spectra:-

25 mbar

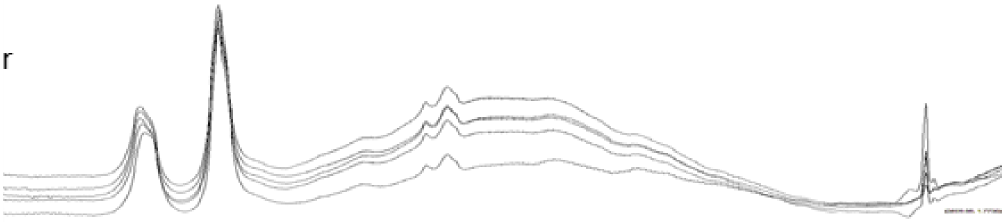

50 mbar

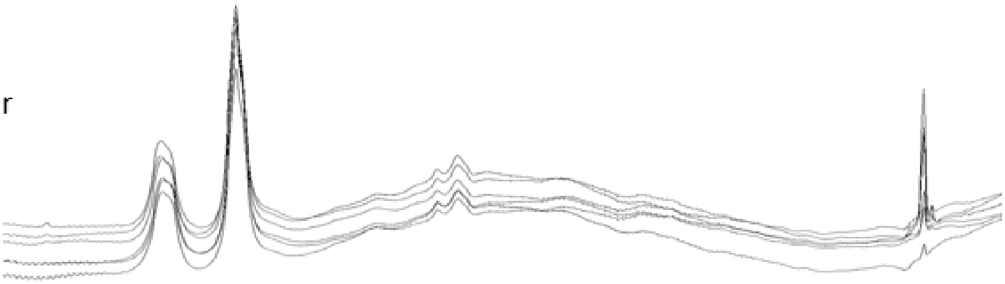

100 mbar

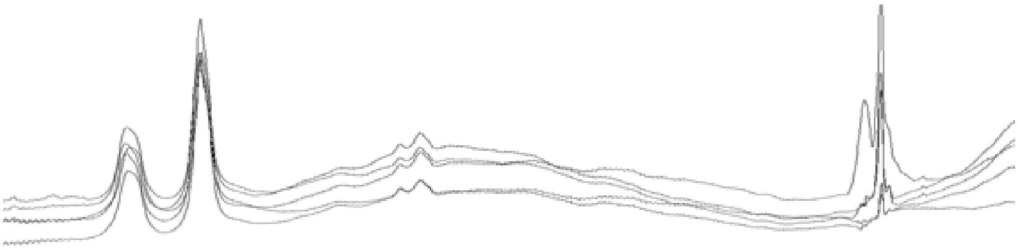

200 mbar

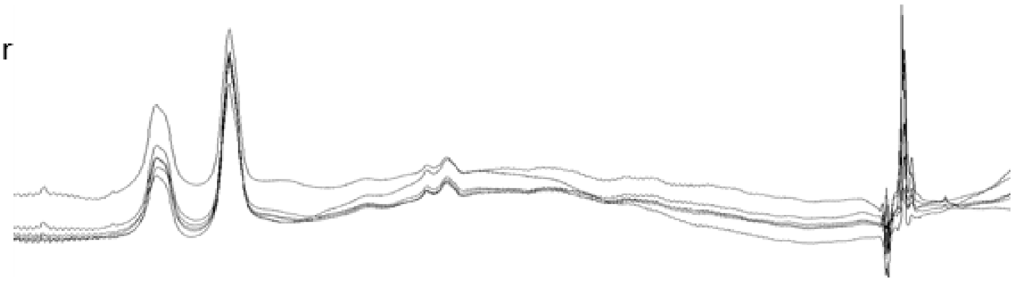

S7c Heat of adsorption charts

200 mbar partial pressure CO_2

Temp / T (K)	1/T (K^{-1})	Total Uptake (mmol g^{-1})	Estimated uptake (mmol g^{-1})	Fractional Coverage (θ)	K $\theta/P_{CO2}(1-\theta)$	ln K
298	0.00336	5.1	0.6227	0.1221	1.3908	0.3299
308	0.0032	5.1	0.4997	0.0980	1.0862	0.0827
323	0.0031	5.1	0.3890	0.0763	0.8258	-0.1914
338	0.003	5.1	0.2348	0.0460	0.4827	-0.7284
353	0.0028	5.1	0.1192	0.0234	0.2392	-1.4303
373	0.0027	5.1	0.0827	0.0162	0.1649	-1.8023

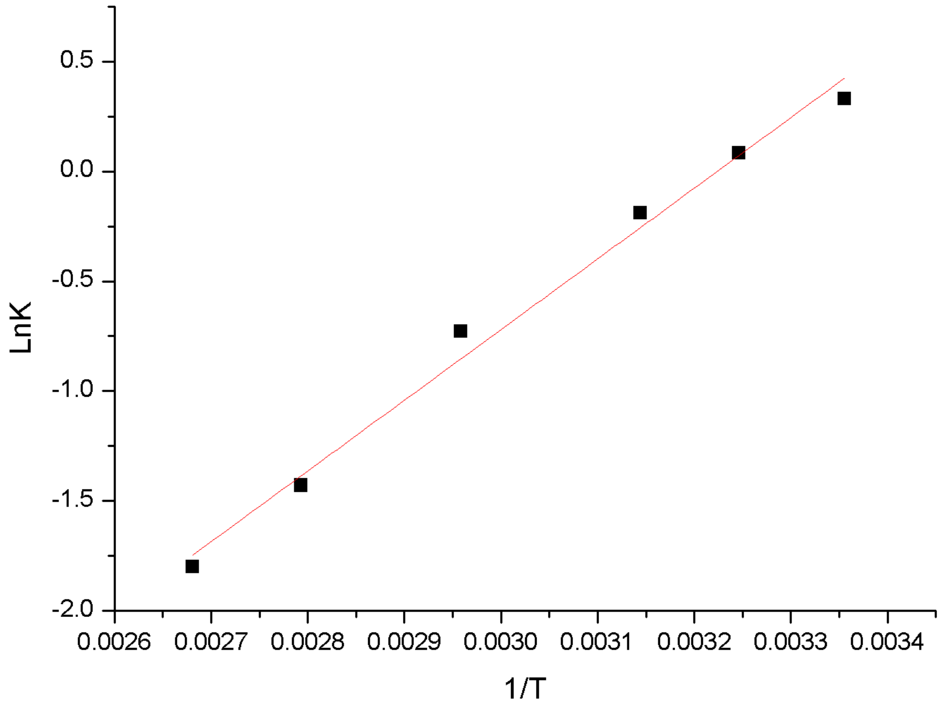

y = 3216.4x - 10.370, standard error in slope = 155.5

$\Delta H = 26.7 \pm 1.3\ kJmol^{-1}$

100 mbar partial pressure CO_2

Temperature (K)	1/T	Total Uptake (mmol g^{-1})	estimated Uptake (mmol g^{-1})	coverage	$K=\theta / P_{CO2}(1-\theta)$	lnK
298	0.0034	5.1	0.3939	0.0772	0.8370	-0.1779
308	0.0032	5.1	0.3002	0.0589	0.6254	-0.4693
323	0.0031	5.1	0.2240	0.0439	0.4594	-0.7779
338	0.003	5.1	0.1050	0.0206	0.2102	-1.5599
353	0.0028	5.1	0.0497	0.0097	0.0984	-2.3190
373	0.0027	5.1	0.0336	0.0066	0.0663	-2.7138

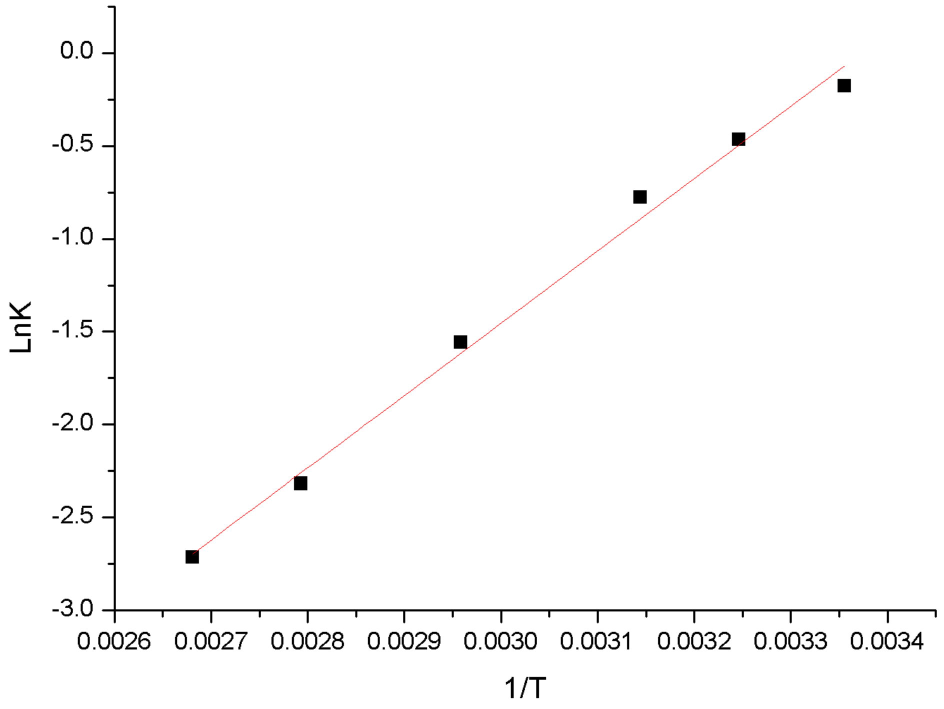

y = 3382.76x – 10.84, σ(slope) = 151.8

$\Delta H = 28.1 \pm 1.3$ kJ mol^{-1}

50 mbar partial pressure CO_2

Temperature (K)	1/T	Total Uptake (mmol g^{-1})	estimated uptake (mmol g^{-1})	coverage	$K=\theta / P_{CO2}(1-\theta)$	lnK
298	0.0034	5.1	0.2047	0.0401	0.4182	-0.8717
308	0.0032	5.1	0.1469	0.0288	0.2967	-1.2151
323	0.0031	5.1	0.0926	0.0182	0.1849	-1.6879
338	0.003	5.1	0.0466	0.0091	0.0923	-2.3831
353	0.0028	5.1	0.0215	0.0042	0.0424	-3.1604
373	0.0027	5.1	0.0131	0.0026	0.0257	-3.6619

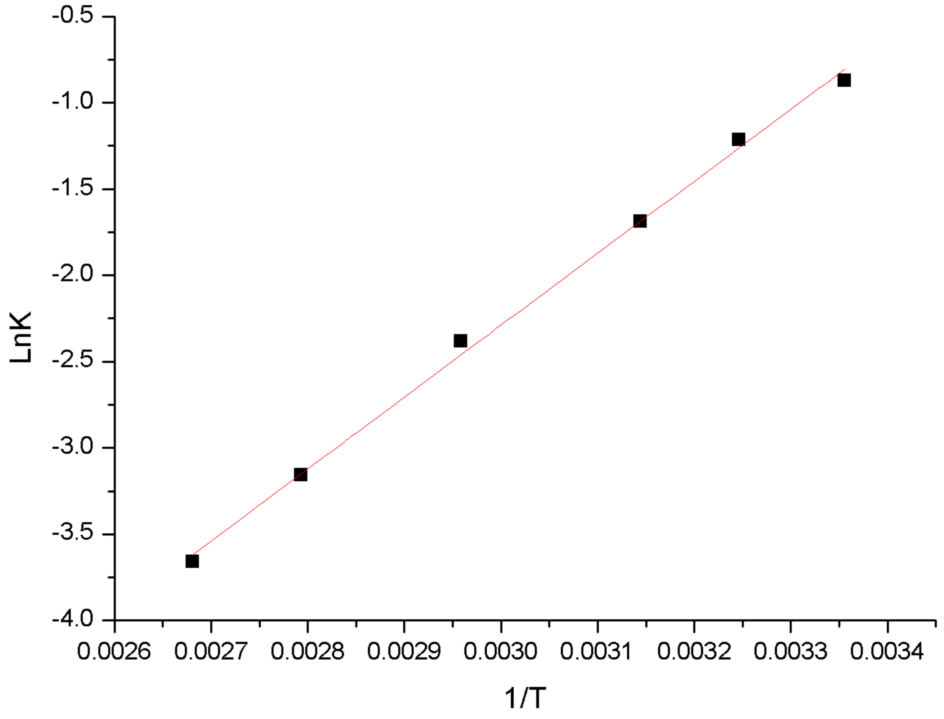

$y = 4168.4x - 14.79$, σ (slope) = 101.7

$\Delta H = 34.7 \pm 0.8$ kJ mol^{-1}

25 mbar partial pressure CO_2

Temperature (K)	1/T	Total Uptake (mmol g^{-1})	estimated Uptake (mmol g^{-1})	coverage	$K=\theta / P_{CO2}(1-\theta)$	lnK
298	0.0034	5.1	0.1082	0.0212	0.2168	-1.5288
308	0.0032	5.1	0.0658	0.0129	0.1308	-2.0344
323	0.0031	5.1	0.0412	0.0081	0.0815	-2.5077
338	0.003	5.1	0.0198	0.0039	0.0390	-3.2440
353	0.0028	5.1	0.0067	0.0013	0.0132	-4.3246

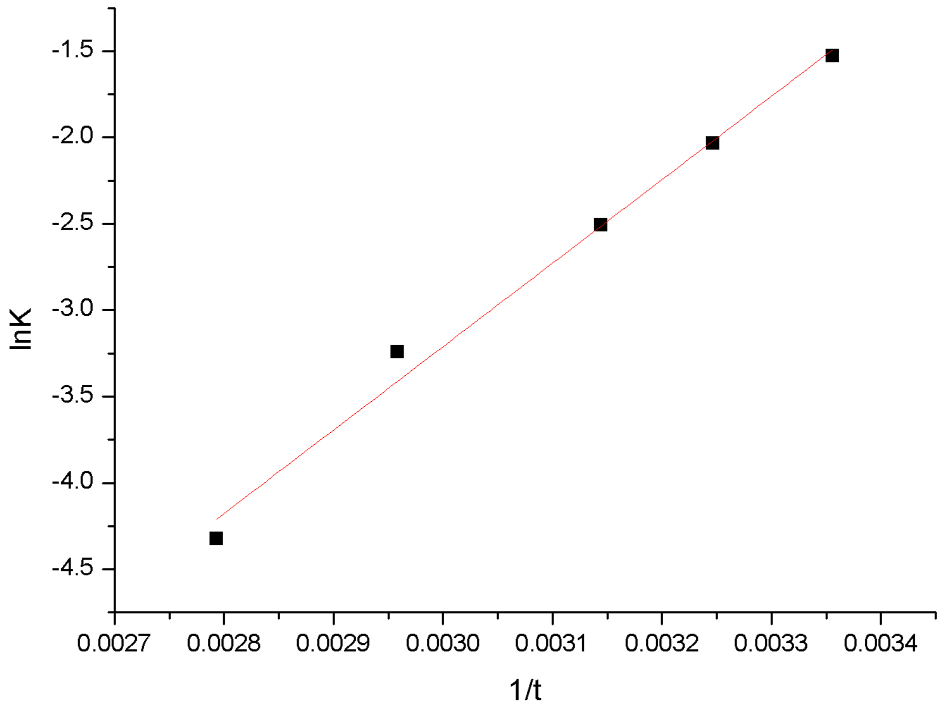

y = 4832.6x – 17.71, σ(slope) = 135

$\Delta H = 35.9 \pm 1.1$ kJ mol^{-1}

Overall, average $\Delta H = 31 \pm 2$ kJ mol^{-1}

S8 Gravimetric CO_2 adsorption isotherms and isosteric heats

Carbon dioxide isotherms for functionalised Sc2(BDC-NH2)3 were collected up to 900 mbar at a range of temperatures including 303, 293, 288, 283 and 273 K (using a Grant GR150 thermostatic refrigerated bath for temperature control) using a Hiden IGA automatic gravimetric porosimeter. Prior to adsorption of CO_2 the samples were heated at 400 K under a vacuum of 3 x 10^{-7} mbar for 12h. During this degassing period samples lost very little mass (< 2%). The adsorption at each pressure point was taken at a value predicted to be 98% of its increase in uptake towards equilibrium, up to a maximum wait time of 120 minutes.

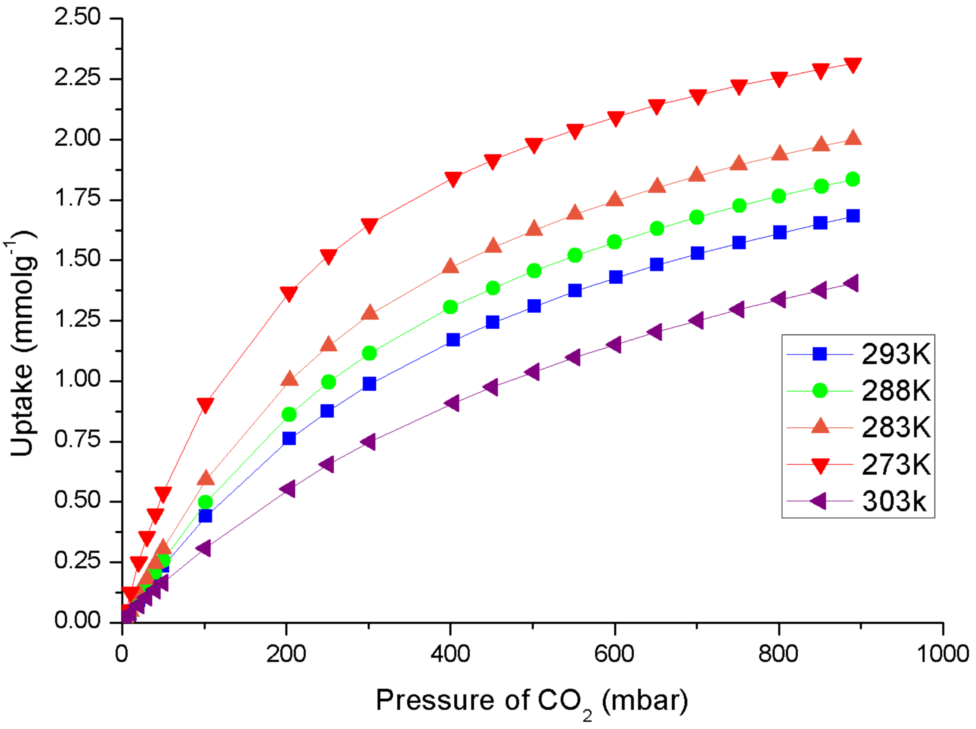

Figure S8.1 CO_2 adsorption isotherms on $Sc_2(BDC\text{-}NH_2)_3$, measured gravimetrically.

Temperature = 273 K

Pressure(mbar)	Uptake ($mmolg^{-1}$)
2.021	0.003
3.014	0.018
4.014	0.035
5.020	0.051
10.152	0.125
20.165	0.250
30.157	0.354
40.159	0.448
50.159	0.537
101.640	0.905
203.173	1.368
251.556	1.523
301.496	1.649
403.250	1.842
451.343	1.914
501.428	1.982
551.506	2.041
601.273	2.094
651.088	2.142
701.118	2.185
751.459	2.225
799.835	2.256
850.875	2.290
889.884	2.315

Temperature = 283 K

Pressure(mbar)	Uptake ($mmolg^{-1}$)
4.026	0.001
5.028	0.009
10.143	0.047
20.172	0.117
30.172	0.180
40.205	0.244
50.183	0.305
101.737	0.590
203.913	1.002
251.515	1.146
301.738	1.275
399.909	1.468
451.751	1.552
501.615	1.625
551.679	1.689
599.993	1.745
651.365	1.801
699.962	1.848
750.836	1.893
800.748	1.935
851.400	1.973
890.002	1.999

Temperature = 288 K

Pressure(mbar)	Uptake (mmolg-1)
10.157	0.034
20.192	0.098
30.219	0.154
40.198	0.205
50.178	0.258
101.772	0.497
203.533	0.861
251.598	0.996
301.621	1.115
400.034	1.305
451.807	1.384
501.905	1.456
551.658	1.520
600.097	1.575
651.628	1.630
700.108	1.678
751.971	1.725
800.153	1.765
851.767	1.807
890.016	1.835

Temperature = 293 K

Pressure(mbar)	Uptake (mmolg-1)
5.051	0.005
10.171	0.034
20.239	0.091
30.085	0.139
40.206	0.188
50.204	0.233
102.339	0.439
203.858	0.760
250.491	0.875
301.939	0.987
403.797	1.168
452.146	1.241
502.168	1.310
552.212	1.372
602.262	1.428
651.365	1.480
701.913	1.528
751.846	1.572
802.007	1.613
851.137	1.652
891.192	1.681

Temperature = 303 K

Pressure(mbar)	Uptake (mmolg-1)
0.005	0.001
0.012	0.002
0.010	0.004
0.022	0.009
0.033	0.009
0.042	0.009
0.052	0.008
0.062	0.008
0.072	0.008
0.081	0.008
0.092	0.008
0.101	0.008
0.200	0.007
0.300	0.006
0.404	0.006
0.500	0.005
1.002	0.007
2.010	0.010
3.000	0.014
4.011	0.017
5.014	0.021
10.146	0.039
20.172	0.073
30.153	0.105
40.138	0.135
50.189	0.164
101.592	0.308
203.769	0.554
251.190	0.654
301.704	0.748
403.631	0.909
451.364	0.974
501.442	1.038
551.624	1.097
601.605	1.152
651.199	1.203
701.429	1.251
750.850	1.296
801.558	1.338
851.131	1.377
890.486	1.407

Curves were fitted to the gravimetric data using polynomial functions and from these equations values of pressure could be calculated for fixed coverage (uptake). A van't Hoff plot of lnP vs 1 /T was used to calculate the isosteric heat of adsorption at various loadings.

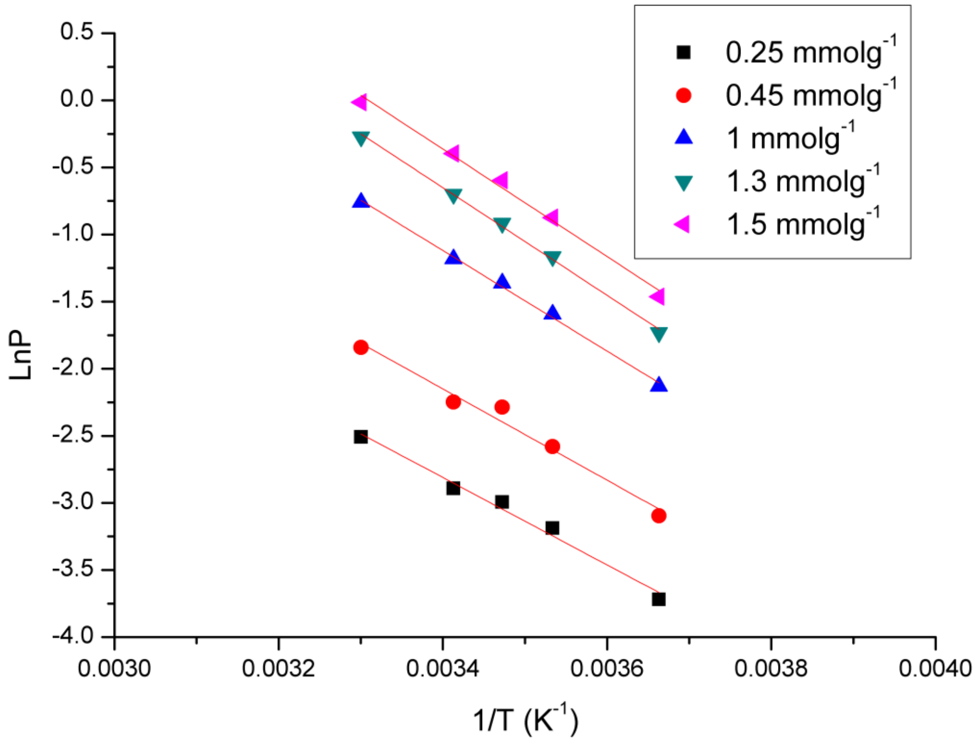

**Uptake** (mmolg^{-1})	**Slope**	**Intercept**	**σ (slope) as fraction**	**Isosteric heat of adsorption (kJ mol^{-1})**
0.25	-3281.000	8.340	0.07	27 ± 2
0.45	-3425.000	9.500	0.09	29 ± 2
1	-3774.300	11.710	0.03	31.4 ± 0.9
1.3	-4038.600	13.073	0.02	33.6 ± 0.8
1.5	-4032.200	13.340	0.05	33.5 ± 1.7

Mean value for the isosteric heat of adsorption = 30.9 ± 2.9 kJ mol^{-1}

S9 In situ single crystal polarisation experiments

S9a Samples with no CO_2

Crystal 5 (original nomenclature from experiment)

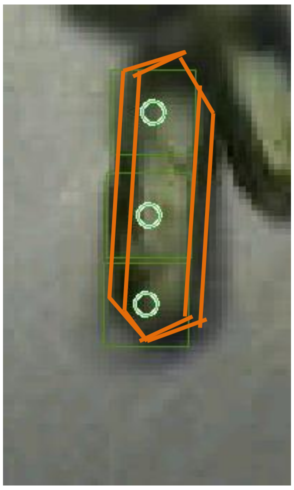

Crystal site 8 (middle).

Angle from horizontal = 84°

Crystallographic face orientation (i.e. face parallel to surface of window of the Linkam cell, and so perpendicular to the IR beam) is (0 1 1) (see S2)

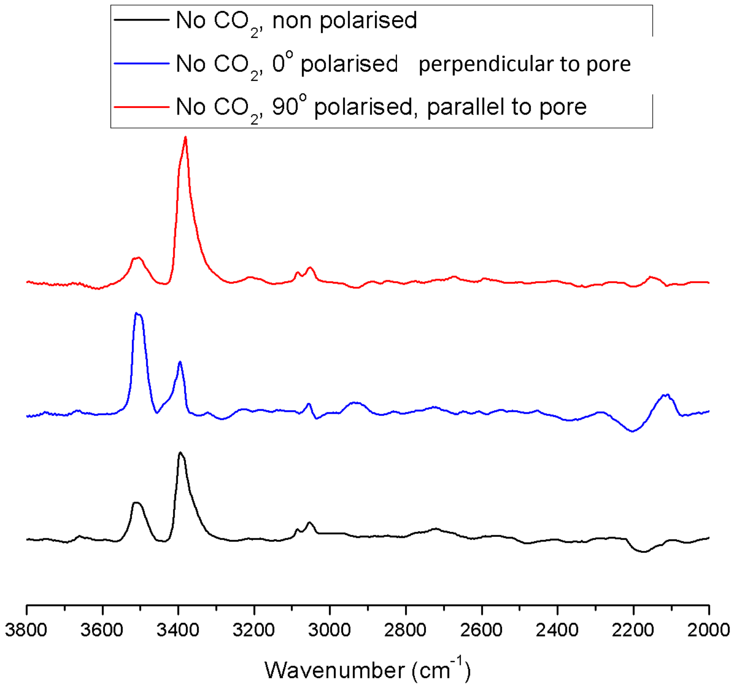

S9b Samples with CO_2

Crystal 1

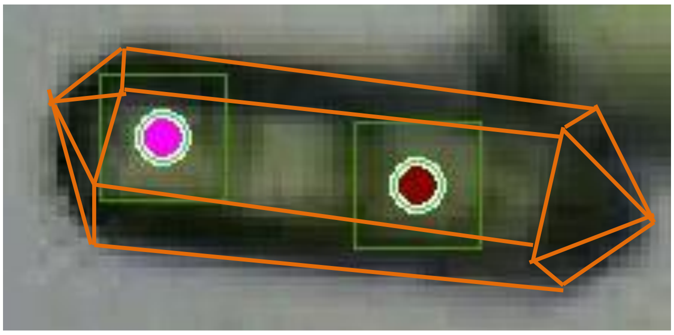

Crystal site 1(pink), site 2 (red).

Angle from horizontal = 6°.

Crystallographic face orientation = (0 1 1)

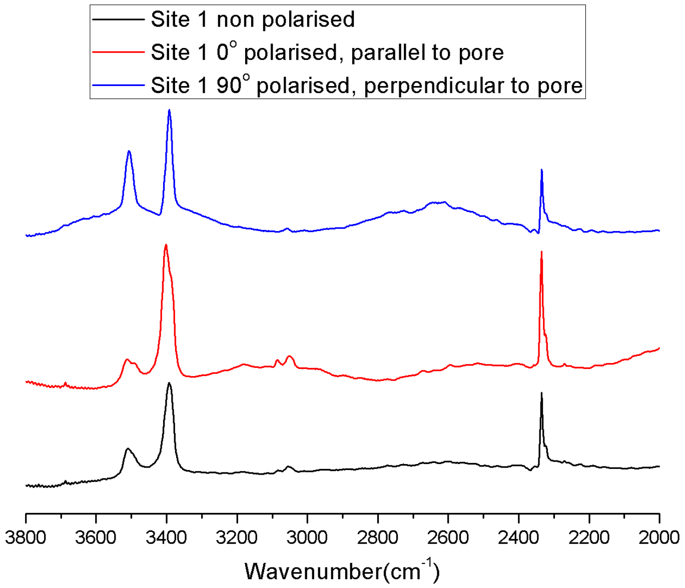

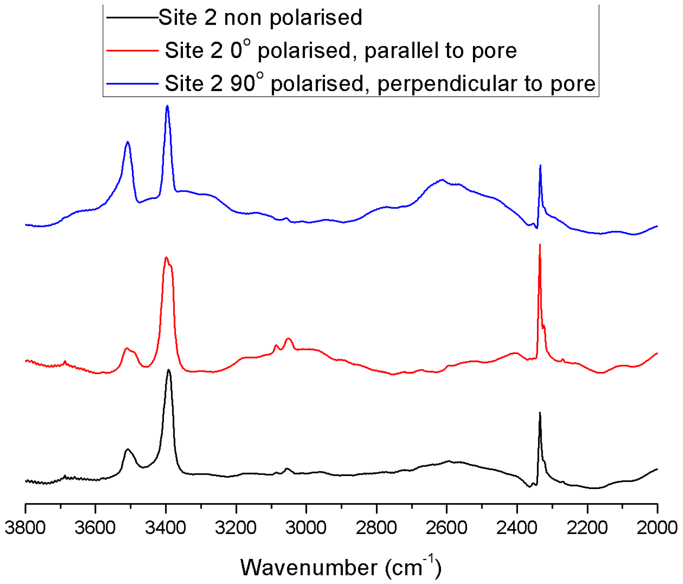

Crystal 2

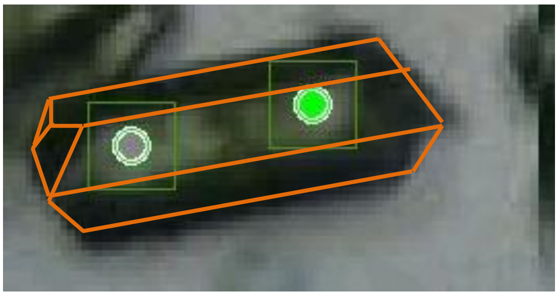

Crystal site 3 (grey), site 4 (green).

Angle from horizontal = 13°

Crystallographic face orientation = (0 1 1)

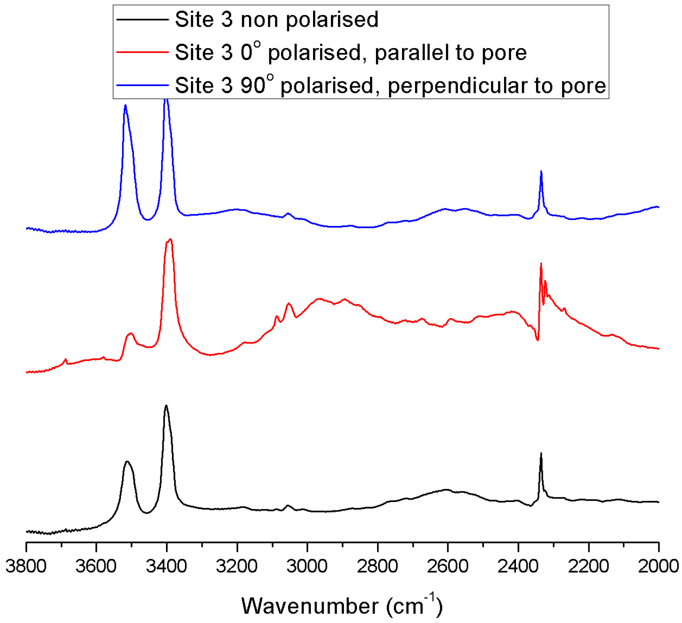

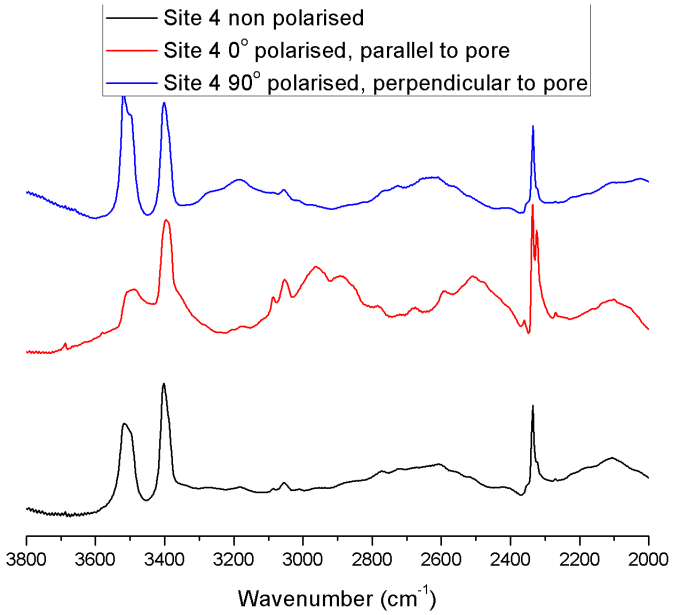

Crystal 3

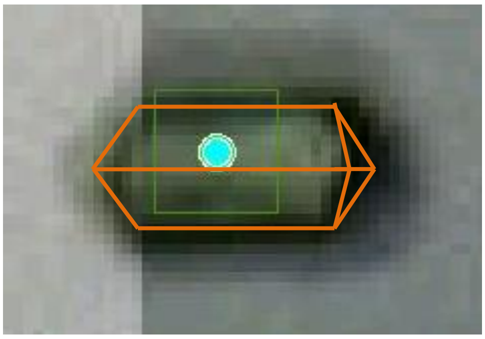

Crystal site 5 (blue).

Angle from horizontal = 0°

Crystallographic face orientation = (0 1 0)

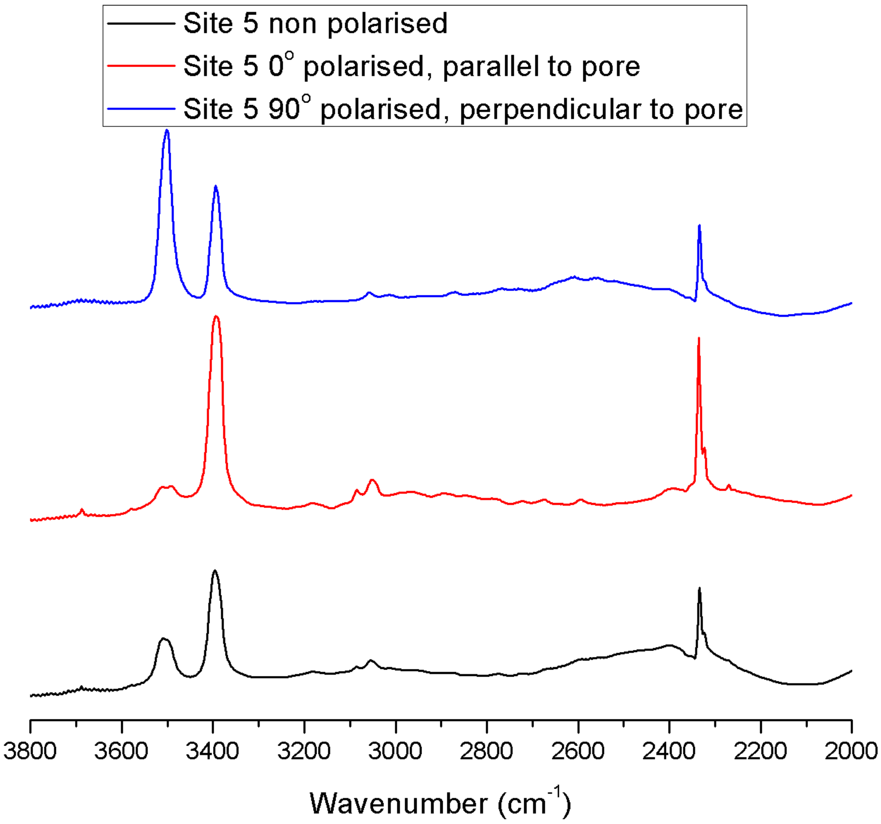

Crystal 4

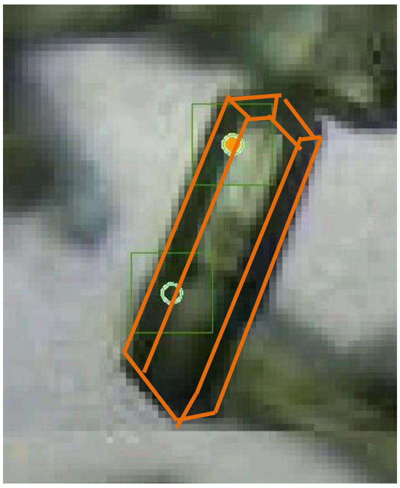

Crystal site 6 (orange).

Angle from horizontal = 71°

Crystallographic face orientation = (0 0 1)

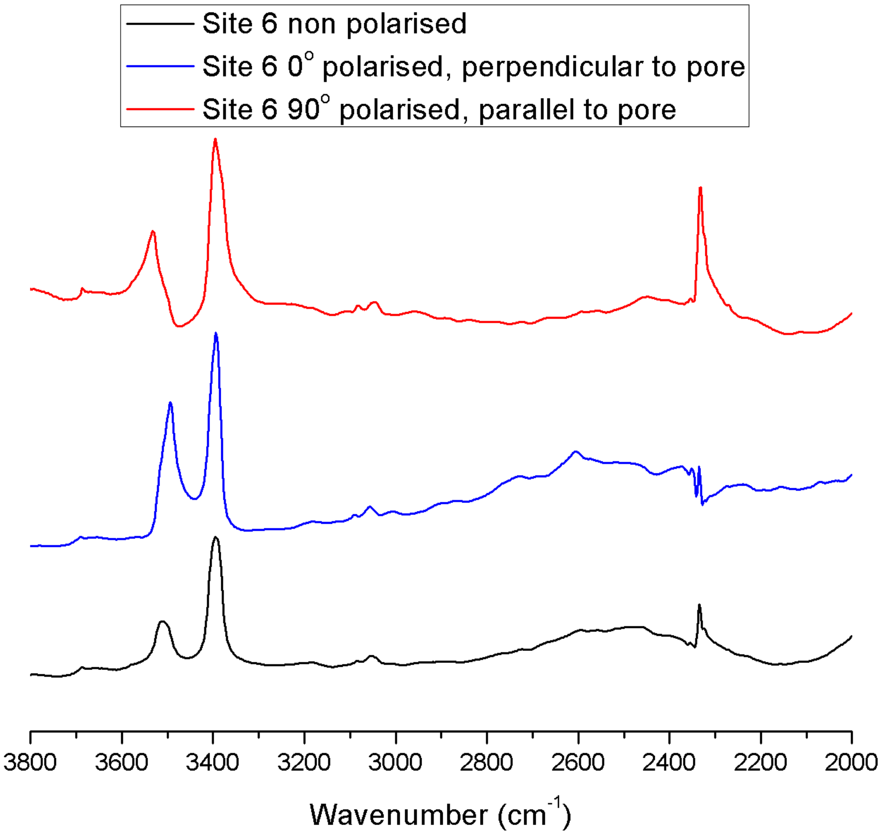

Crystal 5

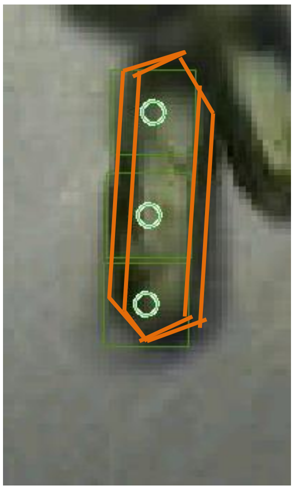

Crystal site 7 (top), site 8 (middle), site 9 (bottom).

Angle from horizontal = 84°

Crystallographic face orientation = (0 1 1)

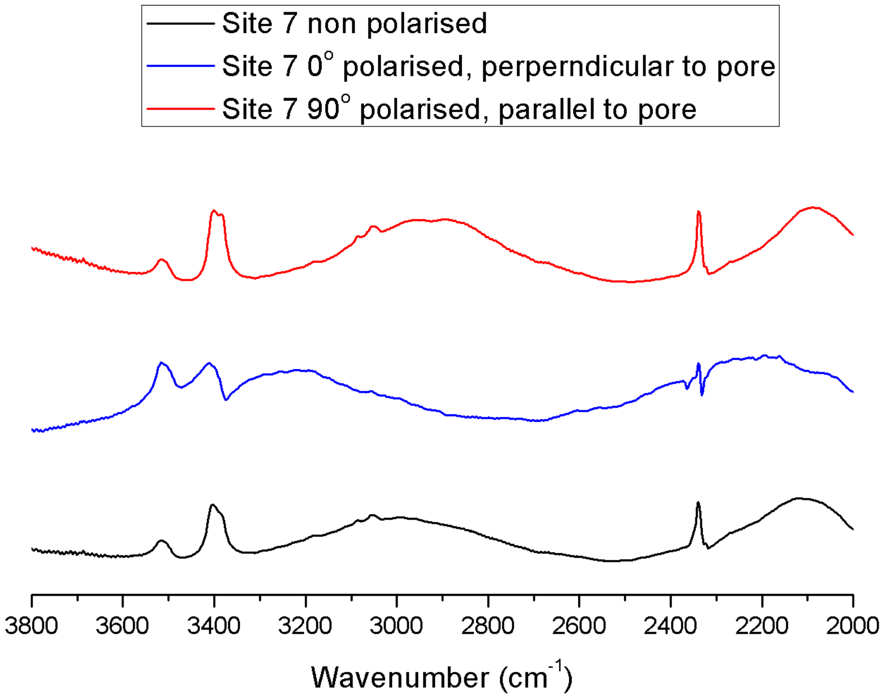

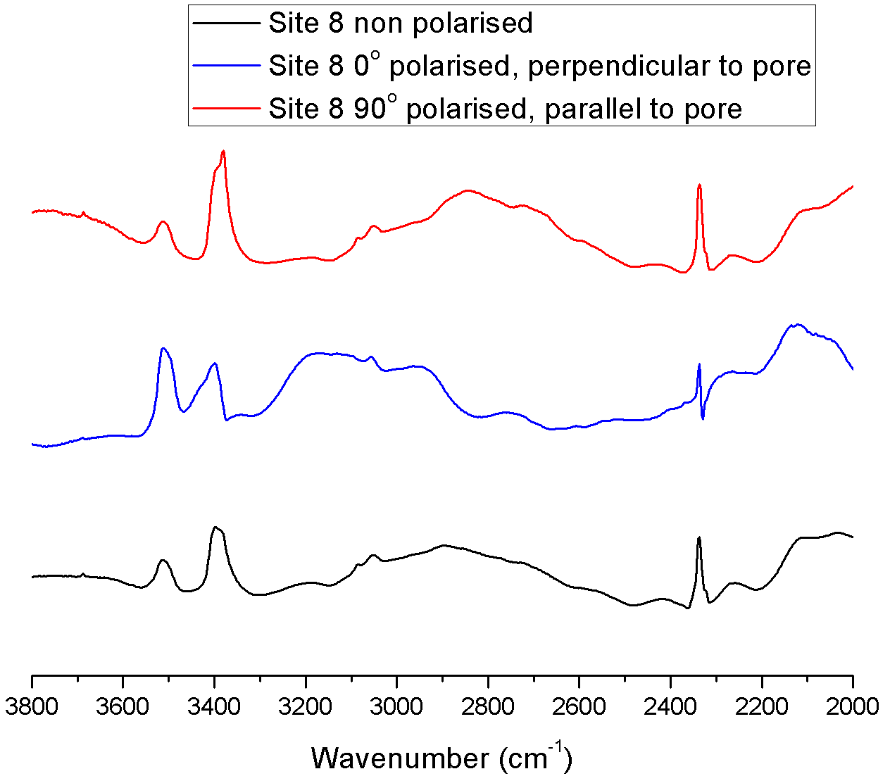
Site 8 non polarised
Site 8 0° polarised, perpendicular to pore
Site 8 90° polarised, parallel to pore
3800
3600
3400
3200
3000
2800
2600
2400
2200
2000
Wavenumber (cm-1)

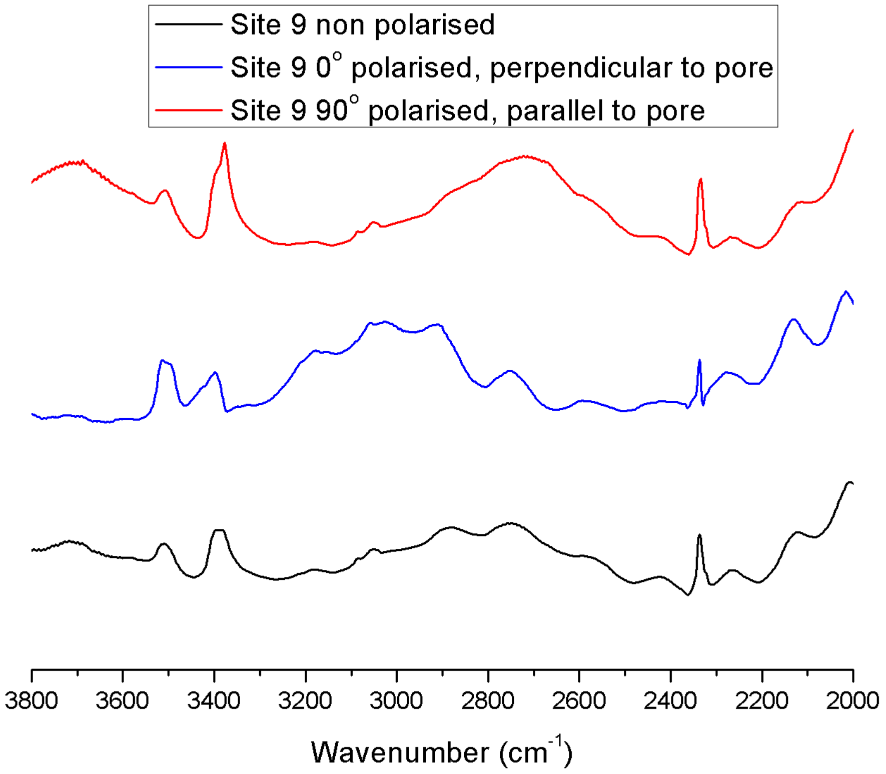
Site 9 non polarised
Site 9 0° polarised, perpendicular to pore
Site 9 90° polarised, parallel to pore
3800
3600
3400
3200
3000
2800
2600
2400
2200
2000
Wavenumber (cm-1)

Crystal 6

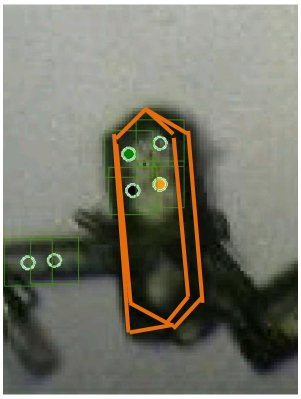

Crystal site 10 (green), site 11 (black).

Angle from horizontal = 84°

Crystallographic face orientation = (0 1 1)

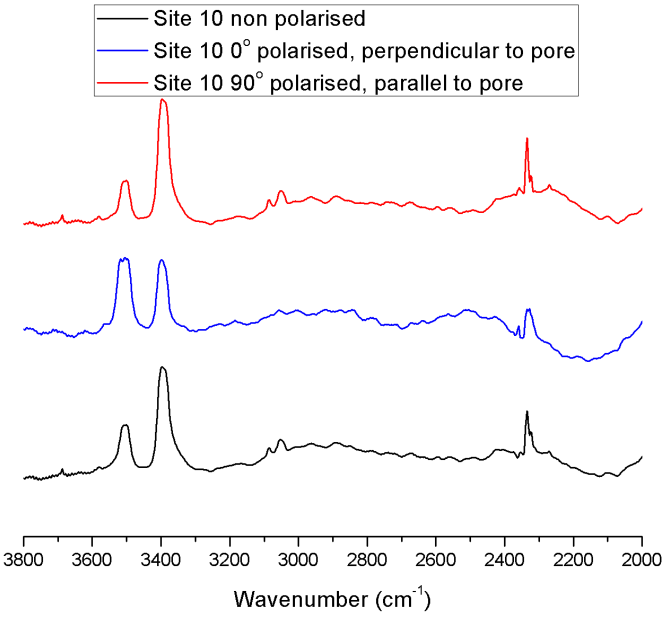

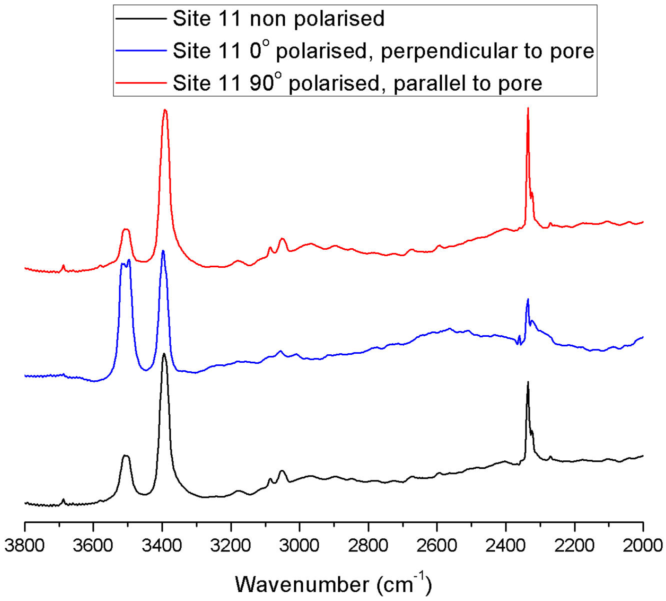

S9c Analysis of spectra

IR spectra of samples with and without CO_2 loading were measured to determine whether the structure of the MOF changes in response to CO_2 loading in a way that would affect the validity of the model used to calculate the approximate position of CO_2 loaded in the MOF. This was achieved by taking the integrals for the NH_2 symmetric and asymmetric stretches from the same crystal sites with both CO_2 present and not present, and then comparing the integrals (Table S5d-1). The peak shapes and ratios of integrals of asymmetric and symmetric stretches from the non-polarised IR spectra are similar with or without CO_2, implying no strong hydrogen bonds are formed between the NH_2 and adsorbed CO_2.

For the polarised spectra the symmetric stretches for the 90° polarised spectra, parallel to the pores, are similar for spectra with and without CO_2, indicating the average C-N vector (used later to calculate the orientation of the CO_2 molecules) does not change. In the 0° polarised spectra, the integral of the asymmetric stretch decreases between the sample with no CO_2 and the sample with CO_2. This could be due to a sigma bond rotation of the $C_{aromatic}$---N_{amine} and therefore does not affect the orientation of the $C_{aromatic}$---N_{amine} within the crystal.

Crystal 5 site	Without CO_2 loading			With CO_2 loading		
	Asymmetric stretch	Symmetric stretch	Ratio Asym/Symm	Asymmetric stretch	Symmetric stretch	Ratio Asym/Symm
Non polarised						
7	8.369	30.236	0.277	6.283	24.751	0.254

8	14.755	33.505	0.440	13.207	30.143	0.438
9	16.078	31.865	0.505	11.139	25.967	0.429
0° polarised						
7	21.346	11.875	1.798	9.370	12.078	0.776
8	31.543	13.592	2.321	15.864	13.386	1.185
9	24.404	9.842	2.480	14.3	9.288	1.540
90° polarised						
7	5.376	32.578	0.165	6.168	32.59	0.189
8	11.381	57.214	0.199	8.621	43.734	0.197
9	14.016	60.777	0.231	9.189	41.169	0.223

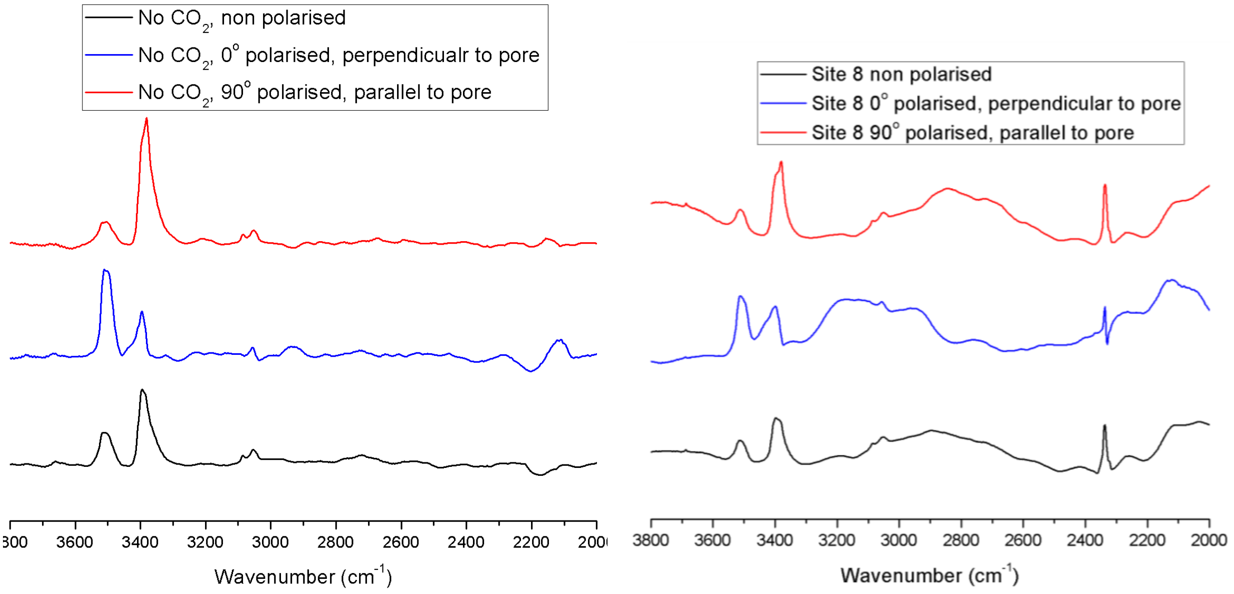

Analysis of the spectra of MOF crystals loaded with CO_2 was used to determine positional information about the CO_2 molecules with in the pores of the MOF framework. The analysis method used for determining positional information of adsorbed CO_2 molecules within the pores of $Sc_2(BDC\text{-}NH_2)_3$ was to integrate the NH_2 symmetric stretch and the CO_2 asymmetric stretch in each of the non-polarised, 0° polarised and 90° polarised spectra. For each spectra a CO_2 : $NH_{2symmetric}$ ratio was calculated.[9] The ratios for the 0° polarised and 90° polarised spectra was then compared with the ratio calculated for the non-polarised spectra as

this enabled the investigation of whether or not the CO_2 stretch had been enhanced or suppressed relative to the $NH_{2symmetric}$ stretch in each polarisation of radiation.

Crystal	1					
Orientation (face) : relative to 0° polarisation	(0 1 1) : Parallel					
Site	1			2		
Polarisation	None	0°	90°	None	0°	90°
NH_2 Symmetric Integral	42.45	60.45	28.78	35.82	60.01	26.44
CO_2 Asymmetric Integral	9.83	16.46	6.17	9.45	17.11	6.05
CO_2 / NH_2 Ratio	0.23	0.27	0.21	0.26	0.28	0.23
Enhancement		1.18	0.93		1.08	0.87

Crystal	2					
Orientation (face) : relative to 0° polarisation	(0 1 1) : Parallel					
Site	3			4		
Polarisation	None	0°	90°	None	0°	90°
NH_2 Symmetric Integral	50.67	64.53	53.00	39.41	42.96	35.13
CO_2 Asymmetric Integral	6.14	9.54	5.32	10.29	18.55	7.76
CO_2 / NH_2 Ratio	0.12	0.15	0.10	0.26	0.43	0.22
Enhancement		1.22	0.83		1.65	0.85

Crystal	3		
Orientation (face) : relative to 0° polarisation	(0 1 0) : Parallel		
Site	5		
Polarisation	None	0°	90°
NH_2 Symmetric Integral	31.52	57.12	24.24
CO_2 Asymmetric Integral	7.22	10.97	6.24
CO_2 / NH_2 Ratio	0.23	0.20	0.26
Enhancement		0.84	1.12

Crystal	4		
Orientation (face) : relative to 0° polarisation	(0 0 1) : Perpendicular		
Site	6		
Polarisation	None	0°	90°
NH_2 Symmetric Integral	40.02	44.59	70.66
CO_2 Asymmetric Integral	3.94	1.77	13.44
CO_2 / NH_2 Ratio	0.10	0.04	0.19
Enhancement		0.40	1.93

Crystal	5								
Orientation (face) : relative to 0° polarisation	(0 1 1) : Perpendicular								
Site	7			8			9		
Polarisation	None	0°	90°	None	0°	90°	None	0°	90°
NH_2 Symmetric Integral	24.77	17.74	34.74	31.19	21.33	41.28	26.07	19.20	37.05
CO_2 Asymmetric Integral	7.19	3.22	10.03	9.42	3.31	11.63	8.06	4.07	10.49
CO_2 / NH_2 Ratio	0.29	0.18	0.29	0.30	0.16	0.28	0.31	0.21	0.28
Enhancement		0.63	0.99		0.51	0.93		0.69	0.92

Crystal	6					
Orientation (face) : relative to 0° polarisation	(0 1 1) : Perpendicular					
Site	10			11		
Polarisation	None	0°	90°	None	0°	90°
NH_2 Symmetric Integral	56.33	32.64	64.26	67.14	46.83	73.99
CO_2 Asymmetric Integral	7.35	2.38	9.74	11.90	7.037	14.51
CO_2 / NH_2 Ratio	0.13	0.07	0.15	0.18	0.15	0.20
Enhancement		0.56	1.16		0.85	1.11

Analysis of the polarised spectra shows that the size of the NH_2 symmetric stretch changes with respect to the non-polarised IR spectra. The area of the peak increases for polarised spectra that are parallel with the long axis of the crystal (0° polarised IR spectra for crystals parallel to 0° polarisation and 90° polarised spectra for crystals perpendicular to 0° polarisation) and decreases for spectra in which the polarisation is perpendicular to the long axis of the crystal (90° polarised IR spectra for crystals parallel to 0° polarisation and 0° polarised spectra for crystals perpendicular to 0° polarisation). The only exceptions to this pattern are for Crystal 2 (site 3) where the 90° polarised spectra has a larger integral for the NH_2 symmetric stretch than in the non-polarised spectra and in crystal 4 (site 6) where the 0° polarised spectra has a larger integral than the non-polarised for the NH_2 symmetric stretch. In both of these crystals the angle that the physical crystal is orientated away from the idealised 0° and 90° polarisation of the IR beam is relatively high $>13^o$ and therefore the suppression / enhancement effects for the NH_2 stretch is not as well defined when compared to crystals more closely orientated to the direction of polarisation.

The same pattern of behaviour is exhibited by the peaks associated with the CO_2 asymmetric stretch whereby the peak is (i) enhanced between the non-polarised IR and the polarised spectra measured where the direction of polarisation runs parallel to the long axis of the crystal (0° polarised IR spectra for crystals parallel to 0° polarisation and 90° polarised spectra for crystals perpendicular to 0° polarisation) and (ii) decreases in spectra in which the polarisation is perpendicular to the long axis of the crystal (90° polarised IR spectra for crystals parallel to 0° polarisation and 0° polarised spectra for crystals perpendicular to 0° polarisation). There are no exceptions to this in the data.

From the pattern of behaviour exhibited by the size of the integrals for the CO_2 asymmetric stretch being the same as that for the NH_2 symmetric stretch it can be concluded that the orientation of the CO_2 molecules is approximately parallel to the dipole vector for the NH_2 symmetric stretch.

S9d Crystal projections onto (011), (001) and (010) planes

Projections of the structure down each of the observed orientations of the crystals were used to analyse the single crystal polarised IR spectra. For each projection down a plane normal the representation of one full pore of the structure has been constructed from the single crystal crystallographic information file. The crystal structure shows that there are two inequivalent NH_2 functionalised benzene di carboxylates present in the structure.

Two thirds of the amine-functionalised benzenedicarboxylates (linker 2) are present in a disordered form where the amine N atoms are split over two positions and have 50% occupancy. These amino N atoms are represented as purple spheres in the projections.

The remaining one third of the amine-functionalised benzene dicarboxylates are present in a disordered form where the amine N atoms are disordered over 4 positions with 25% occupancy. These amine N atoms are represented as green spheres in the projections.

Projection onto (011)

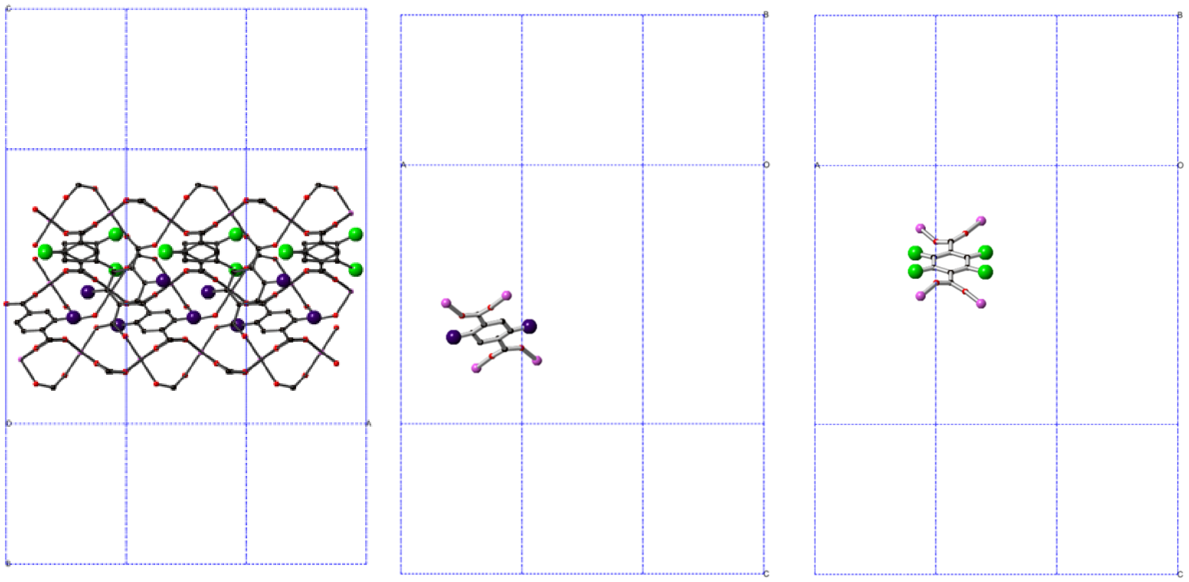

2 / 3 BDC-NH_2 (purple spheres) angle of C-N bond from horizontal ≈ 6°

1 / 3 BDC-NH_2 (green spheres) angle of C-N bond from horizontal ≈ ½ 28°, ½ 6°

Net dipole moment perpendicular to pore $= (\frac{2}{3} * (\sin 11.5) + \frac{1}{6} * (\sin 28) + \frac{1}{6} * (\sin 6))$

$= 0.165x$

Net dipole moment parallel to pore $= (\frac{2}{3} * (\cos 11.5) + \frac{1}{6} * (\cos 28) + \frac{1}{6} * (\cos 6))$

$= 0.976\,x$

Net angle of NH_2 symmetric stretch away from horizontal = 12.60^{o}

Projection onto (0 0 1)

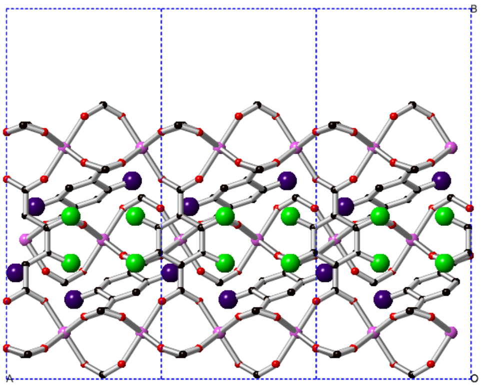

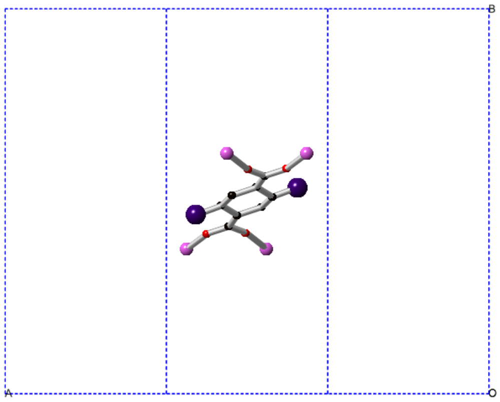

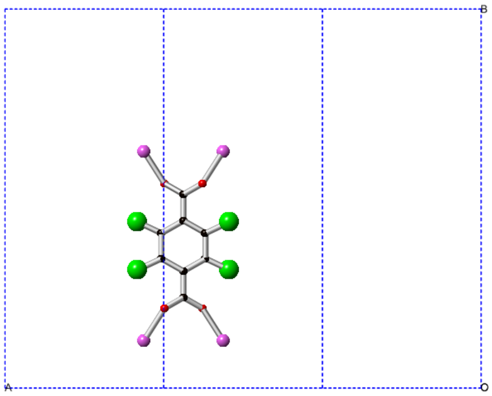

2 / 3 BDC-NH_2 (purple spheres) angle of C-N bond from horizontal ≈ 15°

1 / 3 BDC-NH_2 (green spheres) angle of C-N bond from horizontal ≈ 19°

Net dipole moment perpendicular to pore $= (\frac{2}{3} * (\sin 15) + \frac{1}{3} * (\sin 19))$

$$= 0.282x$$

Net dipole moment parallel to pore $= (\frac{2}{3} * (\cos 15) + \frac{1}{3} * (\cos 19))$

$$= 0.929\, x$$

Net angle of NH_2 symmetric stretch away from horizontal =16.44^{o}

Projection onto (0 1 0)

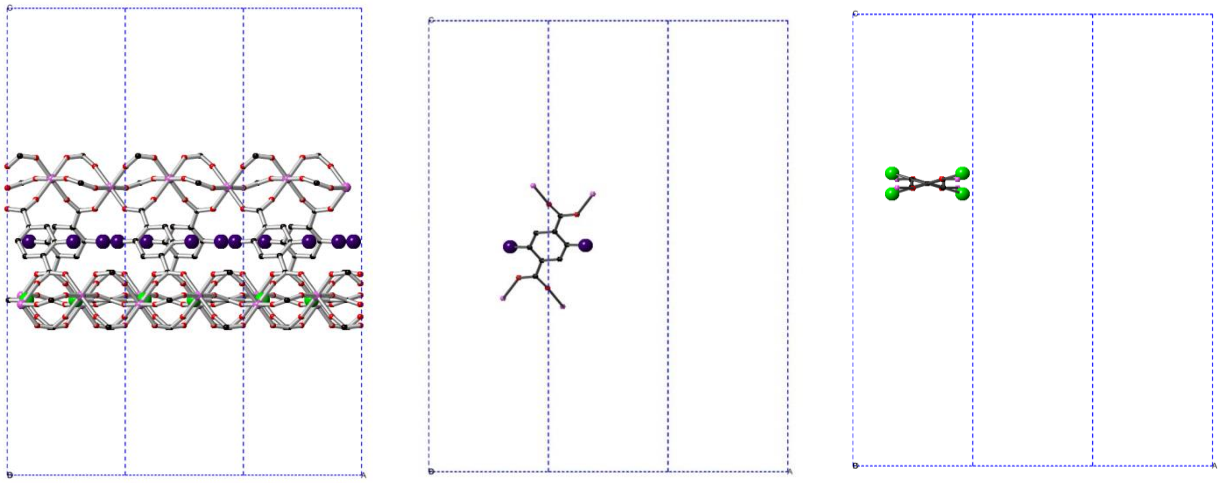

2/3 BDC-NH_2 (purple spheres) angle of C-N bond from horizontal ≈ 2°

1/3 BDC-NH_2 (green spheres) angle of C-N bond from horizontal ≈ 18°

Net dipole moment perpendicular to pore $= (\frac{2}{3} * (\sin 1) + \frac{1}{3} * (\sin 18))$

$$= 0.126\,x$$

Net dipole moment parallel to pore $= (\frac{2}{3} * (\cos 1) + \frac{1}{3} * (\cos 0))$

$$= 0.983x$$

Net angle of NH_2 symmetric stretch away from horizontal = 10.49^o

S9e Interpretation of spectra and crystal projections: Positioning of adsorbed CO_2

Using the information obtained from the crystal projections and the polarised IR spectra it is possible to estimate the orientation of the CO_2 molecules in the pores of $Sc_2(BDC\text{-}NH_2)_3$. From the polarised IR spectra it is apparent that the CO_2 asymmetric stretch is enhanced relative to the NH_2 symmetric stretch in crystals that lie with either their (001) or their (011) crystal planes flat on the Linkam cell windows (and therefore perpendicular to the IR beam), while it is suppressed for those that lie with their (010) planes parallel to the window. This would suggest that the net dipole moment associated with the asymmetric stretch of CO_2 is more closely aligned with the pores of the MOF than the net dipole moment of the NH_2 in the samples where the CO_2 : NH_2 ratio is enhanced. The inverse argument applies in samples where the CO_2 : NH_2 ratio is suppressed, i.e. the net dipole moment of the CO_2 is less aligned with the pores of the MOF than the net dipole moment of the NH_2.

As the net dipole of the CO_2 asymmetric stretch is directly parallel with the long dimension of the linear molecule the result makes the direct comparison of the adsorbed CO_2 molecules with the net angle of the NH_2 groups away from the horizontal direction possible. As the ratio of CO_2 : NH_2 signals is enhanced in both the (011) and (001) scenarios, it can be inferred that the long axis of the CO_2 molecules lies closer to being parallel with the pore than the average angle of the C-N bonds present in the crystal structure. This would represent being closer than 12.6^o in (011) crystals and 16.4^o for (0 0 1). In the scenario of crystals on the (010) face the of CO_2: NH_2 signal is suppressed and therefore it can be inferred that the long axis of the CO_2 molecule lies further away from the pore than the average angle of the C-N bonds present in the crystal structure, this value is 10.5^o and therefore the CO_2 molecules are not pointing directly down the pores of the MOF.

S 10 Synchrotron IR Microcrystal Spectroscopy Developments

The technique of synchrotron IR microcrystal spectroscopy has great potential to be used widely for studies of adsorption on single crystals. The photon flux density using slit sizes of 15×15 μm^2 is already $100 – 1000 \times$ that from lab sources and developments in beam properties and in environmental cell and detectors at the Diamond Light Source will further enhance the possibilities.

Diamond is designed to operate with a beam current of 500 mA - it currently works at 300 mA - and will do so in the nearest future, leading to an increase in flux, while future upgrades can increase the brightness potentially another order of magnitude above its current value. This will permit the study of smaller sample areas than described in the paper, with similar signal/noise (S/N) ratios, improving spatial resolution. Experimentally, the smallest slit size experimentally used is 5×5 μm^2 down to 1000 cm^{-1}, which now gives S/N (rms) values >5000 in 30 seconds (256 scans). It should be noted that the spatial resolution in confocal mode by the 36× objective (NA = 0.5) is diffraction-limited to the wavelength at the CO_2 symmetric stretch frequency to 4 μm.

A brighter beam will also permit spectra of with S/N ratios similar to those in this paper to be collected more rapidly, which will allow the study of heterogeneous systems or of the adsorption or desorption of gases from crystals where concentration gradients arise. Although the distribution of adsorbed CO_2 was homogeneous in the $Sc_2(BDC\text{-}NH_2)_3$ under the experimental conditions described in the paper, there are other conditions and systems where adsorbates exhibit slower diffusion and these will result in inhomogeneous distributions. This could result at lower temperatures or in solids with stronger adsorbate-adsorbent interactions. Measurement of these distributions can give anisotropic diffusivities. Future possibilities

include the use of Multipixel Focal Plane Array detectors to measure rapidly the spectra from extended areas with close to diffraction-limited resolution.[10]

In the experiment described in the paper, the Linkam cell used has a path length of 10 mm, which reduced the partial pressure of CO_2 that could be used to below *ca*. 0.3 bar, because IR absorption from the gas phase CO_2 greatly reduced the incident beam at frequencies close to that of the adsorbed CO_2. Planned development of the environmental cell to reduce this path length to a few mm or less will extend the range of partial pressures of flowing adsorbing gas that can be used. This will make the method more directly relevant to processes where higher concentrations of adsorbing gas are present.

S11 Notes and References

1. Scandium chloride (1.5 mol dm^{-3}, 100 ml) solution prepared from scandium oxide (Sc_2O_3, 99.999%, Stanford Materials Corporation, 75 mmol, 10.34 g), dissolved in hydrochloric acid (HCl, 38%, Fischer Scientific, 225 mmol, 18.6 cm^3) with heating. Upon cooling the solution is made up to 100 cm^3 with distilled water.

2. J. P. S. Mowat, S. R. Miller, J. M. Griffin, V. R. Seymour, S. E. Ashbrook, S. P. Thompson, D. Fairen-Jimenez, A.-M. Banu, T. Düren, P. A. Wright, *Inorg.Chem*. **2011**, *50*, 10844–10858

3. M. Eic, D.M. Ruthven, *Zeolites*, **1988**, *8*, 40-45.

4. Brandani, S., Z. Xu, and D. Ruthven, *Microporous Materials*, **1996**, *7*, 323-331.

5. S. Brandani, D.M. Ruthven, *Ind. Eng. Chem. Res*., **1996**, *35*, 315-319.

6. F. Brandani, D.M. Ruthven, C.G. Coe, *Ind. Eng. Chem. Res*., **2003**, *42*, 1451-1461.

7. H. D. Wang et al., *Adsorption*, **2011**, *17*, 687-694.

8. S. Brandani, D.M. Ruthven, *Adsorption*, **1996**, *2*, 133-143.

9. The integral for the NH_2 asymmetric stretch was not included in the calculation as the dipole corresponds to a change in a plane perpendicular to the C_{phenyl}-N_{amine} bond. As this analytical method does not enable the position of the NH_2 protons to be modelled it is not possible to define the specific dipole vector relating to the asymmetric stretch and therefore information from the asymmetric stretch has to be omitted from positional analysis.

10. E. Stavitski, R. J. Smith, M. W. Bourassa, A. S. Acerbo, G. L. Carr, L. M. Miller *Anal. Chem*. **2013**, *85*, 3599-3605

Cif file

```
data_global
_audit_creation_date        "2014-08-18"
_audit_creation_method CRYSTALS_ver_14.43

_oxford_structure_analysis_title  'ST3049_0m in Fddd'
_chemical_name_systematic         ?
_chemical_melting_point           ?

#looking for refcif

_publ_contact_author_name         'Stephen A. Moggach'
_publ_contact_author_address
;
 Stephen Moggach
 Lecturer in Inorganic Chamistry
 The University of Edinburgh
 Joseph Black Building
 West Mains Road
 Edinburgh
 EH9 3JJ
;

_publ_contact_author_phone        '+44(0)131 6517152'
_publ_contact_author_email        's.moggach@ed.ac.uk'


_publ_section_exptl_refinement
;
245_ALERT_2_B U(iso) H131    Smaller than U(eq) N1      by ...       0.09 AngSq
366_ALERT_2_C Short? C(sp?)-C(sp?) Bond  C5     -   C7_s  ...       1.39 Ang.
366_ALERT_2_C Short? C(sp?)-C(sp?) Bond  C6     -   C7     ...       1.38 Ang.
```

These are caused by the H-atom attached to the Carbon atom C13, which has a part-occupied H-atom attached as well as a disordered amino group which causes confusion over the hybridisation.

919_ALERT_3_B Reflection # Likely Affected by the Beamstop ...   1

A sin theta/ lamda cutoff was used in order to remove this reflection.

213_ALERT_2_C Atom O4 has ADP max/min Ratio ..... 3.20 prola
241_ALERT_2_C Check High Ueq as Compared to Neighbors for O9
242_ALERT_2_C Check Low Ueq as Compared to Neighbors for Sc1
250_ALERT_2_C Large U3/U1 Ratio for Average U(i,j) Tensor 3.10

Some slight libration does take place in the BDC ligands, however the principal axes of the thermal ellipsoids are well within normal parameters.

Principal axes of the thermal ellipsoids, A**2

		Min	Med	Max	Uarith	Ugeom	Uprime
SC	1.	0.0095	0.0097	0.0132	0.0108	0.0107	0.0135
O	2.	0.0123	0.0177	0.0596	0.0299	0.0235	0.0863
O	4.	0.0089	0.0203	0.0897	0.0396	0.0253	0.2039
O	9.	0.0133	0.0197	0.0841	0.0390	0.0280	0.1249
C	3.	0.0095	0.0175	0.0477	0.0249	0.0199	0.0879
C	5.	0.0105	0.0207	0.0587	0.0300	0.0234	0.1153
C	6.	0.0146	0.0204	0.1049	0.0467	0.0315	0.1468
C	7.	0.0128	0.0179	0.1143	0.0484	0.0297	0.1600
C	10.	0.0137	0.0187	0.0415	0.0247	0.0220	0.0566
C	11.	0.0118	0.0243	0.0536	0.0299	0.0249	0.1103
C	12.	0.0141	0.0226	0.0982	0.0450	0.0315	0.1566
C	13.	0.0161	0.0244	0.0956	0.0454	0.0335	0.1445

601_ALERT_2_C Structure Contains Solvent Accessible VOIDS of . 38 A**3

The structure is porous, though the pores are relatively small and hydrophobic, and no large difference peaks could be found within the pores.

910_ALERT_3_C Missing # of FCF Reflections Below Th(Min) 2
913_ALERT_3_C Missing # of Very Strong Reflections in FCF 1
909_ALERT_3_G Percentage of Observed Data at Theta(Max) still 71 Perc.
960_ALERT_3_G Number of Intensities with I .LT. - 2*sig(I) .. 1

===
Resolution & Completeness Statistics (Cumulative and Friedel Pairs Averaged)
===

Theta	sin(th)/Lambda	Complete	Expected	Measured	Missing
20.82	0.500	0.998	822	820	2

23.01   0.550   0.997   1097   1094   3
25.03   0.595   0.999   1391   1389   2
-- ACTA Min. Res. ---

Note: The Reported Completeness refers to the Actual H,K,L Index Range

242_ALERT_2_G Check Low   Ueq as Compared to Neighbors for   C13

This C-atom is attached to a 0.25 occupancy amino group, which shows large thermal motion.

430_ALERT_2_B Short Inter D...A Contact O2   .. O2   ..   2.84 Ang.
430_ALERT_2_A Short Inter D...A Contact O9   .. O9   ..   2.21 Ang.
430_ALERT_2_C Short Inter D...A Contact O4   .. O9   ..   2.85 Ang.
430_ALERT_2_C Short Inter D...A Contact O4   .. O4   ..   2.86 Ang.

These are oxygen oxygen contacts between carboxyl oxygen atoms within an octahedral
ScO6 unit. This would appear to be an erroneous alert.

301_ALERT_3_G Note: Main Residue Disorder ....   16 Perc.

This is correct (see above)

432_ALERT_2_G Short Inter X...Y Contact O2   .. C7   ..   2.83 Ang.
432_ALERT_2_G Short Inter X...Y Contact O9   .. C13   ..   2.78 Ang.
432_ALERT_2_G Short Inter X...Y Contact N8   .. C5   ..   2.51 Ang.
432_ALERT_2_G Short Inter X...Y Contact N8   .. C3   ..   3.00 Ang.
432_ALERT_2_G Short Inter X...Y Contact C3   .. C7   ..   2.51 Ang.
432_ALERT_2_G Short Inter X...Y Contact C5   .. C7   ..   1.39 Ang.
432_ALERT_2_G Short Inter X...Y Contact C5   .. C6   ..   2.38 Ang.
432_ALERT_2_G Short Inter X...Y Contact C5   .. C5   ..   2.78 Ang.
432_ALERT_2_G Short Inter X...Y Contact C6   .. C7   ..   2.40 Ang.
432_ALERT_2_G Short Inter X...Y Contact C6   .. C6   ..   2.73 Ang.
432_ALERT_2_G Short Inter X...Y Contact C7   .. C7   ..   2.80 Ang.
432_ALERT_2_G Short Inter X...Y Contact C10   .. C13   ..   2.49 Ang.
432_ALERT_2_G Short Inter X...Y Contact C10   .. C13   ..   2.49 Ang.
432_ALERT_2_G Short Inter X...Y Contact C10   .. C12   ..   2.50 Ang.
432_ALERT_2_G Short Inter X...Y Contact C11   .. C13   ..   1.38 Ang.
432_ALERT_2_G Short Inter X...Y Contact C11   .. C13   ..   1.38 Ang.
432_ALERT_2_G Short Inter X...Y Contact C11   .. C12   ..   1.38 Ang.

432_ALERT_2_G Short Inter X...Y Contact C11 .. C12 .. 2.40 Ang.
432_ALERT_2_G Short Inter X...Y Contact C11 .. C12 .. 2.40 Ang.
432_ALERT_2_G Short Inter X...Y Contact C11 .. C13 .. 2.41 Ang.
432_ALERT_2_G Short Inter X...Y Contact C11 .. N1 .. 2.67 Ang.
432_ALERT_2_G Short Inter X...Y Contact C11 .. N1 .. 2.67 Ang.
432_ALERT_2_G Short Inter X...Y Contact C11 .. C11 .. 2.78 Ang.

These are 1,3 contacts within the BDC ligands. This would appear to be an erroneous alert.

760_ALERT_1_G CIF Contains no Torsion Angles .... ?
779_ALERT_4_G Suspect or Irrelevant (Bond) Angle in CIF .... # 33
N8 -C7 -H71 1.555 1.555 1.555 5.10 Deg.
779_ALERT_4_G Suspect or Irrelevant (Bond) Angle in CIF .... # 39
C12 -C11 -C13 18.656 1.555 27.654 25.60 Deg.
779_ALERT_4_G Suspect or Irrelevant (Bond) Angle in CIF .... # 46
C13 -C11 -C12 10.566 1.555 1.555 25.60 Deg.
779_ALERT_4_G Suspect or Irrelevant (Bond) Angle in CIF .... # 58
N1 -C13 -H131 1.555 1.555 1.555 10.40 Deg.
779_ALERT_4_G Suspect or Irrelevant (Bond) Angle in CIF .... # 62
C13 -N1 -H131 1.555 1.555 1.555 15.40 Deg.
779_ALERT_4_G Suspect or Irrelevant (Bond) Angle in CIF .... # 68
C13 -H131 -C12 1.555 1.555 10.566 28.00 Deg.
804_ALERT_5_G ARU-Pack Problem in PLATON Analysis .... 1 Times

No action taken.

808_ALERT_5_G No Parseable SHELXL Style Weighting Scheme Found !

Shelx was not used.

811_ALERT_5_G No ADDSYM Analysis: Too Many Excluded Atoms .... !

The symmetry has been checked.

860_ALERT_3_G Note: Number of Least-Squares Restraints 46

One of the BDC ligands is disordered about a 2-fold axis, whilst occupational disorder occurs with the amino groups. This has been modelled appropriately.

929_ALERT_5_G No Weight Pars,Obs and Calc R1,wR2,S not checked !

This has been checked.
;

_publ_section_exptl_prep
Brief details or a reference. Include solvent if known
;
?
;

#end of refcif
_cell_length_a 8.7138(3)
_cell_length_b 20.8204(7)
_cell_length_c 34.3633(12)
_cell_angle_alpha 90
_cell_angle_beta 90
_cell_angle_gamma 90
_cell_volume 6234.4(4)

_symmetry_cell_setting 'orthorhombic'
_symmetry_space_group_name_H-M 'F d d d '
_symmetry_space_group_name_Hall '-F 2uv 2vw'
loop_
 _symmetry_equiv_pos_as_xyz
 'x,y,z'
 '-x,-y,-z'
 'x,y+1/2,z+1/2'
 '-x,-y+1/2,-z+1/2'
 'x+1/2,y,z+1/2'
 '-x+1/2,-y,-z+1/2'
 'x+1/2,y+1/2,z'
 '-x+1/2,-y+1/2,-z'
 '-x,y+1/4,z+1/4'
 'x,-y+1/4,-z+1/4'
 '-x,y+3/4,z+3/4'
 'x,-y+3/4,-z+3/4'
 '-x+1/2,y+1/4,z+3/4'
 'x+1/2,-y+1/4,-z+3/4'
 '-x+1/2,y+3/4,z+1/4'
 'x+1/2,-y+3/4,-z+1/4'

```
'x+1/4,-y,z+1/4'
'-x+1/4,y,-z+1/4'
'x+1/4,-y+1/2,z+3/4'
'-x+1/4,y+1/2,-z+3/4'
'x+3/4,-y,z+3/4'
'-x+3/4,y,-z+3/4'
'x+3/4,-y+1/2,z+1/4'
'-x+3/4,y+1/2,-z+1/4'
'-x+1/4,-y+3/4,z+1/2'
'x+1/4,y+3/4,-z+1/2'
'-x+1/4,-y+5/4,z+1'
'x+1/4,y+5/4,-z+1'
'-x+3/4,-y+3/4,z+1'
'x+3/4,y+3/4,-z+1'
'-x+3/4,-y+5/4,z+1/2'
'x+3/4,y+5/4,-z+1/2'

loop_
_atom_type_symbol
_atom_type_scat_dispersion_real
_atom_type_scat_dispersion_imag
_atom_type_scat_Cromer_Mann_a1
_atom_type_scat_Cromer_Mann_b1
_atom_type_scat_Cromer_Mann_a2
_atom_type_scat_Cromer_Mann_b2
_atom_type_scat_Cromer_Mann_a3
_atom_type_scat_Cromer_Mann_b3
_atom_type_scat_Cromer_Mann_a4
_atom_type_scat_Cromer_Mann_b4
_atom_type_scat_Cromer_Mann_c
_atom_type_scat_source
 C      0.0033   0.0016   2.3100  20.8439   1.0200  10.2075   1.5886   0.5687
   0.8650  51.6512   0.2156 'International Tables Vol C 4.2.6.8 and 6.1.1.4'
 H      0.0000   0.0000   0.4930  10.5109   0.3229  26.1257   0.1402   3.1424
   0.0408  57.7998   0.0030 'International Tables Vol C 4.2.6.8 and 6.1.1.4'
 N      0.0061   0.0033  12.2126   0.0057   3.1322   9.8933   2.0125  28.9975
   1.1663   0.5826 -11.5290 'International Tables Vol C 4.2.6.8 and 6.1.1.4'
 O      0.0106   0.0060   3.0485  13.2771   2.2868   5.7011   1.5463   0.3239
   0.8670  32.9089   0.2508 'International Tables Vol C 4.2.6.8 and 6.1.1.4'
 Sc     0.2519   0.3716   9.1890   9.0213   7.3679   0.5729   1.6409 136.1080
   1.4680  51.3531   1.3329 'International Tables Vol C 4.2.6.8 and 6.1.1.4'
```

_cell_formula_units_Z 8

Given Formula = C24 H12 N3 O12 Sc2
Dc = 1.33 Fooo = 2544.00 Mu = 4.94 M = 156.07
Found Formula = C24 H15 N3 O12 Sc2
Dc = 1.34 FOOO = 2544.00 Mu = 4.95 M = 156.83

_chemical_formula_sum 'C24 H15 N3 O12 Sc2'
_chemical_formula_moiety 'C24 H15 N3 O12 Sc2'
_chemical_compound_source ?
_chemical_formula_weight 627.31

_cell_measurement_reflns_used 4328
_cell_measurement_theta_min 3
_cell_measurement_theta_max 25
_cell_measurement_temperature 120

_exptl_crystal_description 'block'
_exptl_crystal_colour 'yellow'
_exptl_crystal_size_min 0.050
_exptl_crystal_size_mid 0.070
_exptl_crystal_size_max 0.100

_exptl_crystal_density_diffrn 1.337
_exptl_crystal_density_meas ?
_exptl_crystal_density_method 'not measured'
Non-dispersive F(000):
_exptl_crystal_F_000 2544
_exptl_absorpt_coefficient_mu 0.495

Sheldrick geometric approximatio 0.97 0.98
_exptl_absorpt_correction_type multi-scan
_exptl_absorpt_process_details 'SADABS (Siemens, 1996)'
_exptl_absorpt_correction_T_min 0.90
_exptl_absorpt_correction_T_max 0.98
_diffrn_measurement_device_type 'Bruker Kappa Apex2'
_diffrn_measurement_device 'Area'
_diffrn_radiation_monochromator 'graphite'

```
_diffrn_radiation_type           'Mo K\a'
_diffrn_radiation_wavelength          0.71073
_diffrn_measurement_method       \w

# If a reference occurs more than once, delete the author
# and date from subsequent references.
_computing_data_collection       'Apex2 (Bruker AXS, 2006)'
_computing_cell_refinement       'Apex2 (Bruker AXS, 2006)'
_computing_data_reduction        'Apex2 (Bruker AXS, 2006)'
_computing_structure_solution     'Superflip (Palatinus & Chapuis, 2007)'
_computing_structure_refinement   'CRYSTALS (Betteridge et al., 2003)'
_computing_publication_material   'CRYSTALS (Betteridge et al., 2003)'
_computing_molecular_graphics     'CAMERON (Watkin et al., 1996)'

_diffrn_standards_interval_time   .
_diffrn_standards_interval_count  .
_diffrn_standards_number         0
_diffrn_standards_decay_%        ?

_diffrn_ambient_temperature       120
_diffrn_reflns_number            14036
_reflns_number_total            1389
_diffrn_reflns_av_R_equivalents   0.072
# Number of reflections without Friedels Law is 0
# Number of reflections with Friedels Law is 1389
# Theoretical number of reflections is about 2753


_diffrn_reflns_theta_min         2.602
_diffrn_reflns_theta_max         25.025
_diffrn_measured_fraction_theta_max 0.999

_diffrn_reflns_theta_full        25.025
_diffrn_measured_fraction_theta_full 0.999


_diffrn_reflns_limit_h_min       -10
_diffrn_reflns_limit_h_max       10
_diffrn_reflns_limit_k_min       -24
_diffrn_reflns_limit_k_max       24
_diffrn_reflns_limit_l_min       -40
```

_diffrn_reflns_limit_l_max 40
_reflns_limit_h_min 0
_reflns_limit_h_max 10
_reflns_limit_k_min 0
_reflns_limit_k_max 24
_reflns_limit_l_min 0
_reflns_limit_l_max 40

_oxford_diffrn_Wilson_B_factor 0.88
_oxford_diffrn_Wilson_scale 570.24

_atom_sites_solution_primary Other #heavy,direct,difmap,geom
_atom_sites_solution_secondary difmap
_atom_sites_solution_hydrogens difmap

_refine_diff_density_min -0.69
_refine_diff_density_max 1.01

The current dictionary definitions do not cover the
situation where the reflections used for refinement were
selected by a user-defined sigma threshold

The values actually used during refinement
_oxford_reflns_threshold_expression_ref I>-10.0\s(I)
_refine_ls_number_reflns 1389
_refine_ls_number_restraints 46
_refine_ls_number_parameters 105
_oxford_refine_ls_R_factor_ref 0.0705
_refine_ls_wR_factor_ref 0.1480
_refine_ls_goodness_of_fit_ref 1.0059
_refine_ls_shift/su_max 0.0302655
_refine_ls_shift/su_mean 0.0013035

The values computed with all filters except I/sigma
_oxford_reflns_number_all 1389
_refine_ls_R_factor_all 0.0705
_refine_ls_wR_factor_all 0.1480

```
# The values computed with a 2 sigma cutoff - a la SHELX
_reflns_threshold_expression      I>2.0\s(I)
_reflns_number_gt                 1084
_refine_ls_R_factor_gt            0.0531
_refine_ls_wR_factor_gt           0.1388

# choose from: rm (reference molecule of known chirality),
# ad (anomolous dispersion - Flack), rmad (rm and ad),
# syn (from synthesis), unk (unknown) or . (not applicable).
_chemical_absolute_configuration  '.'


_refine_ls_structure_factor_coef  Fsqd
_refine_ls_matrix_type            full
_refine_ls_hydrogen_treatment     noref          #undef, noref, refall,
                                          # refxyz, refU, constr or mixed
_refine_ls_weighting_scheme       calc
_refine_ls_weighting_details
;
 Method= Modified Sheldrick
 w=1/[\s^2^(F^2^) + ( 0.08P)^2^ +71.91P]
 ,where P=(max(Fo^2^,0) + 2Fc^2^)/3
;
# Insert your own references if required - in alphabetical order
_publ_section_references
;
Betteridge, P.W., Carruthers, J.R., Cooper, R.I.,
Prout, K. & Watkin, D.J. (2003). J. Appl. Cryst. 36, 1487.

Bruker Analytical X-ray Systems, Inc., 2006. <i>Apex2</i>,
Version 2 User Manual, M86-E01078, Madison, WI.

Palatinus, L. & Chapuis, G. (2007). J. Appl. Cryst. 40, 786-790.

Siemens Industrial Automation, Inc (1996).
SADABS: Area-Detector Absorption Correction;: Madison, WI.

Watkin, D.J., Prout, C.K. & Pearce, L.J. (1996). CAMERON, Chemical
Crystallography Laboratory, Oxford, UK.
;
```

Uequiv = arithmetic mean of Ui i.e. Ueqiv = (U1+U2+U3)/3

Replace last . with number of unfound hydrogen atoms attached to an atom.

..._refinement_flags_...
. no refinement constraints S special position constraint on site
G rigid group refinement of site R riding atom
D distance or angle restraint on site T thermal displacement constraints
U Uiso or Uij restraint (rigid bond) P partial occupancy constraint

```
loop_
_atom_site_label
_atom_site_type_symbol
_atom_site_fract_x
_atom_site_fract_y
_atom_site_fract_z
_atom_site_U_iso_or_equiv
_atom_site_occupancy
_atom_site_adp_type
_atom_site_refinement_flags_posn
_atom_site_refinement_flags_adp
_atom_site_refinement_flags_occupancy
_atom_site_disorder_assembly
_atom_site_disorder_group
_atom_site_attached_hydrogens
Sc1 Sc 0.3750 0.3750 0.61550(2) 0.0107 1.0000 Uani S T . . . .
O2 O 0.2448(3) 0.41592(14) 0.57058(7) 0.0297 1.0000 Uani . . . . . .
O4 O 0.0066(3) 0.41605(16) 0.59243(8) 0.0395 1.0000 Uani . . . . . .
O9 O 0.5049(3) 0.45799(12) 0.61482(10) 0.0388 1.0000 Uani . . . . . .
C3 C 0.1049(4) 0.43010(19) 0.56720(11) 0.0248 1.0000 Uani . . . . . .
C5 C 0.0542(4) 0.4663(2) 0.53211(11) 0.0299 1.0000 Uani . . . . . .
C6 C -0.0993(5) 0.4832(3) 0.52898(13) 0.0465 1.0000 Uani . . . . . .
C7 C -0.1555(5) 0.5166(3) 0.49719(13) 0.0488 1.0000 Uani . . . . . .
C10 C 0.6250 0.4865(2) 0.6250 0.0245 1.0000 Uani S T . . . .
C11 C 0.6250 0.5582(2) 0.6250 0.0299 1.0000 Uani DS TU . . . .
C12 C 0.4922(14) 0.5918(8) 0.6334(4) 0.0449 0.5000 Uani D U . . . .
C13 C 0.4930(13) 0.6588(9) 0.6344(4) 0.0453 0.5000 Uani D U . . . .
N1 N 0.332(4) 0.6839(17) 0.6474(9) 0.145(13) 0.2500 Uiso . . . . . .
N8 N -0.3158(13) 0.5343(5) 0.4983(3) 0.081(3) 0.5000 Uiso . . . . . .
H81 H -0.3722 0.5240 0.5186 0.0894 0.5000 Uiso R . . . . .
```

H82 H -0.3569 0.5555 0.4787 0.0894 0.5000 Uiso R . . . . .
H11 H 0.3190 0.7257 0.6495 0.1396 0.2500 Uiso R . . . . .
H12 H 0.2545 0.6576 0.6509 0.1396 0.2500 Uiso R . . . . .
H61 H -0.1678 0.4716 0.5493 0.0560 1.0000 Uiso R . . . . .
H121 H 0.3998 0.5691 0.6384 0.0535 0.5000 Uiso R . . . . .
H71 H -0.2616 0.5275 0.4955 0.0580 0.5000 Uiso . . . . . .
H131 H 0.4026 0.6816 0.6416 0.0545 0.2500 Uiso . . . . . .
loop_
_atom_site_aniso_label
_atom_site_aniso_U_11
_atom_site_aniso_U_22
_atom_site_aniso_U_33
_atom_site_aniso_U_23
_atom_site_aniso_U_13
_atom_site_aniso_U_12
Sc1 0.0095(5) 0.0130(4) 0.0095(5) 0.0000 0.0000 0.0006(4)
O2 0.0147(14) 0.0504(18) 0.0241(14) 0.0175(13) -0.0007(11) 0.0049(12)
O4 0.0200(15) 0.069(2) 0.0290(16) 0.0345(15) 0.0018(12) -0.0004(14)
O9 0.0204(15) 0.0148(13) 0.081(2) 0.0054(14) -0.0105(14) -0.0034(12)
C3 0.018(2) 0.037(2) 0.0197(19) 0.0169(16) -0.0008(15) -0.0020(16)
C5 0.019(2) 0.047(3) 0.024(2) 0.0203(18) -0.0040(16) 0.0015(19)
C6 0.019(2) 0.086(4) 0.035(2) 0.036(3) 0.0041(18) 0.002(2)
C7 0.017(2) 0.093(4) 0.037(2) 0.043(3) 0.0031(18) 0.009(2)
C10 0.019(3) 0.014(2) 0.041(3) 0.0000 0.001(2) 0.0000
C11 0.025(2) 0.012(2) 0.053(4) 0.0000 0.003(3) 0.0000
C12 0.022(3) 0.020(2) 0.093(11) 0.006(8) 0.018(5) -0.003(3)
C13 0.023(3) 0.020(2) 0.093(11) 0.003(8) 0.014(6) 0.006(3)

_refine_ls_extinction_method
'None'
_oxford_refine_ls_scale 0.046839(19)
loop_
_geom_bond_atom_site_label_1
_geom_bond_site_symmetry_1
_geom_bond_atom_site_label_2
_geom_bond_site_symmetry_2
_geom_bond_distance
_geom_bond_publ_flag
Sc1 . O2 29_554 2.097(3) yes
Sc1 . O9 29_554 2.066(3) yes
Sc1 . O4 16_556 2.034(3) yes

Sc1 . O4 18_556 2.034(3) yes
Sc1 . O2 . 2.097(3) yes
Sc1 . O9 . 2.066(3) yes
O2 . C3 . 1.260(4) yes
O4 . C3 . 1.253(4) yes
O9 . C10 . 1.252(4) yes
C3 . C5 . 1.489(5) yes
C5 . C7 2_566 1.386(6) yes
C5 . C6 . 1.387(6) yes
C6 . C7 . 1.384(6) yes
C6 . H61 . 0.950 no
C7 . N8 . 1.445(12) yes
C7 . H71 . 0.954 no
C10 . C11 . 1.493(7) yes
C11 . C12 18_656 1.383(8) yes
C11 . C13 27_654 1.379(8) yes
C11 . C13 10_566 1.379(8) yes
C11 . C12 . 1.383(8) yes
C12 . H131 10_566 1.258 no
C12 . C13 . 1.394(7) yes
C12 . H121 . 0.950 no
C13 . N1 . 1.56(4) yes
C13 . H131 . 0.952 no
N1 . H11 . 0.880 no
N1 . H12 . 0.880 no
N1 . H131 . 0.646 no
N8 . H81 . 0.880 no
N8 . H82 . 0.880 no
loop_
_geom_angle_atom_site_label_1
_geom_angle_site_symmetry_1
_geom_angle_atom_site_label_2
_geom_angle_site_symmetry_2
_geom_angle_atom_site_label_3
_geom_angle_site_symmetry_3
_geom_angle
_geom_angle_publ_flag
O2 29_554 Sc1 . O9 29_554 87.05(11) yes
O2 29_554 Sc1 . O4 16_556 92.71(12) yes
O9 29_554 Sc1 . O4 16_556 88.03(13) yes
O2 29_554 Sc1 . O4 18_556 177.89(12) yes

O9 29_554 Sc1 . O4 18_556 92.89(12) yes
O4 16_556 Sc1 . O4 18_556 89.40(18) yes
O2 29_554 Sc1 . O2 . 85.19(15) yes
O9 29_554 Sc1 . O2 . 92.00(12) yes
O4 16_556 Sc1 . O2 . 177.89(12) yes
O4 18_556 Sc1 . O2 . 92.71(12) yes
O2 29_554 Sc1 . O9 . 92.00(12) yes
O9 29_554 Sc1 . O9 . 178.7(2) yes
O4 16_556 Sc1 . O9 . 92.89(12) yes
O4 18_556 Sc1 . O9 . 88.03(13) yes
O2 . Sc1 . O9 . 87.05(11) yes
Sc1 . O2 . C3 . 133.4(2) yes
Sc1 16_456 O4 . C3 . 167.2(3) yes
Sc1 . O9 . C10 . 148.3(3) yes
O2 . C3 . O4 . 122.9(3) yes
O2 . C3 . C5 . 118.7(3) yes
O4 . C3 . C5 . 118.4(3) yes
C3 . C5 . C7 2_566 122.0(4) yes
C3 . C5 . C6 . 118.5(3) yes
C7 2_566 C5 . C6 . 119.5(4) yes
C5 . C6 . C7 . 122.0(4) yes
C5 . C6 . H61 . 118.9 no
C7 . C6 . H61 . 119.1 no
C5 2_566 C7 . C6 . 118.5(4) yes
C5 2_566 C7 . N8 . 124.6(6) yes
C6 . C7 . N8 . 116.8(6) yes
C5 2_566 C7 . H71 . 120.8 no
C6 . C7 . H71 . 120.7 no
N8 . C7 . H71 . 5.1 no
O9 18_656 C10 . O9 . 123.5(5) yes
O9 18_656 C10 . C11 . 118.3(2) yes
O9 . C10 . C11 . 118.3(2) yes
C10 . C11 . C12 18_656 120.5(8) yes
C10 . C11 . C13 27_654 120.0(8) yes
C12 18_656 C11 . C13 27_654 25.6(9) yes
C10 . C11 . C13 10_566 120.0(8) yes
C12 18_656 C11 . C13 10_566 113.3(6) yes
C13 27_654 C11 . C13 10_566 120.1(17) yes
C10 . C11 . C12 . 120.5(8) yes
C12 18_656 C11 . C12 . 119.1(17) yes
C13 27_654 C11 . C12 . 113.3(6) yes

C13 10_566 C11 . C12 . 25.6(9)   yes
C11 . C12 . H131 10_566 100.4   no
C11 . C12 . C13 . 120.5(16)   yes
H131 10_566 C12 . C13 . 114.1   no
C11 . C12 . H121 . 119.7   no
H131 10_566 C12 . H121 . 53.5   no
C13 . C12 . H121 . 119.8   no
C12 . C13 . C11 10_566 119.8(16)   yes
C12 . C13 . N1 . 109.8(15)   yes
C11 10_566 C13 . N1 . 130.4(18)   yes
C12 . C13 . H131 . 120.2   no
C11 10_566 C13 . H131 . 120.0   no
N1 . C13 . H131 . 10.4   no
C13 . N1 . H11 . 118.3   no
C13 . N1 . H12 . 121.6   no
H11 . N1 . H12 . 120.0   no
C13 . N1 . H131 . 15.4   no
H11 . N1 . H131 . 102.9   no
H12 . N1 . H131 . 136.8   no
C7 . N8 . H81 . 119.8   no
C7 . N8 . H82 . 120.2   no
H81 . N8 . H82 . 120.0   no
C13 . H131 . C12 10_566 28.0   no
C13 . H131 . N1 . 154.2   no
C12 10_566 H131 . N1 . 145.8   no

```
_iucr_refine_instructions_details_constraints
;
#
# Punched on 18/08/14 at 15:10:04
#
#LIST     12
BLOCK SCALE X'S
CONT SC(1, U'S) UNTIL C(13)
CONT N(1, U[ISO])
CONT N(8, U[ISO])
RIDE C   (   6,X'S) H   (  61,X'S)
RIDE N   (   8,X'S) H   (  81,X'S) H   (  82,X'S)
RIDE C   (  12,X'S) H   ( 121,X'S)
RIDE N   (   1,X'S) H   (  11,X'S) H   (  12,X'S)
```

```
END
;


_iucr_refine_instructions_details_restraints
;
#
# Punched on 18/08/14 at 15:10:04
#
#LIST     16
DISTANCE 1.390000 , 0.010000 = C(11) TO C(12)
DISTANCE 1.390000 , 0.010000 = C(12) TO C(13)
DISTANCE 1.390000 , 0.010000 = C(13) TO C(11,-2,1,0,1,1)
REM   DELU   START (DO NOT REMOVE THIS LINE)
VIBR .0, 0.00200 = C(11) TO C(12,-3,1,1,0,1)
VIBR .0, 0.00200 = C(11) TO C(13,-2,1,0,1,1)
VIBR .0, 0.00200 = C(11) TO C(13,4,2,1,0,-1)
VIBR .0, 0.00200 = C(11) TO C(12)
VIBR .0, 0.00500 = C(13,-2,1,0,1,1) TO C(12,-3,1,1,0,1)
VIBR .0, 0.00500 = C(13,4,2,1,0,-1) TO C(12,-3,1,1,0,1)
VIBR .0, 0.00500 = C(13,4,2,1,0,-1) TO C(13,-2,1,0,1,1)
VIBR .0, 0.00500 = C(12) TO C(12,-3,1,1,0,1)
VIBR .0, 0.00500 = C(12) TO C(13,-2,1,0,1,1)
VIBR .0, 0.00500 = C(12) TO C(13,4,2,1,0,-1)
VIBR .0, 0.00200 = C(12) TO C(13)
VIBR .0, 0.00500 = C(13) TO C(11)
VIBR .0, 0.00500 = C(11,4,2,1,0,-1) TO C(12)
REM   DELU   END (DO NOT REMOVE THIS LINE)
REM   THERMSIM   START (DO NOT REMOVE THIS LINE)
U(IJ) .0, 0.04000 = C(11) TO C(12,-3,1,1,0,1)
U(IJ) .0, 0.04000 = C(11) TO C(13,-2,1,0,1,1)
U(IJ) .0, 0.04000 = C(11) TO C(13,4,2,1,0,-1)
U(IJ) .0, 0.04000 = C(11) TO C(12)
U(IJ) .0, 0.04000 = C(12) TO C(13)
REM   THERMSIM   END (DO NOT REMOVE THIS LINE)
END
;
```
